# A novel molecular signature identifies mixed subtypes in renal cell carcinoma with poor prognosis and independent response to immunotherapy

**DOI:** 10.1186/s13073-022-01105-y

**Published:** 2022-09-15

**Authors:** Florian A. Büttner, Stefan Winter, Viktoria Stühler, Steffen Rausch, Jörg Hennenlotter, Susanne Füssel, Stefan Zastrow, Matthias Meinhardt, Marieta Toma, Carmen Jerónimo, Rui Henrique, Vera Miranda-Gonçalves, Nils Kröger, Silvia Ribback, Arndt Hartmann, Abbas Agaimy, Christine Stöhr, Iris Polifka, Falko Fend, Marcus Scharpf, Eva Comperat, Gabriel Wasinger, Holger Moch, Arnulf Stenzl, Marco Gerlinger, Jens Bedke, Matthias Schwab, Elke Schaeffeler

**Affiliations:** 1 Dr. Margarete Fischer-Bosch Institute of Clinical Pharmacology, Auerbachstrasse 112, 70376 Stuttgart, Germany; 2 University of Tuebingen, Tuebingen, Germany; 3 Department of Urology, University Hospital Tuebingen, Tuebingen, Germany; 4 Department of Urology, University Hospital and Faculty of Medicine, Technische Universität Dresden, Dresden, Germany; 5 Institute of Pathology, University Hospital and Faculty of Medicine, Technische Universität Dresden, Dresden, Germany; 6 Current address: Institute of Pathology, University of Bonn, Bonn, Germany; 7 Cancer Biology & Epigenetics Group-Research Center, Portuguese Oncology Institute of Porto (CI-IPOP, IPO Porto), Porto, Portugal; 8 Department of Pathology and Molecular Immunology, Institute of Biomedical Sciences Abel Salazar-University of Porto (ICBAS-UP), Porto, Portugal; 9 Department of Pathology, Portuguese Oncology Institute of Porto (IPO Porto), Porto, Portugal; 10 Department of Urology, University of Greifswald, Greifswald, Germany; 11 Institute of Pathology, University Medicine Greifswald, Greifswald, Germany; 12 Institute of Pathology, Friedrich-Alexander-University Erlangen-Nürnberg (FAU), University Hospital, Erlangen, Germany; 13 Institute of Pathology and Neuropathology, University Hospital Tuebingen, Tuebingen, Germany; 14 Department of Pathology, Medical University of Vienna, Vienna, Austria; 15 Department of Pathology and Molecular Pathology, University Hospital Zuerich, University of Zuerich, Zuerich, Switzerland; 16 Translational cancer immunotherapy and genomics lab, Barts Cancer Institute, Queen Mary University of London, London, UK; 17 German Cancer Consortium (DKTK), Partner Site Tübingen, German Cancer Research Center (DKFZ), Heidelberg, Germany; 18 Departments of Clinical Pharmacology, Pharmacy and Biochemistry, University of Tuebingen, Tuebingen, Germany; 19 Cluster of Excellence iFIT (EXC2180) “Image-Guided and Functionally Instructed Tumor Therapies”, University of Tuebingen, Tuebingen, Germany

**Keywords:** Renal cell carcinoma, Cancer-specific survival, RCC subtypes, Gene expression deconvolution, Immunotherapy

## Abstract

**Background:**

Renal cell carcinoma (RCC) is a heterogeneous disease comprising histologically defined subtypes. For therapy selection, precise subtype identification and individualized prognosis are mandatory, but currently limited. Our aim was to refine subtyping and outcome prediction across main subtypes, assuming that a tumor is composed of molecular features present in distinct pathological subtypes.

**Methods:**

Individual RCC samples were modeled as linear combination of the main subtypes (clear cell (ccRCC), papillary (pRCC), chromophobe (chRCC)) using computational gene expression deconvolution. The new molecular subtyping was compared with histological classification of RCC using the Cancer Genome Atlas (TCGA) cohort (*n* = 864; ccRCC: 512; pRCC: 287; chRCC: 65) as well as 92 independent histopathologically well-characterized RCC. Predicted continuous subtypes were correlated to cancer-specific survival (CSS) in the TCGA cohort and validated in 242 independent RCC. Association with treatment-related progression-free survival (PFS) was studied in the JAVELIN Renal 101 (*n* = 726) and IMmotion151 trials (*n* = 823). CSS and PFS were analyzed using the Kaplan–Meier and Cox regression analysis.

**Results:**

One hundred seventy-four signature genes enabled reference-free molecular classification of individual RCC. We unambiguously assign tumors to either ccRCC, pRCC, or chRCC and uncover molecularly heterogeneous tumors (e.g., with ccRCC and pRCC features), which are at risk of worse outcome. Assigned proportions of molecular subtype-features significantly correlated with CSS (ccRCC (*P* = 4.1E − 10), pRCC (*P* = 6.5E − 10), chRCC (*P* = 8.6E − 06)) in TCGA. Translation into a numerical RCC-R(isk) score enabled prognosis in TCGA (*P* = 9.5E − 11). Survival modeling based on the RCC-R score compared to pathological categories was significantly improved (*P* = 3.6E − 11). The RCC-R score was validated in univariate (*P* = 3.2E − 05; HR = 3.02, 95% CI: 1.8–5.08) and multivariate analyses including clinicopathological factors (*P* = 0.018; HR = 2.14, 95% CI: 1.14–4.04). Heterogeneous PD-L1-positive RCC determined by molecular subtyping showed increased PFS with checkpoint inhibition versus sunitinib in the JAVELIN Renal 101 (*P* = 3.3E − 04; HR = 0.52, 95% CI: 0.36 − 0.75) and IMmotion151 trials (*P* = 0.047; HR = 0.69, 95% CI: 0.48 − 1). The prediction of PFS significantly benefits from classification into heterogeneous and unambiguous subtypes in both cohorts (*P* = 0.013 and *P* = 0.032).

**Conclusion:**

Switching from categorical to continuous subtype classification across most frequent RCC subtypes enables outcome prediction and fosters personalized treatment strategies.

**Supplementary Information:**

The online version contains supplementary material available at 10.1186/s13073-022-01105-y.

## Background

Renal cell carcinoma (RCC) is among the ten most diagnosed cancers worldwide and especially in Western countries its incidence is rising [1–4].

RCC comprises histologically defined subtypes, which differ in pathophysiology, clinical course, response to treatment, and prognosis. Major subtypes are clear cell (ccRCC), papillary (pRCC), and chromophobe (chRCC) RCC [2, 5], which originate from either proximal (ccRCC, pRCC) or distal (chRCC) parts of the nephron [2]. Disease relapse after surgery occurs in 20–40% of patients with localized RCC. Therapeutic options, mainly approved for ccRCC [3, 4, 6, 7], have recently improved. However, response of metastatic patients and 5-year survival rates are still poor. In addition, reliable biomarkers for individualized patient selection are still limited [8–10]. Although clinicopathological scores, like the clinical International mRCC Database Consortium model [11], enable stratification of metastatic RCC [1–4] patients irrespective of their subtype, significant differences are observed in clinical outcome among patients within one prognosis group. Molecular scores which might improve risk prediction have been established primarily for the most common subtype ccRCC [12–14], consequently requiring prior accurate subtype determination. However, histopathological diagnosis of RCC is complicated by the introduction of additional histological subtypes with distinct molecular features in the WHO classification (e.g., clear cell papillary RCC) [15–17], and the frequent occurrence of heterogeneous mixed-histological tumors [5], as well as intratumoral heterogeneity [18]. Currently, no molecular signature is available to objectively and accurately identify not only the main subtypes but also their mixtures to improve outcome prediction across subtypes.

Using computational deconvolution and molecular features of the three main histologically defined subtypes of RCC [2, 5] (ccRCC, pRCC, and chRCC), we developed a novel molecular method for continuous subtype classification of RCC. Proposed already two decades ago, the 2016 WHO classification of renal cancer distinguished between pRCC type 1 and type 2 [5]. Notably, based on recent molecular studies suggesting that pRCC type 2 may not constitute a single well-defined entity, pRCC subclassification into type 1 and type 2 is no longer recommended in the updated 2022 WHO classification [17, 19]. Our approach distinguishes pure subtypes from molecularly mixed ones with features from different subtypes, thus enabling the detection of a new class of high-risk tumors with intermediate subtypes. In addition, our novel classification approach into unambiguous and intermediate subtypes opens new avenue for patient stratification and treatment selection for innovative immunotherapies.

## Methods

### Patient cohorts

**Fig. 1:**
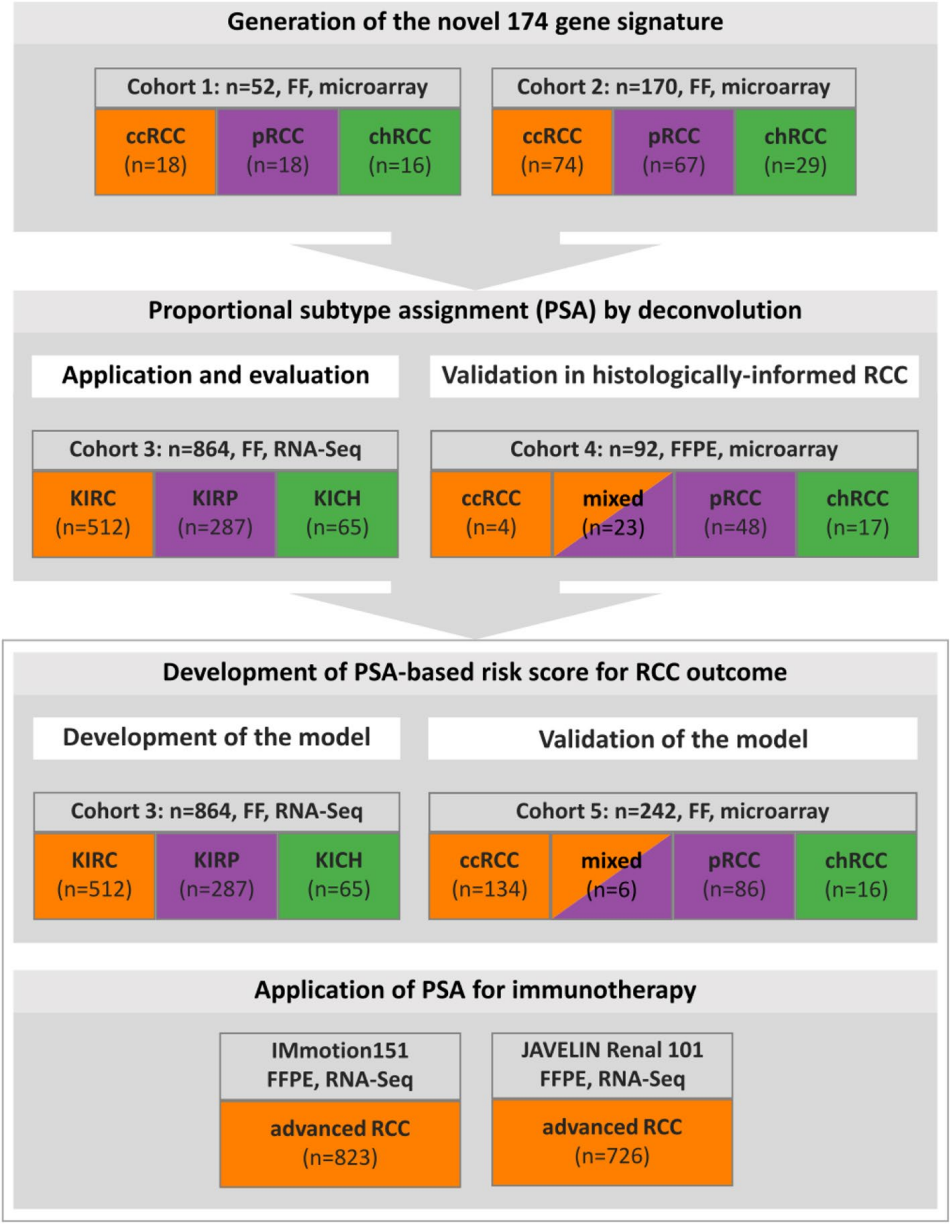
Overview of the general data analysis workflow and the use of the different cohorts. RNA quantification technologies, cohort compositions, and tissue preparation techniques used are given. FF fresh-frozen, FFPE formalin-fixed and paraffin-embedded

The study included five RCC cohorts each comprising cases of ccRCC, pRCC, and chRCC (Fig. 1) for the development and validation of continuous subtype classification and the novel established risk score. Extended information is provided in Additional file 1: Supplementary methods. RCC cohort 1 (C1) included 52 tumors (18 ccRCC, 18 pRCC, 16 chRCC) (Additional file 2: Fig. S1; Additional file 3: Table S1), collected at the Department of Urology, University Hospital Tuebingen, Germany. None of the patients received neoadjuvant therapy before surgery. Tissues were independently evaluated by two teams of pathologists with expertise in renal tumor pathology to assign RCC subtypes.

RCC cohort 2 (C2) (Additional file 4: Table S2) contained a total of 170 RCC samples comprising 158 tumors (74 ccRCC, 55 pRCC, 29 chRCC) from seven studies [20–26] with publicly available transcriptome data from the Genome Expression Omnibus data repository; 11 pRCC collected at the Department of Urology, University Hospital Carl Gustav Carus, Dresden, Germany; and one pRCC collected at the Department of Urology, University Hospital Tuebingen, Germany. C1 and C2 were used to develop the signature (Fig. 1).

RCC cohort C3 (Table 1) consisted of samples from the three renal cancer cohorts of the Cancer Genome Atlas (TCGA) [27–29] including 864 patients (kidney renal clear cell carcinoma, KIRC: 512; kidney renal papillary cell carcinoma, KIRP: 287; kidney chromophobe carcinoma, KICH: 65) with survival data [30] available for 847 patients. Clinical information and gene expression data (“FPKM-UQ”) from TCGA were downloaded on September 25, 2019, from https://gdc.cancer.gov/ using R-package TCGAbiolinks. Disease-specific survival data for the TCGA cohorts was obtained from Liu et al. [30]. Somatic mutation calls generated by the TCGA MC3 project [31] were downloaded from https://gdc.cancer.gov/about-data/publications/mc3-2017 (mc3.v0.2.8.PUBLIC.maf.gz). A pathological re-evaluation of the TCGA RCC cohort was obtained from Ricketts et al. [32]. Patients receiving prior treatment related to their disease were excluded. In cohort C3, proportional subtype assignment (PSA) was evaluated and its association to survival was analyzed (Fig. 1).

**Table 1:** Characteristics of the discovery and the validation cohorts

	**C3 (discovery, *n* = 864)**		**C5 (validation, *n* = 242)**	
**Characteristic**	***n***	**%**	***n***	**%**
**Sex**				
Male	579	67.0	165	68.2
Female	285	33.0	77	31.8
**T**				
1	476	55.1	124	51.2
2	122	14.1	21	8.7
3	251	29.1	92	38
4	13	1.5	4	1.7
NA	2	0.2	1	0.4
**N**				
0	322	37.3	173	71.5
1/2	46	5.3	26	10.7
X	495	57.3	40	16.5
NA	1	0.1	3	1.2
**M**				
0	537	62.2	170	70.2
1	82	9.5	38	15.7
X	209	24.2	33	13.6
NA	36	4.2	1	0.4
**Histology**				
ccRCC	512	59.3	134	55.4
pRCC	287	33.2	86	35.5
chRCC	65	7.5	16	6.6
mixed	0	0.0	6	2.5
**Overall survival**				
Alive	648	75.0	161	66.5
Deceased	216	25.0	81	33.5
**CSS**				
Censored	714	82.6	188	77.7
Events	135	15.6	54	22.3
NA	15	1.7	0	0.0
**Follow-up, years**				
Median	3.0		4.8	
Range	0 to 16.2		0 to 21.2	
NA	2	0.2	0	0.0
**Age, years**				
Median	60		64	
Range	17 to 90		25 to 90	
NA	3	0.3	0	0.0
**Tumor size, cm**				
Median	5.1		5.8	
Range	1 to 25		1.3 to 17.7	
NA	105	12.2	2	0.8

*NA* Not available, *CSS* Cancer-specific survival

Cohort 4 (C4) included 92 independent cases (4 ccRCC, 48 pRCC, 17 chRCC, 23 tumors with mixed histology) and was used to evaluate our method with expression data generated from FPPE tissues (Fig. 1). Seventeen chRCC and 23 tumors with mixed histology were obtained from the Institute of Pathology, University Hospital, Friedrich-Alexander-University Erlangen-Nürnberg (FAU), Erlangen, Germany; 26 pRCC derived from the study by Polifka et al. [33] were collected from several participating centers in Germany; and 15 pRCC were obtained from the Department of Pathology, Medical University of Vienna, Vienna, Austria. Four ccRCC and seven pRCC were collected by the Department of Urology, University Hospital Tuebingen, Germany.

Cohort 5 (C5) (Table 1) comprises 242 independent RCC samples (134 ccRCC, 86 pRCC, 16 chRCC, 6 tumors with mixed histology) that were consecutively collected from the Department of Urology, University Hospital Tuebingen, Germany (*n* = 161); from the Department of Urology, University Hospital Carl Gustav Carus, Dresden, Germany (*n* = 44); from the Department of Pathology, Portuguese Oncology Institute of Porto (IPO Porto), Portugal (*n* = 27); and from the Department of Urology, University of Greifswald, Germany (*n* = 10). C5 was used to validate results from the survival analysis (Fig. 1).

Additionally, clinical and RNA-seq data from the JAVELIN Renal 101 trial (*n* = 726) [9, 34] and the IMmotion151 trial (*n* = 823) [35, 36] were used to study the association of the molecular classification with progression-free survival (PFS). In brief, JAVELIN Renal 101 (NCT02684006) is a worldwide multicenter, randomized, open-label, phase 3 trial comparing checkpoint inhibition (avelumab) plus the tyrosine-kinase inhibitor axitinib with monotherapy of the tyrosine-kinase inhibitor sunitinib. The age of eligible patients was ≥ 18 years and patients with untreated advanced RCC with a clear-cell component were included. Normalized gene expression data (TPM) generated by RNA-Seq from FFPE tissue as well as clinical information were available for 726 patients [9]. The IMmotion151 trial (NCT02420821) is a worldwide multicenter, open-label, phase 3, randomized controlled trial comparing checkpoint inhibition (atezolizumab) plus VEGF inhibition (bevacizumab) with monotherapy of the tyrosine-kinase inhibitor sunitinib. Eligible patients were aged ≥ 18 years with unresectable locally advanced or metastatic RCC with any component of clear cell or sarcomatoid histology. Normalized gene expression data (TPM) generated by RNA-Seq from FFPE tissue and clinical data of 823 participants were obtained from the European Genome-phenome Archive (EGA) (accession number: EGAS00001004353).

### RCC-derived cell lines

Transcriptomic data as provided by the Broad-Novartis Cancer Cell Line Encyclopedia (CCLE) [37, 38] as well as the COSMIC Cell Lines Project (CCLP) [39, 40] were used from 14 RCC-derived cell lines (i.e., 769-P, 786-O, A498, A704, ACHN, BFTC-909, CAKI-1, CAL-54, KMRC-1, KMRC-20, OS-RC-2, RCC10RGB, UO31, VMRC-RCZ). For detailed information, see Additional file 5: Table S3.

### Gene expression analyses

Total RNA was isolated from fresh-frozen tissue of cohorts C1, C2, and C5 as previously described [41, 42]. RNA from FFPE tissue of cohort C4 was isolated using the AllPrep DNA/RNA FFPE Kit (Qiagen, Germany). Genome-wide transcriptome analyses were performed using GeneChip™ Human Transcriptome Array 2.0 (Thermo Fisher Scientific). Processing of microarray data was performed as described [42]. The data accession number at the European Genome-phenome Archive (EGA) (www.ebi.ac.uk/ega/home), which is hosted by the EBI and the CRG, is EGAS00001001176. Processing of publicly available transcriptome data of all other cohorts is described in supplementary data.

### Statistical analysis

#### Statistical tools

Detailed information of all statistical and bioinformatics methods is given in Additional file 1: Supplementary methods.

All statistical analyses were performed with R-3.6.1 [43] including additional packages beanplot_1.2 [44], coin_1.3–1 [45], MASS_7.3–51.4 [46], partykit_1.2–5 [47, 48], Rfast_2.0.1 [49], rms_5.1–3.1 [50], squash_1.0.8 [51], survival_2.44–1.1 [52], and twosamples_1.0.0 [53] from CRAN (http://cran.r-project.org). GEOquery_2.46.15 [54], limma_3.40.6 [55], oligo_1.48.0 [56], org.Hs.eg.db_3.8.2 [57], pda.hta.2.0_3.12.2 [58], Rgraphviz_2.30.0 [59], SCAN.UPC_2.26.0 [60], SummarizedExperiment_1.14.1 [61], and TCGAbiolinks_2.12.6 [62] are part of the Bioconductor software project (http://www.bioconductor.org, version 9). For Affymetrix microarrays, customized CDF files provided by brainarray [63, 64] (version 23) were used.

For gene expression deconvolution, expression levels were required to be in linear space. Hence, log2 expression levels from microarray analysis were exponentiated. Raw counts from RNA-Seq measurement had to be normalized for sequencing depth and gene length to allow for intrasample analysis. Preceding deconvolution linear expression values were mean-centered and standardized.

For the principal component analysis, FPKM-UQ and TPM expression values were log2-transformed (log2(*x* + 1)).

Survival analyses for endpoints cancer-specific survival (CSS) and PFS were conducted by Kaplan–Meier curves and corresponding log-rank tests as well as uni- and multivariate Cox models. Comparisons of Cox models were performed by analysis of deviance.

All statistical tests were two-sided. Statistical significance was defined as *P*-value < 0.05. Where indicated, *P*-values were corrected for multiple testing applying Holm’s [65] method.

The Akaike information criterion (AIC) was used for model selection. It is an estimator of the relative amount of information lost by a given model, measuring the trade-off between model fit and model complexity. The preferred model is the one with minimum AIC value.

#### Generation of the gene signature

Subtype-specific genes were determined using gene expression data from C1 as described in Additional file 1: Supplementary Methods resulting in 1379 ccRCC-, 844 pRCC-, and 1463 chRCC-specific genes (total 3686) (Additional file 2: Fig. S2). Out of these genes, signature genes were selected by evaluating various signature gene matrices (Additional file 2: Fig. S3). Starting with two genes per subtype that exhibited the highest fold change relative to each of the other two subtypes, matrices with increasing numbers of subtype-specific genes were iteratively created and used to deconvolve the 170 samples of cohort C2. Matrices consisted of median linear expression values per RCC subtype based on C1. Based on the assumption that the accuracy of deconvolution increases with the number of genes included, the largest matrix was chosen that produced a substantial change in subtype deconvolution compared to its predecessor matrix. The final signature matrix included 174 genes, i.e., 58 genes per subtype (see Additional file 1: Supplementary Methods; Additional file 2: Fig. S3).

#### Proportional subtype assignment (PSA)

Samples from RCC tissue were considered as composite samples that may combine specific molecular features from ccRCC, pRCC, and chRCC. The proportional subtype assignment (PSA) was determined using gene expression deconvolution. In brief, the expression of the 174 signature genes in an RCC sample of interest was modeled as the weighted sum of expression of these genes in ccRCC, pRCC, and chRCC. Based on the weights identified by robust linear regression, the proportional composition of the sample was then calculated such that c + p + h = 100%, where c, p, and h represent the ccRCC, pRCC, and chRCC proportions, respectively (details see Additional file 1: Supplementary methods).

#### Development of the RCC-R score

Our approach estimates three percentage values per sample, representing the predicted proportions of ccRCC, pRCC, and chRCC. Subtype proportions termed also as scores were modeled with flexible restricted cubic spline (RCS) functions as well as with cubic polynomials in Cox proportional hazard regression. Linear predictors from Cox proportional hazard models were used as prognostic index (PI). The predictive accuracy of single subtype scores as well as their combination was compared by repeated tenfold cross-validation in cohort C3. For comparison, the pathological classification was evaluated as categorical predictor of survival. The ccRCC-score modeled via cubic polynomials, hereafter termed RCC-R score, showed the best trade-off between model complexity and predictive accuracy and consequently was selected as a biomarker for risk prediction (details see Additional file 1: Supplementary Methods). With PSA specified on 0–1 scale, the prognostic index (PI) for a RCC sample with a ccRCC proportion (RCC-R score) of ***c*** was determined as follows:

1 $${\varvec{P}}{\varvec{I}}={\varvec{c}} \times 14.71-{{\varvec{c}}}^{2}\times 25.46+ {{\varvec{c}}}^{3}\times 12.21-1.46$$

#### Survival or therapy outcome analyses

CSS was used as an endpoint of survival analyses in cohorts C3 and C5. CSS was defined as time from surgery to death or last date of follow-up if alive. Data for patients who died from other causes than RCC were censored at the time of death. PFS, as defined in the JAVELIN Renal 101 and IMmotion151 trials [9, 35], was used as endpoint of survival analyses in the treatment trials.

## Results

### Development of a 174-gene signature matrix for deconvolution and molecular subtype classification

Based on candidate genes selected for subtype classification (Fig. 1), a final gene signature of 174 genes (the top 58 subtype-specific genes per ccRCC, pRCC, and chRCC) (Additional file 2: Fig. S3B; Additional file 6: Table S4) was developed using cohorts C1 and C2 as outlined in supplementary methods (Additional file 1: Supplementary methods; Additional file 2: Fig. S2/S3).

**Fig. 2:**
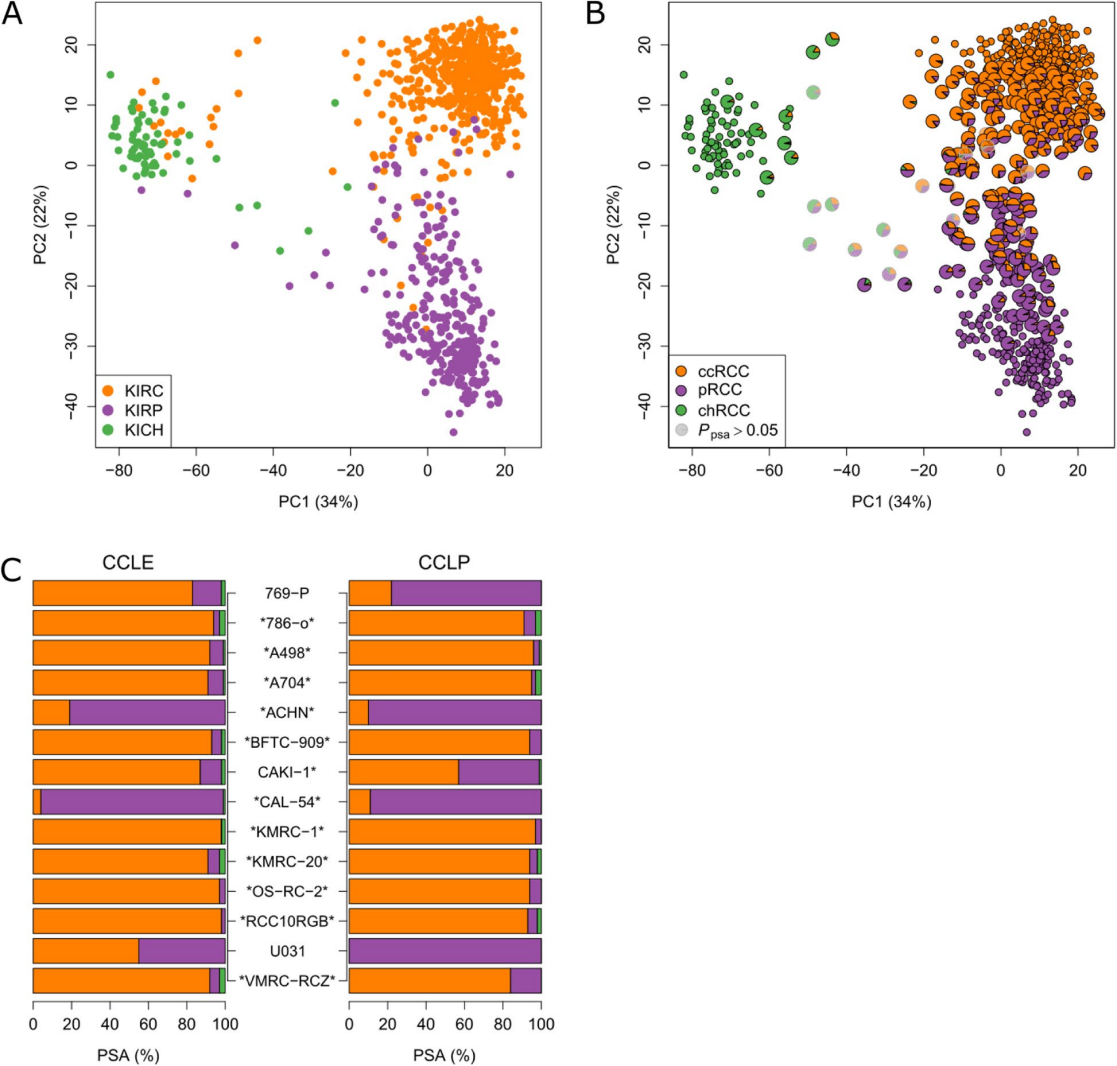
Proportional subtype assignment (PSA) of RCC and RCC cell lines. **A**, **B** Principal component analysis of the TCGA RCC cohort (C3) using expression data of the 174 signature genes. **A** TCGA cohorts of ccRCC (KIRC, *n* = 512), pRCC (KIRP, *n* = 287), and chRCC (KICH, *n* = 65) are displayed. **B** PSA were determined for tumors of C3 by computational deconvolution. A total of 246 RCC samples with maximum PSA values below 95% were considered as potential heterogeneous tumors (enlarged symbols). Their molecular subtype composition based on PSA is visualized by pie charts. Nineteen samples with ${P}_{psa}>0.05$ as determined by a permutation *P*-value approach are displayed by shaded pie charts with gray borders. **C** PSA for 14 RCC-derived cell lines were calculated using transcriptomic data as provided by the Broad-Novartis Cancer Cell Line Encyclopedia (CCLE) [37, 38] as well as the COSMIC Cell Lines Project (CCLP) [39, 40]. Asterisk indicates ${P}_{psa}<0.05$

Using these signature genes, a principal component analysis (PCA) of the TCGA RCC cohort (C3) (Table 1) including RNA-seq data of 864 tumors was carried out (Fig. 2A). Principal component 1 discriminated tumors originating from distal cell types (KICH) from those arising from the proximal tubule (KIRC and KIRP). The incorrect classifications, particularly of some chRCC samples (Fig. 2A), have been reported [12, 32] and were also observed in this analysis. Interestingly, KIRC and KIRP cohorts were not fully separated from each other. Additionally, PCA was performed using the 174 signature genes, but including both 864 tumors and 128 samples of adjacent non-tumor tissue from the TCGA RCC cohort (Additional file 2: Fig. S4A). Here, non-tumor samples formed a cluster, which was separated from tumors by principal component 1.

### Molecular tumor characterization by proportional subtype assignment

Next, rather than categorizing a tumor, we intended to model its molecular characteristics through proportional subtype assignment (PSA). Therefore, transcriptomes of 864 RCC from RCC cohort C3 were deconvolved and PSA for each sample were computed (Additional file 7. Table S5). Following significance filtering (${P}_{psa}<0.05$), results of PSA were analyzed for 845 samples (97.8%). Using an arbitrary threshold of 95% for PSA to distinguish between tumors with a unique subtype assignment and cases with overlapping features according to PSA revealed 246 (29%) potential heterogeneous tumors (Fig. 2B) mainly with clear cell and papillary characteristics.

Non-tumor samples consistently exhibited heterogeneous PSA (Additional file 2: Fig. S4B/C). However, they were assigned combinations of subtype proportions that were exceptional in tumors. Only seven of 864 tumor samples of which six had a non-significant PSA (${P}_{psa}>0.05$), lie within the range of PSA of non-tumor samples (Additional file 2: Fig. S4C/D).

In addition to bulk tumors from TCGA, application of PSA to RCC cell lines from the Cancer Cell Line Encyclopedia (CCLE) and the COSMIC Cell Lines Project (CCLP) confirmed unambiguously ACHN and CAL54 as cell lines with pRCC characteristics, whereas all other cell lines are classified as predominantly ccRCC (Fig. 2C, Additional file 5: Table S3 and Additional file 2: Fig. S5), which is in line with accepted classifications of these cell lines [66].

**Fig. 3:**
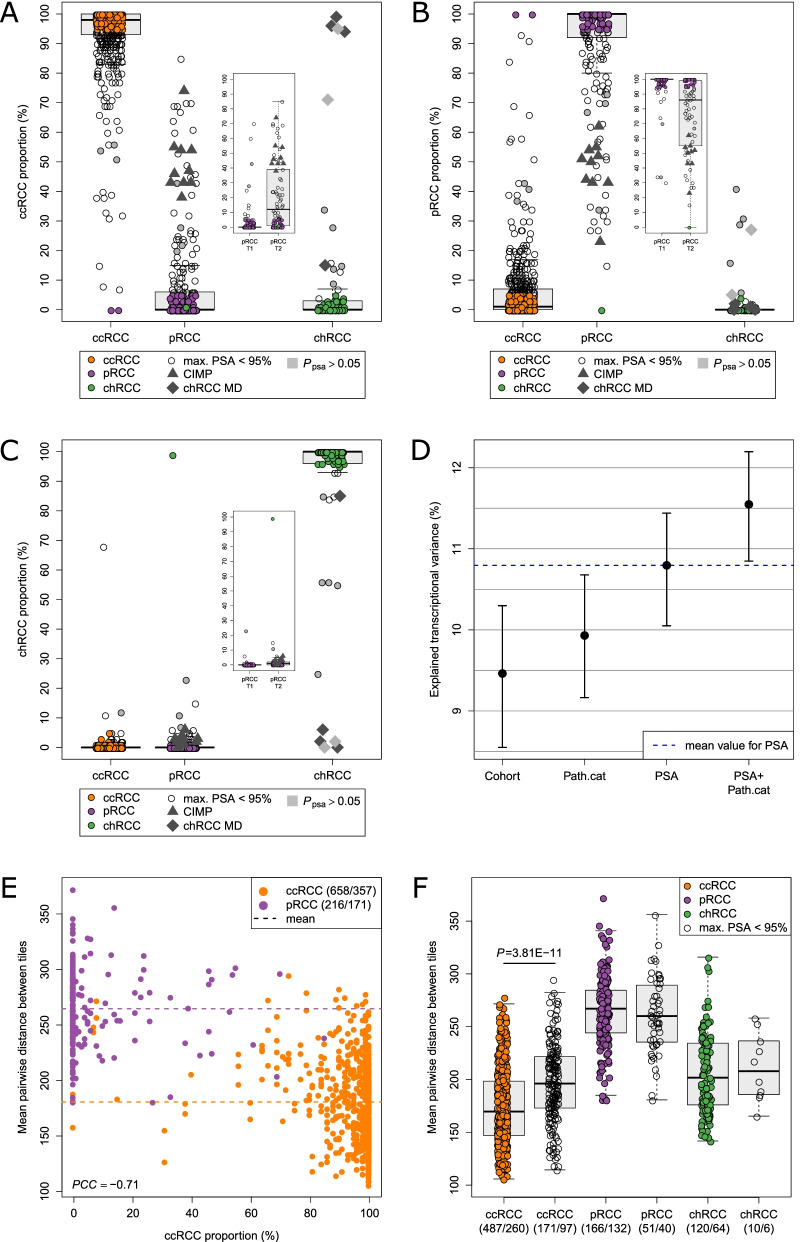
Distribution of PSA in histologically defined RCC subtypes. **A**–**C** Distributions of assigned proportions of ccRCC (**A**), pRCC (**B**), and chRCC (**C**) to tumors of C3 are shown for distinct, pathologically defined subgroups including 469 ccRCC, 270 pRCC including 159 pRCC T1 and 78 pRCC T2, and 80 chRCC, respectively. Tumors with a maximum PSA value ≥ 95% are colored. Furthermore, ten tumors with the CpG island methylator phenotype (CIMP), a molecular pRCC subtype with a specific methylation profile, are indicated, as well as six metabolically divergent (MD) chRCC. Samples with ${P}_{psa}>0.05$ are marked in gray. Boxes refer to median and interquartile ranges with whiskers extending to a maximum of 1.5 times the interquartile range. **D** The information content of different subtype classifications was quantified by determining the amount of variance they explained in gene expression data. Expression of 25,208 genes in 623 tumors consisting of 466 ccRCC, 116 pRCC, and 41 chRCC that were repeatedly resampled (with replacement) from 805 cases of C3 was analyzed. Points and error bars indicate the mean together with the 95% value range of the resampling distribution. “Cohort” refers to the TCGA cohorts KIRC (*n* = 479), KIRP (*n* = 266), and KICH (*n* = 60). “Path.cat” comprises ccRCC (*n* = 466), pRCC (*n* = 266), and chRCC (*n* = 73) subgroups. Additionally, the combination of Path.cat and PSA was evaluated. Samples with ${P}_{psa}>0.05$ were not considered here. **E**, **F** Relationship between PSA and computational histopathology. The mean pairwise Manhattan distance between 50 randomly selected tiles per tumor tissue slide was used as a measure of histopathological complexity. A circle represents one tissue slide, and multiple slides may be present per tumor. The values in parentheses indicate the number of the slides and associated tumors. Samples with ${P}_{psa}>0.05$ were not considered here. **E** Histological complexity is displayed in dependence on the ccRCC proportion. Slides from samples classified as either chRCC or with chRCC proportion above 5% were excluded. Colors indicate the pathological classification, and the dashed lines display the mean distance of the respective set of slides. The Pearson correlation coefficient (PCC) was calculated. **F** Per pathologically defined RCC subgroup (Path.cat), histopathological complexity was compared between tumors with maximum PSA value ≥ 95% (filled circles) and potential heterogeneous tumors with maximum PSA value < 95% (open circles) using the *t*-test. Boxes refer to median and interquartile ranges with whiskers extending to a maximum of 1.5 times the interquartile range

Next, PSA and standard pathological categorization were compared using a recently published pathological re-evaluation of the TCGA RCC cohort [32] (Fig. 3A–C). The tumors categorized as heterogeneous based on RNA profiles consisted almost exclusively of ccRCC and pRCC histological subtypes and tended to be assigned to pathological T3 and T4 (*P* = 0.0024, Fisher test). Among pRCC, tumors with mixed features were mostly of subtype 2, according to the previous WHO 2016 classification of renal cancer [5]. Notably, tumors characterized by a CpG island methylator phenotype (CIMP), a molecular pRCC subtype with a specific methylation profile [28], were among the pRCC with high ccRCC content. Additionally, chRCC tumors with molecular features of ccRCC include recently identified metabolically divergent chRCC with sarcomatoid features [32].

In three cases, PSA and pathological classification differed significantly (Fig. 3A–C). The genomic subtype classification of RCC introduced by Chen et al. [67] confirmed our classification by PSA for these cases (TCGA-A3-3363, TCGA-B0-5707, and TCGA-BQ-7055) (Additional file 7: Table S5).

Next, the amount of explained transcriptional variance in simulated populations of RCC based on cohort C3 was used as a measure to compare the information content in PSA and pathological classification (Fig. 3D). During re-evaluation of the TCGA RCC cohort [32], 13 KIRC cases were classified as chRCC, which increased information content of pathological classification (Path.cat). Determining the proportions of the three main subtypes in individual tumors through PSA generated significantly more information than categorical assignment to one of these subtypes. The highest information content could be generated by combining pathology and PSA.

The relationship between PSA and the occurrence of somatic mutations in candidate genes known to be affected only in certain subtypes [67] (Additional file 2: Fig. S6) confirmed reliability of subtype prediction by PSA.

To further investigate the histopathological characteristics of heterogeneous tumors, we analyzed distances within and between tumors based on histopathological features recently extracted from TCGA whole-slide images using computer vision [68]. First, the mean of the pairwise distances between tiles of the same slide was tested as a measure of histopathological complexity of the scanned tissue. Comparison between slides of the same tumor as well as of different tumors showed that this measure was independent of the slide and could discriminate between tumors (Additional file 2: Fig. S7). A correlation between histopathological complexity and the transition from pRCC to ccRCC was observed (Fig. 3E). In particular, ccRCC with papillary portions showed a higher complexity than unambiguously assigned ccRCC cases (*P* = 3.8E − 11, *t*-test) (Fig. 3F).

### PSA using formalin-fixed paraffin-embedded tissue and comparison against histopathology

**Fig. 4:**
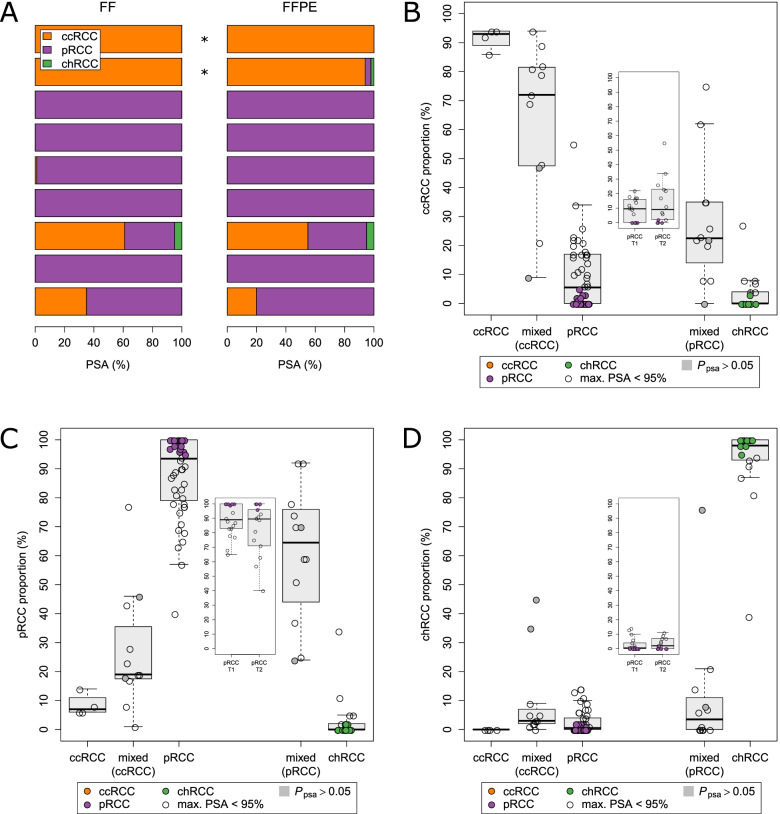
Subtype prediction through PSA in formalin-fixed and paraffin-embedded (FFPE) tissue. **A** PSA based on fresh-frozen (FF) samples from 9 RCC were compared to PSA based on matching FFPE samples. Whole-transcriptome profiles generated by RNA-Seq for two ccRCC were obtained from Li et al. (marked by asterisks) [82]. The remaining 7 tumors (pRCC) from the present study have been analyzed using microarray technology. For all 18 samples, ${P}_{psa}$ was below 0.05. **B**–**D** PSA were determined for 92 FPPE tissues of cohort C4. Gene expression was quantified using microarray technology. The assigned proportions of molecular features of ccRCC (**B**), pRCC (**C**), and chRCC (**D**) were compared with the original pathological classification. According to pathology, 23 tumors had a mixed histology dominated either by clear cell morphology (*n* = 11) or by papillary features (*n* = 12). Additionally, 4 ccRCC, 48 pRCC including 18 pRCC T1 and 14 pRCC T2, and 17 chRCC were analyzed. Tumors with PSA values ≥ 95% are colored. Samples with ${P}_{psa}>0.05$ are marked in gray. Boxes refer to median and interquartile ranges with whiskers extending to a maximum of 1.5 times the interquartile range

Cohorts C1, C2, and C3 included only fresh-frozen tissue. Therefore, we assessed the applicability of PSA for gene expression data derived from FFPE tissue. As shown in Fig. 4A for 9 independent tumors, comparable results were found for PSA in matched FFPE and fresh-frozen samples. Additionally, we investigated 92 FPPE tissues (cohort C4), which have been independently evaluated by experts for renal tumor pathology comprising 4 ccRCC, 48 pRCC, 17 chRCC, and 23 tumors with a mixed-type histology dominated either by clear cell morphology (*n* = 11) or by papillary features (*n* = 12) to demonstrate the reliability of PSA particularly for histologically challenging cases (Fig. 4B–D). PSA was in very good agreement with original histopathological diagnosis for ccRCC and chRCC. Histologically defined pRCC cases showed variable, but predominant proportions of pRCC in PSA, with a median pRCC proportion of 93.5%. A higher variability of pRCC proportion in PSA for previously defined type 2 cases was observed, but the difference in the median pRCC proportion between type 1 (*n* = 18) and type 2 (*n* = 14) was not significant. In addition, PSA confirmed the original histopathological diagnosis of 23 mixed-type RCC with assigned proportions of molecular features of ccRCC and pRCC, confirming the prevalence of either clear cell or papillary features.

### Identification of intermediate subtypes with poor prognosis

As RCC subtypes are known to vary in prognosis [32], we investigated the association of calculated molecular subtype proportions, synonymously referred to as subtype scores, to patient survival. Log relative hazards differentiating the individual risk of patients depending on subtype scores were used as prognostic index (PI). Modeling subtype scores with flexible restricted cubic spline (RCS) functions revealed a significant association to CSS, particularly, in case of the ccRCC- (*P* = 4.1E − 10, log-rank test) and the pRCC-score (*P* = 6.5E − 10, log-rank test) (Additional file 2: Fig. S8B).

**Fig. 5:**
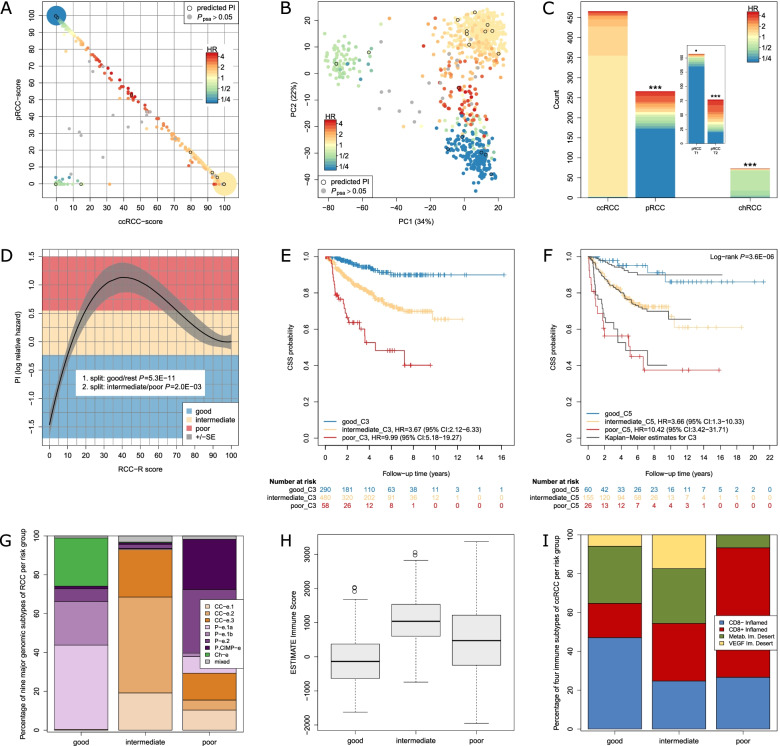
Risk prediction for RCC based on PSA and the RCC-R score. **A**–**C** PSA were used as predictors of survival in C3 (*n* = 864). A prognostic index (PI), which differentiated the individual risk of patients based on ccRCC- and pRCC-score, was calculated as detailed in the “Methods” section. For samples without available survival data, the PI was predicted. Samples with ${P}_{psa}>0.05$ are marked in gray. Hazard ratios (HR) were obtained by exponentiating the PI. **A** Combinations of ccRCC- and pRCC-score values are colored according to their HR. Points in the corners represent 201 (bottom right), 165 (top left), and 48 (bottom left) cases, respectively. **B** Principal component analysis plot is shown with samples colored according to their HR. **C** Distributions of HR in distinct, pathologically defined subtypes (*n* = 805) are displayed. Samples with ${P}_{psa}>0.05$ are not shown here. Per histological subtype, a Cox regression of cancer-specific survival (CSS) on the respective subset of PI was conducted. Log-rank *P*-values are indicated by the level of significance: “***” *P* < 0.001, “**” *P* < 0.01, “*” *P* < 0.05, “.” *P* < “0.1”. **D**–**F** Relationship of cancer-specific survival (CSS) and the ccRCC-score, termed as RCC-R score, is shown. **D** The curve displays the estimated relationship as specified in Eq. 1 in the “Methods” section between the RCC-R score, modeled via cubic polynomials, and the PI in C3 (*n* = 828). Using conditional inference trees with endpoint CSS, the PI was categorized into three risk groups (good (*n* = 290), intermediate (*n* = 480), and poor (*n* = 58)). Corresponding *P*-values from recursive binary splitting are indicated. **E** Kaplan–Meier curves of CSS for risk groups based on the RCC-R score are shown for the discovery cohort (C3). Additionally, HR with the good group as reference are specified for the intermediate and the poor group. **F** Kaplan–Meier curves of CSS for risk groups based on the RCC-R score in the validation cohort (C5, *n* = 241) are shown by colored curves. Corresponding Kaplan–Meier curves for C3 are added for comparison. Indicated HR and log-rank test *P*-value result from Cox regression analysis in C5. **G**–**I** Relationship of RCC-R score with established molecular signatures. Risk groups derived from the RCC-R score in cohort C3 were compared with different molecular-based classifications or signatures available for the combined TCGA RCC or the KIRC cohort. **G** Bar chart showing distribution (%) of nine major genomic subtypes of RCC (as established by multi-omics analysis [67]) per risk group (good (*n* = 290), intermediate (*n* = 480), and poor (*n* = 58)). **H** Boxplots showing immune infiltration as predicted by the ESTIMATE method [70] per risk group (good (*n* = 290), intermediate (*n* = 480), and poor (*n* = 58)). **I** Bar chart showing distribution (%) of four immune subtypes of ccRCC [71] per risk group (good (*n* = 17), intermediate (*n* = 421), and poor (*n* = 15))

Next, ccRCC- and pRCC-score were used in combination (which implied the chRCC-score). In Fig. 5A–C, samples were colored according to their hazard ratio (HR). The highest risk for cancer-specific death in cohort C3 was found for molecularly heterogeneous tumors displaying overlapping ccRCC and pRCC characteristics (Fig. 5A). These tumors are located between the main clusters of ccRCC and pRCC (Fig. 5B). The lowest risk was assigned to few scattered chRCC and a large subset of pRCC (Fig. 5B). In particular, the variability in risk within pRCC type 2 could be captured by risk prediction based on PSA (Fig. 5C).

Using both PSA and pathological categories in Cox modeling revealed that information on histopathologically defined subtypes did not contribute significantly beyond PSA (*P* = 0.85, chi-square test) (Additional file 2: Fig. S9A).

### Development of a risk prediction model for RCC based on PSA

The observed strong link to CSS enabled the development of a PSA-based risk score using the ccRCC, pRCC, and chRCC proportions. Because the combination of ccRCC- and pRCC-score covers the PSA information completely, the chRCC score has not been further considered (Additional file 2: Fig. S8). Subsequently, the predictive ability of the individual ccRCC- and pRCC-score as well as their combination was evaluated. Tumors with proportions of both ccRCC and pRCC had the highest risk, indicating a non-linear relationship between these subtype scores and the log relative hazard (Additional file 2: Fig. S10). The ccRCC-score modeled via cubic polynomials, hereafter termed the RCC-R score, showed the best trade-off between prediction accuracy and model complexity in a repeated tenfold cross-validation analysis testing different modeling approaches (supplementary methods, Additional file 2: Fig. S11A). Survival prediction by Cox modeling based on the novel established RCC-R score compared to the pathological categories (Path.cat) was significantly improved (*P* = 3.6E − 11, chi-square test, Additional file 2: Fig. S9B). Histopathology did not provide significant independent prognosis-relevant information (*P* = 0.059, chi-square test, Additional file 2: Fig. S9B). C-indices for the Path.cat and the RCC-R score, when used individually as predictors, were 0.56 and 0.67, respectively.

Computation of the prognostic index (PI) based on the RCC-R score is detailed in the “Methods” section. Predicted 1-, 2-, and 5-year CSS probabilities in dependence on the RCC-R score are shown in Additional file 2: Fig. S11B. Furthermore, conditional inference trees applied to the PI identified three risk groups, including 290 patients with good, 480 patients with intermediate (HR = 3.7, 95% CI: 2.1–6.3) and 58 patients with poor clinical outcome (HR = 10, 95% CI: 5.2–19.3), respectively (Fig. 5D, E; Additional file 2: Fig. S11C). Both the good and the poor groups combined histologically different tumors, with the good group encompassing 97% of chRCC, 97% of pRCC type 1, 49% of pRCC type 2, and 75% of unclassified pRCC. The poor group mainly consisted of pRCC type 2 (56%), with molecular overlapping ccRCC features, and ccRCC (32%), whereas the intermediate group was nearly exclusively populated by ccRCC (95%) (Additional file 2: Fig. S11D).

### Validation of the RCC-R score

The RCC-R score-based prediction of CSS was validated in an independent cohort (C5) including 134 ccRCC, 86 pRCC, 16 chRCC, and 6 cases with known mixed subtypes (Table 1, Additional file 2: Fig. S12A). In contrast to C3 (RNA-Seq), gene expression in C5 was quantified using microarray technology. Transcriptomes were deconvolved and the PI for C5 (PI_C5_) was calculated. By means of the cutoffs learned in C3, 241 cases with ${P}_{psa}<0.05$ of C5 were divided into 60 cases with good, 155 with intermediate (HR = 3.66, 95% CI: 1.3–10.33), and 26 with poor clinical outcome (HR = 10.42, 95% CI: 3.42–31.71) (Fig. 5F, Additional file 2: S12B). Notably, the Kaplan–Meier curves for these groups were consistent with their equivalents from the derivation cohort C3 (Fig. 5F) as well as with the predicted survival probabilities for the three risk groups based on baseline survival function and PI_C5_ (Additional file 2: Fig. S12C). Univariate Cox regression analysis revealed that the continuous PI_C5_ was significantly associated to CSS (*P* = 3.2E − 05; HR = 3.02, 95% CI: 1.8–5.08). Hence, calibration and discrimination of the RCC-R score model were similar in C5, indicating successful independent validation [69]. Finally, even when used in a multivariate model together with clinicopathological parameters stage (T), nodal status (N), and metastasis (M), as well as histology (Table 2), the contribution of PI_C5_ remained significant.

**Table 2:** Multivariate Cox analyses of cancer-specific survival in 237 patients of the validation cohort (C5) with ${P}_{psa}<0.05$ and all clinicopathological parameters available

Variable	Level	Hazard ratio (95% CI)	*P*-value
**Sex**			
	Female	1	
	Male	0.88 (0.46–1.70)	0.71
**Age, years**			
	Linear	0.99 (0.97–1.02)	0.59
**T**			
	1	1	
	2	1.83 (0.4–8.43)	0.44
	3	5.12 (2.1–12.51)	3.4E − 04
	4	11.49 (2.54–51.88)	1.5E − 03
**N**			
	0	1	
	1/2	1.62 (0.73–3.62)	0.24
	X	0.53 (0.14–1.96)	0.34
**M**			
	0	1	
	1	6.06 (2.74–13.41)	8.7E − 06
	X	1.45 (0.32–6.51)	0.63
**Tumor size, cm**			
	Linear	0.92 (0.83–1.02)	9.7E − 02
**Histology**			
	ccRCC	1	
	pRCC	2 (0.96–4.16)	6.2E − 02
	chRCC	8.8 (0.86–89.78)	6.7E − 02
	Mixed	1.88 (0.28–12.67)	0.52
**PI**_**5**_** (RCC-R score)**			
	Linear	2.14 (1.14–4.04)	1.8E − 02

In an approach for better molecular understanding of the identified risk groups, they were compared to recent classifications and cluster analyses, which identified multilevel genomic and immune RCC subtypes [67, 70, 71], using TCGA cohorts (Fig. 5G–I). Although the CIMP cluster is enriched in the poor group, there is no complete overlap with the nine major genomic subtype categories defined by Chen et al. [67]. Additionally, the immune score [70] is not significantly different between outcome groups. Further investigation of immune subtypes defined by Clark et al. [71] showed a trend towards higher frequency of CD8 inflamed tumors in the poor outcome group, but CD8 inflamed tumors are also present in the intermediate and even good prognostic group.

### Association of PSA with progression-free survival in the JAVELIN Renal 101 and the IMmotion151 trials

**Fig. 6:**
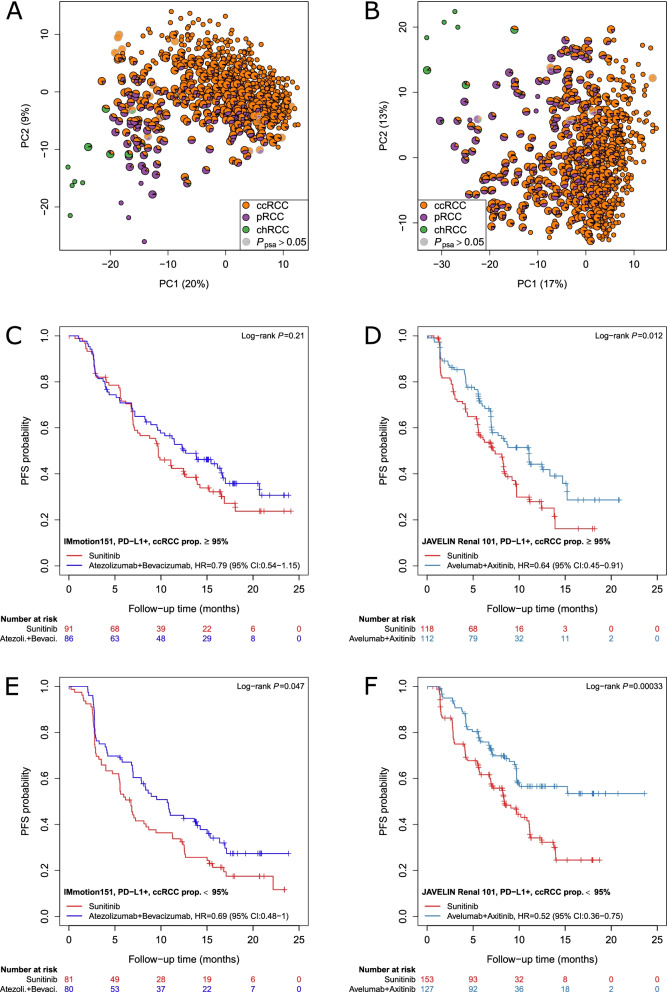
Prediction of therapeutic outcome by PSA in the IMmotion151 and JAVELIN Renal 101 trials. **A**, **B** Principal component analysis (PCA) using expression of 174 signature genes identified 341 of 823 samples and 407 of 726 samples as heterogeneous tumors in the IMmotion151 and of the JAVELIN Renal 101 trials, respectively. Here, tumors with maximum PSA value below 95% were considered as potential heterogeneous. Their molecular subtype is visualized by pie charts (enlarged symbols). Samples with non-significant PSA (${P}_{psa}>0.05$) are displayed by shaded pie charts with gray borders (IMmotion151 *n* = 23; JAVELIN Renal 101 *n* = 12). **C**–**F** Kaplan–Meier curves of progression-free survival (PFS) are shown for PD-L1-positive tumors in both cohorts with a ccRCC proportion of at least (**C**, **D**) or less (**E**, **F**) than 95% based on PSA. Cox regression analysis was used to determine *P*-values (log-rank test) and HR of checkpoint inhibition with tyrosine-kinase inhibition versus sunitinib, respectively

First, we investigated the tumors of patients in the JAVELIN Renal 101 (*n* = 726) and the IMmotion151 trials (*n* = 823) through PSA using public available RNA-seq data. Molecularly heterogeneous tumors with ccRCC and pRCC features were uncovered in both cohorts (Fig. 6A, B). Next, in both cohorts, the subsets of PD-L1-positive tumors (Additional file 2: Fig. S13) were stratified into molecularly heterogeneous and unambiguous cases depending on the assigned ccRCC proportion, using 95% as a cutoff. Interestingly, molecularly heterogeneous PD-L1-positive tumors showed higher response rates to checkpoint inhibition in combination with a tyrosine-kinase inhibitor (axitinib) or antibody (bevacizumab) compared to sunitinib monotherapy, both in the JAVELIN Renal 101 (*P* = 3.3E − 04; HR = 0.52, 95% CI: 0.36 − 0.75) and in the IMmotion151 trial (*P* = 0.047; HR = 0.69, 95% CI: 0.48–1) (Fig. 6C–F). In addition, distinguishing between heterogeneous and unambiguous subtypes based on PSA significantly improved prediction of PFS in PD-L1-positive tumors in the JAVELIN Renal 101 (*P* = 0.013) and the IMmotion151 trials (*P* = 0.032).

## Discussion

RCC is a heterogeneous disease thereby complicating reliable subtype identification based on histopathology alone. Subtype identification is crucial for treatment-related outcome prediction. Selection of therapeutic strategies including immunotherapy might be improved by incorporating molecular data as previously demonstrated for other cancer entities [72, 73]. Of note, a 21-gene recurrence score based on gene expression significantly enhanced prediction of distant recurrence and chemotherapy benefit in breast cancer [72, 74, 75].

In the present work, we developed a reference-free subtype classification system for individual RCC samples using gene expression data of 174 defined subtype-specific genes (Additional file 6: Table S4). The herein described classification method is applicable to single tumor samples, and notably, normalization of gene expression data across cohorts and consideration of batch effects are not required. Its application has been extensively tested in different cohorts, with different tissue preparations and different technologies for expression quantification. Our approach is able to separate tumors across various subtypes that can be unambiguously assigned to one of the main histological subtypes [5] from molecularly heterogeneous tumors with overlapping molecular features. It allows the identification of tumors with clear cell and papillary features, which account for 29% of cases in the TCGA cohort, as well as recently described rare RCC subtypes (e.g., CIMP, Fig. 3A, B). We were able to show, using the TCGA RCC cohort, that even though PSA was originally based on pathological categories ccRCC, pRCC, and chRCC, it generates more information than pathological classification into these subtypes (Fig. 3D). The new information provided by PSA becomes specifically apparent when PSA and pathological classification are combined. In line with the novel 2022 WHO classification, subcategorization into pRCC type 1 and type 2 was no longer considered, which was additionally corroborated by a recent publication particularly addressing the co-occurrence of T1 and T2 features in pRCC [76]. Moreover, PSA applied to an independent histologically informed cohort (C4, *n* = 92) including 48 pRCC cases, enabled valid classification of main and heterogeneous RCC subtypes. Here, no significant differences in pRCC proportions between type 1 and type 2 cases were found supporting the novel WHO classification. Finally, to consider misclassification due to the presence of non-tumor renal tissue, PSA was assessed using TCGA data from non-tumor tissues. PSA identified unambiguously non-tumor tissue that differed constantly in composition from the assignments found in tumor samples.

Application of the PSA approach for characterization of RCC cell lines from the Cancer Cell Line Encyclopedia (CCLE) and the COSMIC Cell Lines Project (CCLP) confirms that the underlying signature genes derived from bulk tumor tissues indeed enable classification of pure tumor cells, and PSA is not impaired by e.g. stroma or immune infiltration. Notably, results from the new PSA based on only 174 genes is in line with findings from complex genomic characterization of cell lines by Sinha and colleagues [66].

Our computational approach of proportional assignment of molecular subtype features to individual RCC samples allows not only independent molecular classification, but the PSA-based RCC-R(isk) score enables also reliable prognosis and prediction of therapeutic outcome. Considering heterogeneity of RCC, of course, multiregion sequencing data for subtype prediction indicated some intratumor variability (Additional file 2: Fig. S14A), but the same risk was assigned for all samples derived from one tumor by the RCC-R score except for one case (Additional file 2: Fig. S14B). In general, the novel molecular classification based on PSA can be used to identify high-risk patients irrespective of the pathological classification even for personalized treatment strategies and innovative immunotherapeutic interventions. We could show that within heterogeneous tumors progression-free survival deviates significantly more between treatment arms in the JAVELIN Renal 101 and the IMmotion151 trials compared to the subgroup of unambiguous cases. These results indicate that molecular subtype composition represents valuable additional information for treatment strategies for RCC compared to histopathological-based characterization of RCC only. Thus, the PSA allows upfront selection of molecularly heterogeneous tumors which is clinically important for the selection of patients that may benefit from novel therapies and future drug trials. Further stratification of molecularly heterogeneous tumors would allow the identification of individual patients having a higher probability for good vs worse drug response, but future prospective studies are mandatory.

Moreover, because analyses of pathological images in the TCGA cohort indicated a higher complexity of morphology in heterogeneous tumors, in particular in the case of non-unique ccRCCs (Fig. 3F), further studies integrating additional Omics-data (e.g., metabolomics) are warranted to characterize underlying molecular mechanisms associated with the predicted RCC mixed type. In a first analysis for better molecular understanding, risk groups based on PSA were compared to recent classifications and cluster analyses [67, 70, 71] (Fig. 5G–I). Our results clearly indicate that subtype and risk prediction through our novel approach provided additional information not covered by recently published RCC classifications. In contrast to currently available gene expression risk scores such as ClearCode34 and S3-score [12, 14, 77], which are tailored to certain subtypes only (e.g., ccRCC), our RCC-R score evaluates the composition of molecular features from different histopathological subtypes for outcome prediction. Thus, our method only requires classification of a sample as RCC in general, whereas well-established risk scores require assignment to one of the RCC subtypes categories hindering a direct comparison [12, 14, 68, 78–80]. Further studies are warranted to prospectively evaluate the clinical utility of our new classification and risk prediction model as well as to consider complementary approaches integrating already available subtype-specific scores.

## Conclusions

In summary, we developed a computational deconvolution method for continuous molecular subtyping of individual RCC tissue samples across subtypes based on gene expression. Thereby, RCC cases with overlapping molecular features from different histological subtypes were uncovered. This novel concept enables subtyping and risk prediction of RCC for personalized treatment strategies irrespective of the pathological classification. Similar approaches might be considered for other tumor entities.

## Supplementary Information

**Additional file 1.** Supplementary Methods.

**Additional file 2.** Supplementary Figures 1-14.

**Additional file 3: Table S1.** Patient characteristics of cohort C1.

**Additional file 4: Table S2.** Composition of cohort C2.

**Additional file 5: Table S3.** PSA of RCC cell lines.

**Additional file 6: Table S4.** Signature genes.

**Additional file 7: Table S5.** PSA of the TCGA RCC cohort (C3).

## Data Availability

Data required to support the results and conclusions of the article are contained in the manuscript and/or Additional files 1, 2, 3, 4, 5, 6, and 7. Individual genome-wide transcriptome data generated from fresh-frozen tissue in the current study has been deposited in the European Genome-Phenome Archive (EGA) (www.ebi.ac.uk/ega/home), which is hosted by the EBI and the CRG. The data accession number is EGAS00001001176 [81]. Additional public repositories used for downloading of data analyzed within this study have been listed the section “Methods” and the Additional file 1: Supplementary methods.

## References

[1] Scelo G, Larose TL (2018). Epidemiology and risk factors for kidney cancer. J Clin Oncol.

[2] Linehan WM, Ricketts CJ (2019). The Cancer Genome Atlas of renal cell carcinoma: findings and clinical implications. Nat Rev Urol.

[3] Rini BI, Plimack ER, Stus V, Gafanov R, Hawkins R, Nosov D (2019). Pembrolizumab plus axitinib versus sunitinib for advanced renal-cell carcinoma. N Engl J Med.

[4] Motzer RJ, Rini BI, McDermott DF, Arén Frontera O, Hammers HJ, Carducci MA (2019). Nivolumab plus ipilimumab versus sunitinib in first-line treatment for advanced renal cell carcinoma: extended follow-up of efficacy and safety results from a randomised, controlled, phase 3 trial. Lancet Oncol.

[5] Moch H, Cubilla AL, Humphrey PA, Reuter VE, Ulbright TM (2016). The 2016 WHO classification of tumours of the urinary system and male genital organs-part A: renal, penile, and testicular tumours. Eur Urol.

[6] Ljungberg B, Albiges L, Abu-Ghanem Y, Bensalah K, Dabestani S, Fernández-Pello S (2019). European Association of Urology guidelines on renal cell carcinoma: the 2019 update. Eur Urol.

[7] McKay RR, Bossé D, Choueiri TK (2018). Evolving systemic treatment landscape for patients with advanced renal cell carcinoma. J Clin Oncol.

[8] Choueiri TK, Kaelin WG (2020). Targeting the HIF2–VEGF axis in renal cell carcinoma. Nat Med.

[9] Motzer RJ, Robbins PB, Powles T, Albiges L, Haanen JB, Larkin J (2020). Avelumab plus axitinib versus sunitinib in advanced renal cell carcinoma: biomarker analysis of the phase 3 JAVELIN Renal 101 trial. Nat Med.

[10] Choueiri TK, Atkins MB, Bakouny Z, Carlo MI, Drake CG, Jonasch E (2020). Summary from the first Kidney Cancer Research Summit, September 12–13, 2019: a focus on translational research. J Natl Cancer Inst..

[11] Graham J, Dudani S, Heng DYC (2018). Prognostication in kidney cancer: recent advances and future directions. J Clin Oncol.

[12] Büttner F, Winter S, Rausch S, Reustle A, Kruck S, Junker K (2015). Survival prediction of clear cell renal cell carcinoma based on gene expression similarity to the proximal tubule of the nephron. Eur Urol.

[13] Rini B, Goddard A, Knezevic D, Maddala T, Zhou M, Aydin H (2015). A 16-gene assay to predict recurrence after surgery in localised renal cell carcinoma: development and validation studies. Lancet Oncol.

[14] Brooks SA, Brannon AR, Parker JS, Fisher JC, Sen O, Kattan MW (2014). ClearCode34: a prognostic risk predictor for localized clear cell renal cell carcinoma. Eur Urol.

[15] Srigley JR, Cheng L, Grignon DJ, Moch H, Humphrey PA, Ulbright TM (2016). Clear cell papillary renal cell carcinoma. WHO classification of tumours of the urinary system and male genital organs.

[16] Kuroda N, Ohe C, Kawakami F, Mikami S, Furuya M, Matsuura K (2014). Clear cell papillary renal cell carcinoma: a review. Int J Clin Exp Pathol.

[17] Raspollini MR, Moch H, Tan PH, Amin MB, Turajlic S. Renal cell tumors. In: WHO Classification of Tumours Editorial Board. Urinary and male genital tumours [Internet]. 2022. https://tumourclassification.iarc.who.int/chapters/36. Accessed 3 Aug 2022.

[18] Gerlinger M, Rowan AJ, Horswell S, Math M, Larkin J, Endesfelder D (2012). Intratumor heterogeneity and branched evolution revealed by multiregion sequencing. N Engl J Med.

[19] Moch H, Amin MB, Berney DM, Compérat EM, Gill AJ, Hartmann A (2022). The 2022 World Health Organization classification of tumours of the urinary system and male genital organs-part A: renal, penile, and testicular tumours. Eur Urol.

[20] Yusenko MV, Kuiper RP, Boethe T, Ljungberg B, van Kessel AG, Kovacs G (2009). High-resolution DNA copy number and gene expression analyses distinguish chromophobe renal cell carcinomas and renal oncocytomas. BMC Cancer.

[21] Tan M-H, Wong CF, Tan HL, Yang XJ, Ditlev J, Matsuda D (2010). Genomic expression and single-nucleotide polymorphism profiling discriminates chromophobe renal cell carcinoma and oncocytoma. BMC Cancer.

[22] Peña-Llopis S, Vega-Rubín-de-Celis S, Liao A, Leng N, Pavía-Jiménez A, Wang S (2012). BAP1 loss defines a new class of renal cell carcinoma. Nat Genet.

[23] Furge KA, Chen J, Koeman J, Swiatek P, Dykema K, Lucin K (2007). Detection of DNA copy number changes and oncogenic signaling abnormalities from gene expression data reveals MYC activation in high-grade papillary renal cell carcinoma. Cancer Res.

[24] Ho TH, Serie DJ, Parasramka M, Cheville JC, Bot BM, Tan W (2017). Differential gene expression profiling of matched primary renal cell carcinoma and metastases reveals upregulation of extracellular matrix genes. Ann Oncol.

[25] Koeman JM, Russell RC, Tan M-H, Petillo D, Westphal M, Koelzer K (2008). Somatic pairing of chromosome 19 in renal oncocytoma is associated with deregulated EGLN2-mediated corrected oxygen-sensing response. PLoS Genet.

[26] Lang H, Béraud C, Bethry A, Danilin S, Lindner V, Coquard C (2016). Establishment of a large panel of patient-derived preclinical models of human renal cell carcinoma. Oncotarget.

[27] Comprehensive molecular characterization of clear cell renal cell carcinoma. Nature. 2013;499:43–9. 10.1038/nature12222. 10.1038/nature12222 PMC3771322 23792563

[28] Network CGAR, Linehan WM, Spellman PT, Ricketts CJ, Creighton CJ, Fei SS (2016). Comprehensive molecular characterization of papillary renal-cell carcinoma. N Engl J Med.

[29] Davis CF, Ricketts CJ, Wang M, Yang L, Cherniack AD, Shen H (2014). The somatic genomic landscape of chromophobe renal cell carcinoma. Cancer Cell.

[30] Liu J, Lichtenberg T, Hoadley KA, Poisson LM, Lazar AJ, Cherniack AD (2018). An integrated TCGA pan-cancer clinical data resource to drive high-quality survival outcome analytics. Cell.

[31] Ellrott K, Bailey MH, Saksena G, Covington KR, Kandoth C, Stewart C (2018). Scalable open science approach for mutation calling of tumor exomes using multiple genomic pipelines. Cell Syst.

[32] Ricketts CJ, de Cubas AA, Fan H, Smith CC, Lang M, Reznik E (2018). The Cancer Genome Atlas comprehensive molecular characterization of renal cell carcinoma. Cell Rep.

[33] Polifka I, Agaimy A, Herrmann E, Spath V, Trojan L, Stöckle M (2019). High proliferation rate and TNM stage but not histomorphological subtype are independent prognostic markers for overall survival in papillary renal cell carcinoma. Hum Pathol.

[34] Motzer RJ, Penkov K, Haanen J, Rini B, Albiges L, Campbell MT (2019). Avelumab plus axitinib versus sunitinib for advanced renal-cell carcinoma. N Engl J Med.

[35] Rini BI, Powles T, Atkins MB, Escudier B, McDermott DF, Suarez C (2019). Atezolizumab plus bevacizumab versus sunitinib in patients with previously untreated metastatic renal cell carcinoma (IMmotion151): a multicentre, open-label, phase 3, randomised controlled trial. Lancet.

[36] Motzer RJ, Banchereau R, Hamidi H, Powles T, McDermott D, Atkins MB (2020). Molecular subsets in renal cancer determine outcome to checkpoint and angiogenesis blockade. Cancer Cell.

[37] Broad Institute and Novartis Institutes for Biomedical Research. Broad-Novartis Cancer Cell Line Encyclopedia. www.broadinstitute.org/ccle.

[38] Ghandi M, Huang FW, Jané-Valbuena J, Kryukov GV, Lo CC, McDonald ER (2019). Next-generation characterization of the Cancer Cell Line Encyclopedia. Nature.

[39] COSMIC Cell Lines project. http://cancer.sanger.ac.uk/cell_lines.

[40] Tate JG, Bamford S, Jubb HC, Sondka Z, Beare DM, Bindal N (2019). COSMIC: the catalogue of somatic mutations in cancer. Nucleic Acids Res.

[41] Fisel P, Kruck S, Winter S, Bedke J, Hennenlotter J, Nies AT (2013). DNA methylation of the SLC16A3 promoter regulates expression of the human lactate transporter MCT4 in renal cancer with consequences for clinical outcome. Clin Cancer Res.

[42] Winter S, Fisel P, Büttner F, Rausch S, D'Amico D, Hennenlotter J (2016). Methylomes of renal cell lines and tumors or metastases differ significantly with impact on pharmacogenes. Sci Rep.

[43] R Core Team. R: a language and environment for statistical computing 2020. Vienna, Austria. https://www.r-project.org/

[44] Kampstra P (2008). Beanplot: a boxplot alternative for visual comparison of distributions. J Stat Software Code Snippets.

[45] Hothorn T, Hornik K, van de Wiel M, Zeileis A (2008). Implementing a class of permutation tests: the coin package. J Stat Software Art.

[46] Venables WN, Ripley BD (2002). Modern applied statistics with S.

[47] Hothorn T, Zeileis A (2015). partykit: a modular toolkit for recursive partytioning in R. J Mach Learn Res.

[48] Hothorn T, Hornik K, Zeileis A (2006). Unbiased recursive partitioning: a conditional inference framework. J Comput Graph Stat.

[49] Papadakis M, Tsagris M, Dimitriadis M, Fafalios S, Tsamardinos I, Fasiolo M (2020). Rfast: a collection of efficient and extremely fast R functions.

[50] Harrell FE (2019). rms: regression modeling strategies.

[51] Eklund AC (2020). squash: color-based plots for multivariate visualization.

[52] Therneau TM (2020). A package for survival analysis in R.

[53] Dowd C (2018). twosamples: fast permutation based two sample tests.

[54] Davis S, Meltzer PS (2007). GEOquery: a bridge between the Gene Expression Omnibus (GEO) and BioConductor. Bioinformatics.

[55] Ritchie ME, Phipson B, Wu D, Hu Y, Law CW, Shi W, Smyth GK (2015). limma powers differential expression analyses for RNA-sequencing and microarray studies. Nucleic Acids Res.

[56] Carvalho BS, Irizarry RA (2010). A framework for oligonucleotide microarray preprocessing. Bioinformatics.

[57] Carlson M (2019). org.Hs.eg.db: genome wide annotation for Human.

[58] MacDonald JW (2017). pd.hta.2.0: platform design info for Affymetrix HTA-2_0.

[59] Hansen KD, Gentry J, Long L, Gentleman R, Falcon S, Hahne F, Sarkar D (2019). Rgraphviz: provides plotting capabilities for R graph objects.

[60] Piccolo SR, Sun Y, Campbell JD, Lenburg ME, Bild AH, Johnson WE (2012). A single-sample microarray normalization method to facilitate personalized-medicine workflows. Genomics.

[61] Morgan M, Obenchain V, Hester J, Pagès H (2019). SummarizedExperiment: SummarizedExperiment container.

[62] Colaprico A, Silva TC, Olsen C, Garofano L, Cava C, Garolini D (2016). TCGAbiolinks: an R/Bioconductor package for integrative analysis of TCGA data. Nucleic Acids Res.

[63] Dai M, Wang P, Boyd AD, Kostov G, Athey B, Jones EG (2005). Evolving gene/transcript definitions significantly alter the interpretation of GeneChip data. Nucleic Acids Res.

[64] Brainarray. http://brainarray.mbni.med.umich.edu.

[65] Holm S (1979). A simple sequentially rejective multiple test procedure. Scand J Stat.

[66] Sinha R, Winer AG, Chevinsky M, Jakubowski C, Chen Y-B, Dong Y (2017). Analysis of renal cancer cell lines from two major resources enables genomics-guided cell line selection. Nat Commun.

[67] Chen F, Zhang Y, Şenbabaoğlu Y, Ciriello G, Yang L, Reznik E (2016). Multilevel genomics-based taxonomy of renal cell carcinoma. Cell Rep.

[68] Fu Y, Jung AW, Torne RV, Gonzalez S, Vöhringer H, Shmatko A (2020). Pan-cancer computational histopathology reveals mutations, tumor composition and prognosis. Nat Cancer.

[69] Royston P, Altman DG (2013). External validation of a Cox prognostic model: principles and methods. BMC Med Res Methodol.

[70] Yoshihara K, Shahmoradgoli M, Martínez E, Vegesna R, Kim H, Torres-Garcia W (2013). Inferring tumour purity and stromal and immune cell admixture from expression data. Nat Commun.

[71] Clark DJ, Dhanasekaran SM, Petralia F, Pan J, Song X, Hu Y (2019). Integrated proteogenomic characterization of clear cell renal cell carcinoma. Cell.

[72] Paik S, Shak S, Tang G, Kim C, Baker J, Cronin M (2004). A multigene assay to predict recurrence of tamoxifen-treated, node-negative breast cancer. N Engl J Med.

[73] Schmitz R, Wright GW, Huang DW, Johnson CA, Phelan JD, Wang JQ (2018). Genetics and pathogenesis of diffuse large B-cell lymphoma. N Engl J Med.

[74] Sparano JA, Gray RJ, Della Makower F, Pritchard KI, Albain KS, Hayes DF (2015). Prospective validation of a 21-gene expression assay in breast cancer. N Engl J Med.

[75] Sparano JA, Gray RJ, Ravdin PM, Della Makower F, Pritchard KI, Albain KS (2019). Clinical and genomic risk to guide the use of adjuvant therapy for breast cancer. N Engl J Med.

[76] Murugan P, Jia L, Dinatale RG, Assel M, Benfante N, Al-Ahmadie HA (2021). Papillary renal cell carcinoma: a single institutional study of 199 cases addressing classification, clinicopathologic and molecular features, and treatment outcome. Mod Pathol.

[77] Büttner F, Winter S, Rausch S, Hennenlotter J, Kruck S, Stenzl A (2018). Clinical utility of the S3-score for molecular prediction of outcome in non-metastatic and metastatic clear cell renal cell carcinoma. BMC Med.

[78] Marostica E, Barber R, Denize T, Kohane IS, Signoretti S, Golden JA, Yu K-H (2021). Development of a histopathology informatics pipeline for classification and prediction of clinical outcomes in subtypes of renal cell carcinoma. Clin Cancer Res.

[79] Yao M, Huang Y, Shioi K, Hattori K, Murakami T, Sano F (2008). A three-gene expression signature model to predict clinical outcome of clear cell renal carcinoma. Int J Cancer.

[80] Zhao H, Ljungberg B, Grankvist K, Rasmuson T, Tibshirani R, Brooks JD (2006). Gene expression profiling predicts survival in conventional renal cell carcinoma. PLoS Med.

[81] TuSCo. Study of renal cancers and renal cancer metastases. https://ega-archive.org/studies/EGAS00001001176.

[82] Li P, Conley A, Zhang H, Kim HL (2014). Whole-transcriptome profiling of formalin-fixed, paraffin-embedded renal cell carcinoma by RNA-seq. BMC Genomics.

