# Supplementary material for: A novel molecular signature identifies mixed subtypes in renal cell carcinoma with poor prognosis and independent response to immunotherapy
Source: Genome Med. 2022 Sep 15;14:105. doi: 10.1186/s13073-022-01105-y (PMC9476269; doi:10.1186/s13073-022-01105-y)
Supplement: Supplementary file 1 — Additional file 1. Supplementary Methods. [file 13073_2022_1105_MOESM1_ESM.pdf]

Additional file 1: Supplementary Methods

Patient cohorts

Regarding the TCGA RCC cohort a pathological re-evaluation of 843 tumors was obtained from Ricketts et al. [1]. Patients receiving prior treatment related to their disease were excluded. Four patients of the KIRC cohort were represented by several samples in the expression data set. Here, the sample with highest median expression was chosen, respectively. To ensure that only tumor samples were included, remaining tumor and non-tumor samples from C3 were hierarchically clustered using Ward's method (data not shown). Three cases (TCGA-BQ-5889, TCGA-CJ-5683, and TCGA-DV-5573) wrongly assigned as tumor tissues were excluded. In case of TCGA-CW-5591 labels of tumor and non-tumor data had been mixed up.

Preprocessing of gene expression data

Total RNA was isolated from fresh-frozen tissue of cohorts C1, C2, and C5 as previously described [2, 3]. RNA from FFPE tissue of cohort C4 was isolated using the AllPrep DNA/RNA FFPE Kit (Qiagen, Germany). Genome-wide transcriptome analyses were performed using GeneChip™ Human Transcriptome Array 2.0 (Thermo Fisher Scientific). Processing of microarray data was performed as described [3]. Array quality control was conducted by Affymetrix Expression Console™ (Build 1.4.1.46; Thermo Fisher Scientific). The microarrays from C1 were preprocessed together using the Robust Multiarray Average (RMA) implementation from the R-package oligo. Microarrays from C2, C4 and C5 were normalized individually using the SCAN method

from R-package SCAN.UPC. The data accession number at the European Genome-phenome Archive (www.ebi.ac.uk/ega/home), which is hosted by the EBI and the CRG, is EGAS00001001176.

Genome-wide transcriptome data produced by the GeneChip™ Human Genome U133 Plus 2.0 microarray (Thermo Fisher Scientific) for the 158 RCC patients [4-10] of C2 were downloaded from Gene Expression Omnibus (GEO) using R-package GEOquery (Additional File 4. Table S2). Microarrays from C2 were normalized individually using the SCAN method from the R-package SCAN.UPC. For all microarrays, probe sets were summarized at Entrez GeneID level using the annotation provided by brainarray for the respective chip type.

Ensembl gene identifiers used in TCGA gene expression data (“FPKM-UQ”) were mapped to Entrez GeneIDs using the annotation provided by the org.Hs.eg.db R-package.

HUGO gene symbols used in gene expression data from the JAVELIN Renal 101 trial [11] were mapped to Entrez GeneIDs using the annotation provided by the org.Hs.eg.db R package.

Li et al. [12] compared whole-transcriptome profiles based on RNA-Seq using matched FFPE and fresh-frozen (FF) samples. Unmapped sequencing data were downloaded from the Sequence Read Archive (SRP050335) and were processed using the nf-core/rna-seq pipeline (version 1.4.2) [13]. We used the GENCODE gene annotation (version 22), which was also used for generation of the TCGA expression data. Entrez GeneIDs were obtained using the org.Hs.eg.db R package. PSA were calculated using FPKM values computed by StringTie [14].

Gene expression deconvolution using robust linear regression

Samples from RCC tissue were considered as composite samples that may combine specific molecular features from ccRCC, pRCC and chRCC. Further, it was assumed that the proportional composition of the subtypes is reflected in the gene expression profile of RCC samples. Gene expression deconvolution is a method that allows estimating the proportion of different cell types in heterogeneous samples [15,16]. The expression of a given gene in a RCC sample A was modeled as weighted sum of the expression of this gene in ccRCC, pRCC and chRCC. The weights correspond to the proportional composition of A and therefore are the same for all genes. Mathematically, the objective of deconvolution is to find a solution to the system of linear equations: $m = f \times S$. Here, the vector f of coefficients represents the unknown proportions to be determined for a potentially heterogeneous sample A. Gene expression levels of the signature genes in A are denoted by m. S is a signature matrix including the (median) expression values of the signature genes in ccRCC, pRCC, and chRCC. The matrix equation can be solved for f using standard linear least squares regression [17]. Robust linear regression as implemented in the "rlm" function from the R-package MASS (parameter maxit was set to 200) was used to increase stability of assignments. Expression deconvolution was performed on linear, i.e. non-log2-transformed, expression data as suggested by Zhong and Liu [18]. Log2 expression values from microarray analysis were therefore inverted to linear space. Raw counts from RNA-Seq measurement had to be normalized for sequencing depth and gene length. Linear expression values of the signature genes were centered to zero mean and scaled to unit variance preceding deconvolution. The proportional subtype

assignment (PSA) was computed by setting negative coefficients to 0 and dividing the three coefficients by their sum, such that $c + p + h = 100\%$, with $c, p,$ and h representing the ccRCC, pRCC, and chRCC proportion, respectively. Subsequently, percentages were rounded to integers.

A permutation P-value (P_{psa}) was calculated to assess the specificity of the signature for a certain tumor sample. Basically, the P-value computation was carried out in the same way as described in Newman et al. [19]. Briefly, Pearson's correlation coefficient R between m and $f \times S$ was compared against a derived null distribution R^* for sample A. Expression values in m were replaced by randomly drawn values from the full transcriptome of A, denoted $m_i{}^*$. Subtype proportions $f_i{}^*$ were determined for $m_i{}^*$ by deconvolution and the correlation coefficient between $m_i{}^*$ and $f_i{}^* \times S$ was calculated. This process was repeated 9,999 times, yielding R^*, and the P_{psa} was determined by the proportion of correlation coefficients in R^* that were greater than R, i.e. $P_{psa} = (|R^* > R| + 1)/(9999 + 1)$.

Generation of the gene signature

The samples of C1, which were evaluated by two teams of pathologists, were considered as unambiguous cases of ccRCC, pRCC and chRCC, respectively (Additional file 2. Fig. S1). Initially, expression measurements of 32,749 genes were available for C1 based on the annotation provided by brainarray for Human Transcriptome Array 2.0. Expression data were sample-wise centered to the median expression level of C1. Genes with median expression below the cohort median in each of the three subtypes were removed. Further, genes not covered by the Human

Genome U133 Plus 2.0 microarray according to the brainarray annotation or in the RNA expression data from TCGA were excluded.

It has been shown using TCGA data that RCC subtypes vary in tumor purity, see http://bioinformatics.mdanderson.org/estimate/. This pattern could be observed in C1 as well (Additional file 2. Fig. S2A). Therefore, genes stronger related to tumor purity as determined by the ESTIMATE method than to tumor type were removed to minimize the influence of tumor purity on the determination of PSA. To be precise, for each gene linear regression models were fit incorporating either tumor type or tumor purity as single predictor or both of them in a multiple regression model. In the latter case, models with and without interaction effects were considered. The reduction in the residual sum of squares was compared by analysis of deviance tests. Per variable, i.e. purity or type, the model with single predictor was tested vs. both corresponding multiple regression models and the lower of the two resulting P-values was used. A gene was kept in case its expression in C1 could be better explained by tumor type than by tumor purity (Additional file 2. Fig. S2B). 11,195 genes remained after these filtering steps (Additional file 2. Fig. S2C) and were subsequently tested for subtype-specific expression by analysis of variance. 5,811 genes showed significant variation between subtypes after correction for multiple testing using Holm's method [20]. From this set, subtype-specific genes were determined using Tukey's range test. If expression differed significantly in two of the three pairwise comparisons between subtypes, a gene was considered subtype-specific, resulting in 1,379 ccRCC-, 844 pRCC, and 1,463 chRCC-specific genes (total 3,686). Expression values of the candidate genes in C1 were collapsed by taking the median per subtype. For each candidate, the minimum log2 fold

change compared to the two respective other entities was calculated using the absolute values of the log2 fold changes (Additional file 2. Fig. S2D). Finally, genes were ordered by decreasing log2 fold change per subtype and expression values were transformed to linear space.

Next, the signature genes were selected from the set of subtype-specific genes. The expression of the signature genes in ccRCC, pRCC, and chRCC constituted the signature matrix S. As in similar studies [17, 19, 21], various signature matrices were tested (Additional file 2. Fig. S3). The top n genes with the highest log2 fold change per subtype were combined into a signature matrix S_n, i.e. S_n included $3n$ different genes. Each matrix S_n was used to deconvolve the 170 samples of C2. n was iterated from 2 to 500 and for $n > 2$ the maximum absolute difference (MAD), i.e. $max(\{|c_n - c_{n-1}|, |p_n - p_{n-1}|, |h_n - h_{n-1}|\})$, in the PSA between two consecutive signature matrices S_n and S_{n-1} was calculated per sample.

Figure S3A (Additional file 2) shows the 0.95-quantiles of the MAD values for increasing matrix size. Between smaller matrices, relatively large alterations in the PSA could be observed. Not every added gene triplet led to substantial changes and with increasing matrix size, clear changes became less frequent. The final set of signature genes was determined using a heuristic approach. Based on the assumption that more genes allow for a more precise and stable estimation, the largest matrix was chosen inducing a relevant MAD (MAD > 5%) in the PSA for a substantial proportion of tumors. C2 was resampled 300 times with replacement to simulate different cohort compositions. S_{58}, including 174 genes, was the largest matrix significantly changing a substantial proportion (mean proportion of tumors with MAD > 5%: 8.3%) of assignments relative to

its predecessor matrix and therefore has been chosen as signature matrix (Additional file 2. Fig. S3B; Additional file 6. Table S4).

Variability in gene expression explained by subtype classifications

We assumed that differences between histological subtypes are comprehensively reflected by gene expression levels. Consequently, the information contained in PSA or pathological classification can be assessed based on the variance in expression data explained by these factors. To this end, a principal variance component analysis-like approach was applied [22]. First eigenvalues and eigenvectors of the covariance matrix of gene-wise mean-centered expression data were determined. Subsequently, linear regression was performed for each eigenvector with pathological categorization or PSA as explanatory variable. Subsequently, coefficients of determination (R-squared) from the resulting regression models were used to weight the eigenvalues correspondingly. Dividing the sum of weighted eigenvalues by the total variance (i.e. the sum of the unweighted eigenvalues) resulted in the proportion of the explained variance for a certain variable.

The RCC population, here including ccRCC, pRCC, and chRCC from C3, was modeled using the prevalence of the subtypes reported in the recent edition of the WHO classification of tumors [23]. Based on the given ranges of frequencies (60-75% ccRCC, 13-20% pRCC, 5-7% chRCC), we set 68% for ccRCC, 17% for pRCC, and 6% for chRCC. From 805 tumors with available pathological classification [13] and PSA ($P_{psa} < 0.05$) we used all 466 ccRCC, resulting in group sizes of 116 for pRCC and 41 for chRCC according to their respective frequencies. The resulting gene expression matrix

for variance analysis consisted of expression levels of 25,208 genes in 623 tumors. In order to generate stable estimates different RCC populations were simulated by sampling 500 times with replacement from the set of 805 tumors in accordance with the group sizes mentioned above (Fig. 3D). FPKM-UQ expression values were log2-transformed (log2(x+1)).

Relationship between PSA and the occurrence of somatic mutations

The analysis of somatic mutations in C3 was limited to 344 genes [24] frequently mutated in RCC subtypes. Further, genes classified as FLAGS [25] were excluded, resulting in 310 genes with somatic mutation calls in 620 samples. Notably, the bi-allelic loss status of e.g. VHL, TP53 was not considered in this analysis. For each gene and subtype, the distribution of assigned proportions was compared between tumors with and without a somatic mutation using the Cramer-von Mises test as implemented in the R-package twosamples (Fig. S6). Permutation P-values were determined based on 10,000 bootstrap iterations.

Relationship between PSA and computational histopathology.

In the study of Fu et al. [26], tissue slides from 28 TCGA cohorts were cropped into tiles and 1,536 computational histopathological features were extracted for each tile by a deep convolutional neural network. The features of 50 randomly selected tiles per tissue slide were publicly available. We used the subset of slides that were from tumors with high tumor purity, which included 729 slides from 396 KIRC tumors, 237 slides from 187 KIRP tumors, and 118 slides from 65 KICH tumors.

Using the histopathologic features, the Manhattan distance between tiles from the same slide as well as from different slides was calculated. We found that the mean distance between tiles of the same slide was of similar size as the respective mean distance between tiles of different slides from the same tumor (Additional file 2. Fig. S7). The mean pairwise Manhattan distance between the 50 tiles per tumor tissue slide was used as a measure of histopathological complexity.

Relationship between PSA and patient survival

Our approach introduced here estimates three percentage values per sample, representing the predicted proportions of ccRCC, pRCC, and chRCC. Hereinafter, the terms "proportion" and "score" are used interchangeably. The relationship between numerical subtype scores and cancer-specific survival (CSS) was investigated in cohort C3. Linear predictors from Cox proportional hazard models were used as prognostic index (PI). Consequently, the PI for a patient is the log relative hazard compared to a hypothetical individual with a PI of 0 and the log hazard ratio between two patients was derived from the difference in their PI.

First, univariate analyses using ccRCC, pRCC, as well as chRCC proportions as single predictors were performed, respectively (Additional file 2. Fig. S8). The proportions were modeled via restricted cubic splines (RCS) using the "rcs" function from the R-package rms. RCS are flexible functions that produce smooth fits between outcome and predictor and are particularly useful when the type of relationship is unknown. The predictor variable is subdivided into segments using a set of knots, and a cubic polynomial is fitted in each segment. Here, smooth joints at the knots and linearity in the two tails are

required. Each time a variable was transformed with RCS, three, four, and five knots were pre-tested using the Akaike information criterion (AIC), as suggested by Harrell [27]. As only two of the subtype scores are independent (since all three sum up to 100%), the chRCC-score which exhibited the lowest variability (Additional file 2. Fig. S8A) as well as the weakest association to survival (Fig. S8B) was excluded from the following analyses. Figure S8B (Additional file 2) shows that tumors with medium scale proportions of ccRCC and pRCC had the highest risk. The curves peaked at 39% (ccRCC) and 58% (pRCC), respectively, and with in-/decrease of the respective proportion the risk decreased for both subtype scores. The shape of both graphs suggested a cubic relationship to the log relative hazard. Hence, both scores were additionally modeled via cubic polynomials. The estimates from the RCS regression and the cubic polynomial regression were within the standard error of the respective other fit (Additional file 2. Fig. S10). Moreover, cubic polynomials and corresponding RCS yielded very similar predictions over the entire range with a maximum absolute difference in the PI of 0.0631 (ccRCC-score) and 0.0225 (pRCC-score), respectively.

In the survival analysis of cohort C3 (see Fig. 5A-C), ccRCC- and pRCC-score were used in (additive) combination in Cox regression analysis, with both being modeled using RCS. For 17 tumors of C3 for which survival data were not available the PI was predicted using the coefficients from the fitted Cox model.

Risk prediction of RCC using PSA and development of the RCC-R score

The predictive accuracy of ccRCC- and pRCC-score as well as their combination was compared by 10-fold cross validation (CV) in cohort C3. Modeling with RCS as well as

with cubic polynomials was tested. As suggested by Dai and Breheny [28], the CV was applied to the linear predictors from the Cox models. To be more precise, for each fold a Cox model was fitted on the training set and then applied to estimate linear predictors on the test set. The linear predictors of all 10 test sets were pooled together to construct a set of linear predictors for the whole cohort. A partial likelihood was then obtained by regression of CSS on the combined set of linear predictors. Since the folds were selected at random, the CV was repeated 300 times to obtain stable estimates of the predictive accuracy for each tested model (Additional file 2. Fig. S11A). For comparison, the pathological classification into ccRCC, pRCC, and chRCC was evaluated as categorical predictor of survival. Cubic polynomial models were of equal or better quality compared to their RCS counterparts. Therefore, results from RCS fits are not discussed in the following. As expected, the pRCC-score achieved less predictive accuracy than the ccRCC-score. This is due to the fact that the pRCC-score assigns low or zero proportions to ccRCC as well as chRCC samples (Fig. 3B), which however differ strongly in their survival rates. In contrast, the ccRCC-score equates pRCC and chRCC tissues (Fig. 3A), which are more similar in terms of CSS. The (additive) combination of both scores turned out to be less suitable for risk prediction in unknown data because of its higher model complexity. Pathological classification performed worse than the PSA-based predictors. Hence, the ccRCC-score modeled via cubic polynomials, hereafter termed RCC-R score, showed the best trade-off between model complexity and predictive accuracy and consequently was selected as biomarker for risk prediction. With PSA specified on 0 – 1 scale, the PI for a RCC sample with a RCC-R score or ccRCC proportion of $\boldsymbol{c}$ was determined as follows:

$$\boldsymbol{PI = c \times 14.71 - c^2 \times 25.46 + c^3 \times 12.21 - 1.46} \qquad \text{(eq. 1)}$$

By subtracting 1.46, tumors with 100% ccRCC proportion, i.e. $\boldsymbol{c}$ = 1, are given a PI value of 0. The coefficients were estimated for endpoint CSS using 828 tumors of C3 for which both survival data and PSA were available ($P_{psa} < 0.05$) (red curve in Additional file 2. Fig. SA10, Fig. 5D).

External validation of the RCC-R score

The validation of the RCC-R score as a risk score followed the proposals of Royston and Altman [29]. The validation cohort C5 comprised 242 RCC samples (Additional file 2. Fig. S12A). First, the PI in C5 (PI_{C5}) was constructed using equation 1 for 241 tumors from C5 with $P_{psa} < 0.05$. Discrimination, i.e. the ability of the model to distinguish between patients with different risks, was evaluated by regression of CSS on PI_{C5}. The resulting calibration slope was 1.106 (SE = 0.265) and thus not significantly different from 1 (P = 0.69 by z-test) indicating that discrimination was preserved in C5. The concordance between observed outcome and prediction, i.e. the calibration of the model, was assessed by comparing Kaplan-Meier estimates for C5 with predicted survival probabilities. First, risk groups were formed in C3. This was done by categorizing the PI using conditional inference trees (R-package partykit, function “ctree”) with endpoint CSS. Three risk groups with significantly different survival functions were identified in this way (Fig. 5D). The corresponding cutoff values were then used to subdivide C5 based on PI_{C5} (Additional file 2. Fig. S12B). Next, CSS probabilities were predicted for patients from C5. Five patients from C5 with follow-up

time exceeding the maximal follow-up time in C3 (16.2 years) were disregarded here. Using PI_{C5} and the baseline survival function from the Cox model evaluated in cohort C3, estimates of survival probabilities along with pointwise 95% prediction intervals were calculated for each risk group in C5 at half-yearly intervals from 0 to 16 years (Additional file 2. Fig. S12C). Comparison of Kaplan-Meier estimates for the risk groups in C5 with the predicted CSS probabilities showed that the model slightly underpredicted the good group. In contrast, the probabilities of the poor group were predicted to be higher, especially in the first years after surgery. Predicted survival probabilities of the intermediate group matched very well with the Kaplan-Meier estimate. Taken together, good discrimination and calibration in C5 indicated a successful external validation of the RCC-R score model.

References

1. Ricketts CJ, Cubas AA de, Fan H, Smith CC, Lang M, Reznik E, et al. The Cancer Genome Atlas Comprehensive Molecular Characterization of Renal Cell Carcinoma. Cell Rep. 2018;23:313-326.e5. doi:10.1016/j.celrep.2018.03.075.
2. Fisel P, Kruck S, Winter S, Bedke J, Hennenlotter J, Nies AT, et al. DNA Methylation of the SLC16A3 Promoter Regulates Expression of the Human Lactate Transporter MCT4 in Renal Cancer with Consequences for Clinical Outcome. Clinical Cancer Research. 2013;19:5170–81. doi:10.1158/1078-0432.CCR-13-1180.
3. Winter S, Fisel P, Büttner F, Rausch S, D'Amico D, Hennenlotter J, et al. Methylomes of renal cell lines and tumors or metastases differ significantly with impact on pharmacogenes. Sci Rep. 2016;6:29930. doi:10.1038/srep29930.
4. Yusenko MV, Kuiper RP, Boethe T, Ljungberg B, van Kessel AG, Kovacs G. High-resolution DNA copy number and gene expression analyses distinguish chromophobe renal cell carcinomas and renal oncocytomas. BMC Cancer. 2009;9:152. doi:10.1186/1471-2407-9-152.
5. Tan M-H, Wong CF, Tan HL, Yang XJ, Ditlev J, Matsuda D, et al. Genomic expression and single-nucleotide polymorphism profiling discriminates chromophobe renal cell carcinoma and oncocytoma. BMC Cancer. 2010;10:196. doi:10.1186/1471-2407-10-196.
6. Peña-Llopis S, Vega-Rubín-de-Celis S, Liao A, Leng N, Pavía-Jiménez A, Wang S, et al. BAP1 loss defines a new class of renal cell carcinoma. Nat. Genet. 2012;44:751–9. doi:10.1038/ng.2323.
7. Furge KA, Chen J, Koeman J, Swiatek P, Dykema K, Lucin K, et al. Detection of DNA copy number changes and oncogenic signaling abnormalities from gene expression data reveals MYC activation in high-grade papillary renal cell carcinoma. Cancer Res. 2007;67:3171–6. doi:10.1158/0008-5472.CAN-06-4571.
8. Ho TH, Serie DJ, Parasramka M, Cheville JC, Bot BM, Tan W, et al. Differential gene expression profiling of matched primary renal cell carcinoma and metastases reveals upregulation of extracellular matrix genes. Ann. Oncol. 2017;28:604–10. doi:10.1093/annonc/mdw652.
9. Koeman JM, Russell RC, Tan M-H, Petillo D, Westphal M, Koelzer K, et al. Somatic pairing of chromosome 19 in renal oncocytoma is associated with deregulated EGLN2-mediated corrected oxygen-sensing response. PLoS Genet. 2008;4:e1000176. doi:10.1371/journal.pgen.1000176.
10. Lang H, Béraud C, Bethry A, Danilin S, Lindner V, Coquard C, et al. Establishment of a large panel of patient-derived preclinical models of human renal cell carcinoma. Oncotarget. 2016;7:59336–59. doi:10.18632/oncotarget.10659.
11. Motzer RJ, Robbins PB, Powles T, Albiges L, Haanen JB, Larkin J, et al. Avelumab plus axitinib versus sunitinib in advanced renal cell carcinoma: Biomarker analysis of the phase 3 JAVELIN Renal 101 trial. Nat Med. 2020;26:1733–41. doi:10.1038/s41591-020-1044-8.
12. Li P, Conley A, Zhang H, Kim HL. Whole-Transcriptome profiling of formalin-fixed, paraffin-embedded renal cell carcinoma by RNA-seq. BMC Genomics. 2014;15:1087. doi:10.1186/1471-2164-15-1087.

13. Ewels P, Hammarén R, Peltzer A, Moreno D, Garcia M, Rfenouil, et al. nf-core/rnaseq: Nf-core/rnaseq version 1.4.2 2019: Zenodo. doi:10.5281/ZENODO.1400710.
14. Pertea M, Pertea GM, Antonescu CM, Chang T-C, Mendell JT, Salzberg SL. StringTie enables improved reconstruction of a transcriptome from RNA-seq reads. Nat Biotechnol. 2015;33:290–5. doi:10.1038/nbt.3122.
15. Shen-Orr SS, Gaujoux R. Computational deconvolution: Extracting cell type-specific information from heterogeneous samples. Curr. Opin. Immunol. 2013;25:571–8. doi:10.1016/j.coi.2013.09.015.
16. Avila Cobos F, Vandesompele J, Mestdagh P, Preter K de. Computational deconvolution of transcriptomics data from mixed cell populations. Bioinformatics. 2018;34:1969–79. doi:10.1093/bioinformatics/bty019.
17. Abbas AR, Wolslegel K, Seshasayee D, Modrusan Z, Clark HF. Deconvolution of blood microarray data identifies cellular activation patterns in systemic lupus erythematosus. PLoS ONE. 2009;4:e6098. doi:10.1371/journal.pone.0006098.
18. Zhong Y, Liu Z. Gene expression deconvolution in linear space. Nat. Methods. 2011;9:8-9; author reply 9. doi:10.1038/nmeth.1830.
19. Newman AM, Liu CL, Green MR, Gentles AJ, Feng W, Xu Y, et al. Robust enumeration of cell subsets from tissue expression profiles. Nat. Methods. 2015;12:453–7. doi:10.1038/nmeth.3337.
20. Holm S. A Simple Sequentially Rejective Multiple Test Procedure. Scandinavian Journal of Statistics. 1979;6:65–70.
21. Gong T, Hartmann N, Kohane IS, Brinkmann V, Staedtler F, Letzkus M, et al. Optimal deconvolution of transcriptional profiling data using quadratic programming with application to complex clinical blood samples. PLoS ONE. 2011;6:e27156. doi:10.1371/journal.pone.0027156.
22. Scherer A, editor. Batch Effects and Noise in Microarray Experiments. Chichester, UK: John Wiley & Sons, Ltd; 2009.
23. Raspollini MR, Moch H, Tan PH, Amin MB, Turajlic S. Renal cell tumors. In: WHO Classification of Tumours Editorial Board. Urinary and male genital tumours [Internet]. 2022. https://tumourclassification.iarc.who.int/chapters/36. Accessed 03 Aug 2022.
24. Chen F, Zhang Y, Şenbabaoğlu Y, Ciriello G, Yang L, Reznik E, et al. Multilevel Genomics-Based Taxonomy of Renal Cell Carcinoma. Cell Rep. 2016;14:2476–89. doi:10.1016/j.celrep.2016.02.024.
25. Shyr C, Tarailo-Graovac M, Gottlieb M, Lee JJY, van Karnebeek C, Wasserman WW. FLAGS, frequently mutated genes in public exomes. BMC Medical Genomics. 2014;7:64. doi:10.1186/s12920-014-0064-y.
26. Fu Y, Jung AW, Torne RV, Gonzalez S, Vöhringer H, Shmatko A, et al. Pan-cancer computational histopathology reveals mutations, tumor composition and prognosis. Nat Cancer. 2020;1:800–10. doi:10.1038/s43018-020-0085-8.
27. Harrell FE. Regression Modeling Strategies: With Applications to Linear Models, Logistic Regression, and Survival Analysis. 2nd ed. Cham: Springer International Publishing; 2015.
28. Dai B, Breheny P. Cross validation approaches for penalized Cox regression. arXiv:1905.10432 [stat]. 2019.

29. Royston P, Altman DG. External validation of a Cox prognostic model: Principles and methods. BMC Med Res Methodol. 2013;13:33. doi:10.1186/1471-2288-13-33.
