# Supplementary material for: A novel molecular signature identifies mixed subtypes in renal cell carcinoma with poor prognosis and independent response to immunotherapy
Source: Genome Med. 2022 Sep 15;14:105. doi: 10.1186/s13073-022-01105-y (PMC9476269; doi:10.1186/s13073-022-01105-y)
Supplement: Supplementary file 2 — Additional file 2. Supplementary Figures 1-14. [file 13073_2022_1105_MOESM2_ESM.pdf]

Fig. S1.

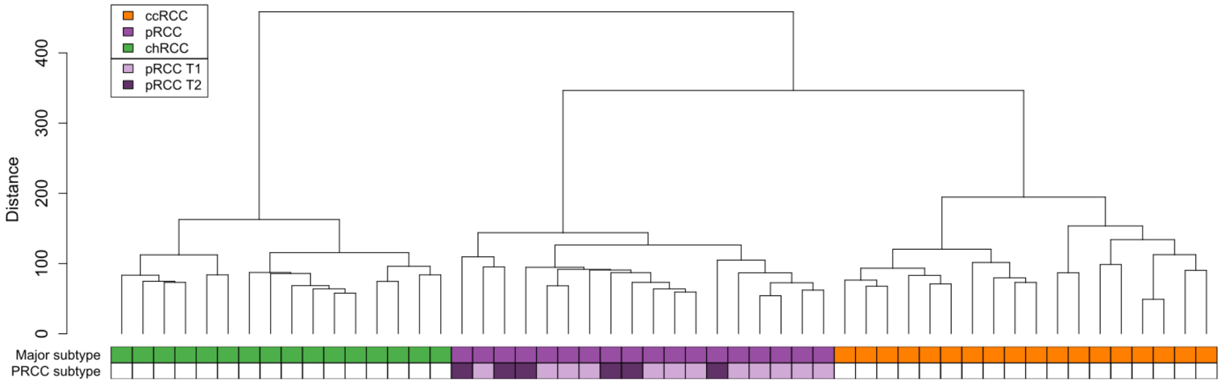

Hierarchical clustering of cohort C1. The 52 samples of cohort C1 comprised 18 ccRCC, 18 pRCC of which 12 were assigned to subtype T1 and 6 to subtype T2, and 16 chRCC. C1 was clustered using Ward's method based on genome-wide gene expression data. Expression data were sample-wise median-centered before clustering.

Fig. S2.

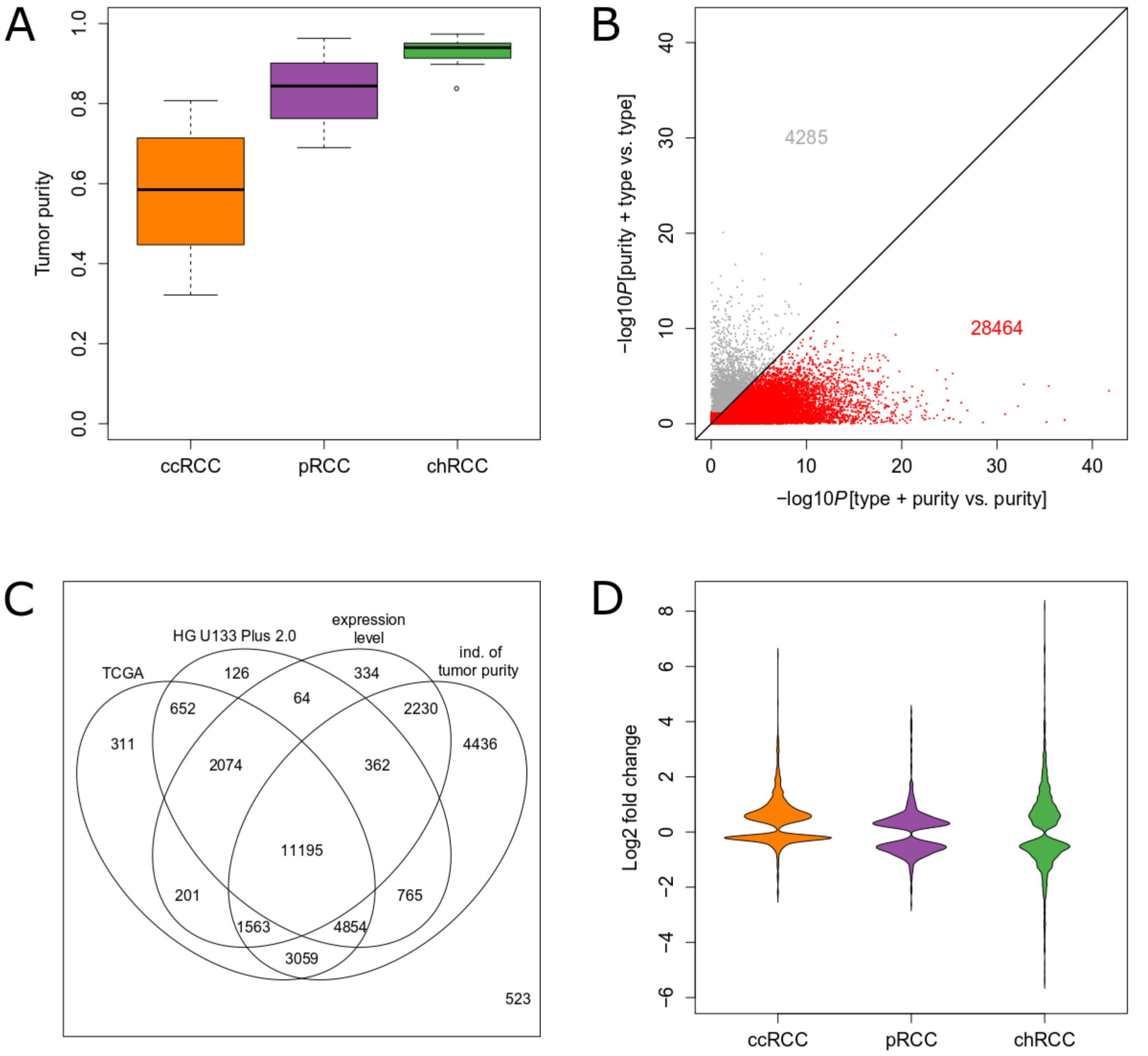

Preselection of subtype-specific genes. These analyses were performed in cohort C1 (detailed description in the corresponding section in supplementary methods). (A) Tumor purity as determined by the ESTIMATE method varies between RCC subtypes. (B) The scatter plot shows P-values obtained from model comparison for each gene. The goal of the analysis was to identify genes for which expression variability is better explained by differences in RCC subtypes than by tumor purity. 28,464 genes marked in red were stronger associated to RCC subtypes. (C) 11,195 of initially 32,749 genes (as measured by the Human Transcriptome Array 2.0 in C1) remained after four filtering

steps. "TCGA": genes covered in TCGA RNA-Seq data; "HG U133 Plus 2.0": genes covered in this microarray; "expression level": genes with median expression above the global median in C1 in minimum one subtype; "ind. of tumor purity": variance in expression was better explained by RCC subtypes than by tumor purity. (D) Subtype-specific genes (ccRCC: 1,379, pRCC: 844, chRCC: 1,463) were obtained by analysis of variance and subsequent post-hoc testing using Tukey’s method. Their expression levels were collapsed by taking the median per subtype and the minimum log2 fold change compared to the two respective other subtypes was calculated for each gene.

Fig. S3.

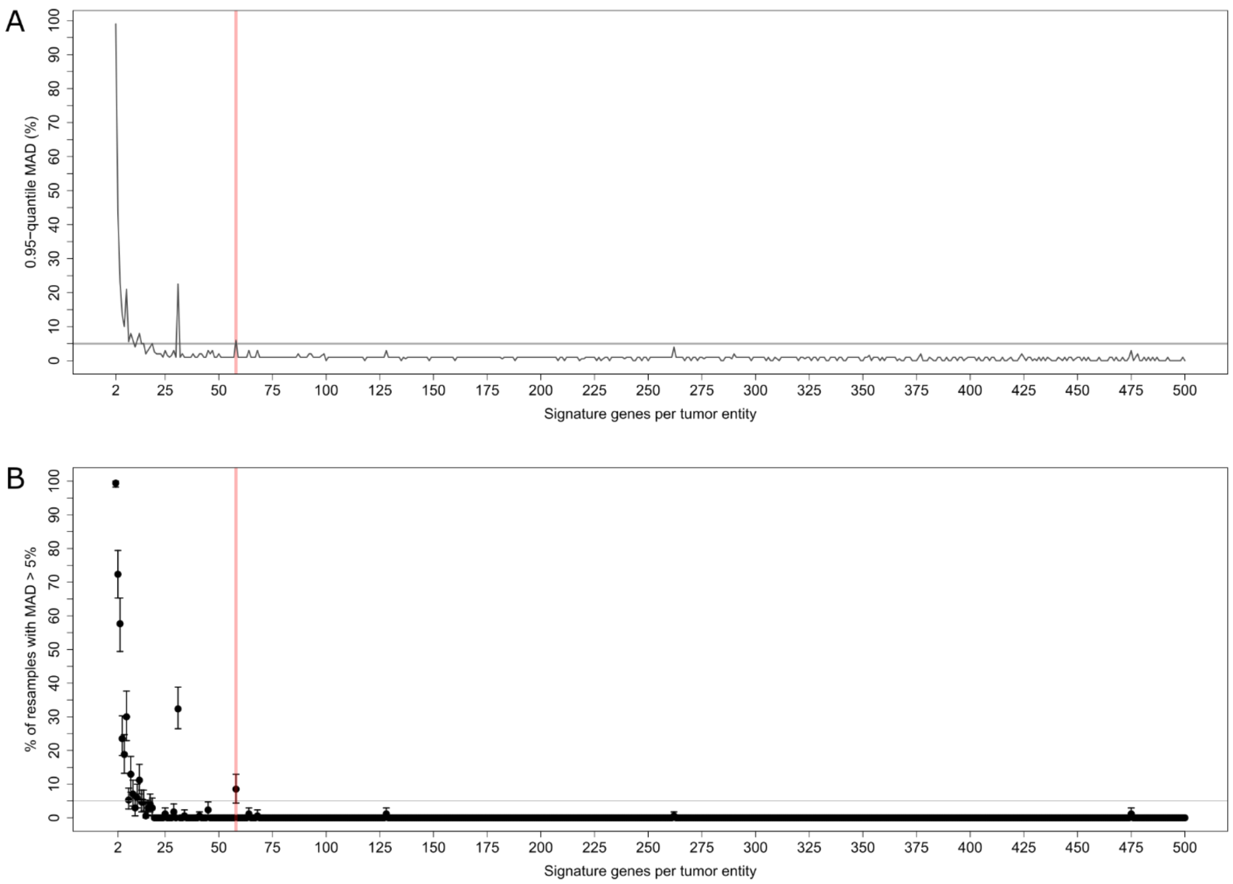

Selection of signature genes. Different $n \times 3$ signature matrices with increasing number of genes n were tested. The initial matrix included expression levels of the top two genes per subtype (i.e. $n = 6$) showing the highest log2 fold change compared to the respective other subtypes (Additional file 2. Fig. S2D). Expression levels were the median expression per subtype in C1. n was iterated from six to 500 and each matrix was used to deconvolve the 170 transcriptomes of cohort C2. The maximum absolute difference (MAD) in PSA was computed between consecutive matrices for each sample. (A) The 0.95-quantile MAD between two consecutive matrices is shown. (B) C2 was resampled 300 times with replacement and for each resample the proportion of cases experiencing a MAD > 5% compared to the respective predecessor matrix was determined. For each tested signature matrix, the median of the proportions as well as the 95% confidence interval is shown. The matrix including the top 58 genes per subtype (i.e. $n = 174$) was chosen as final signature matrix (marked in light red).

Fig. S4.

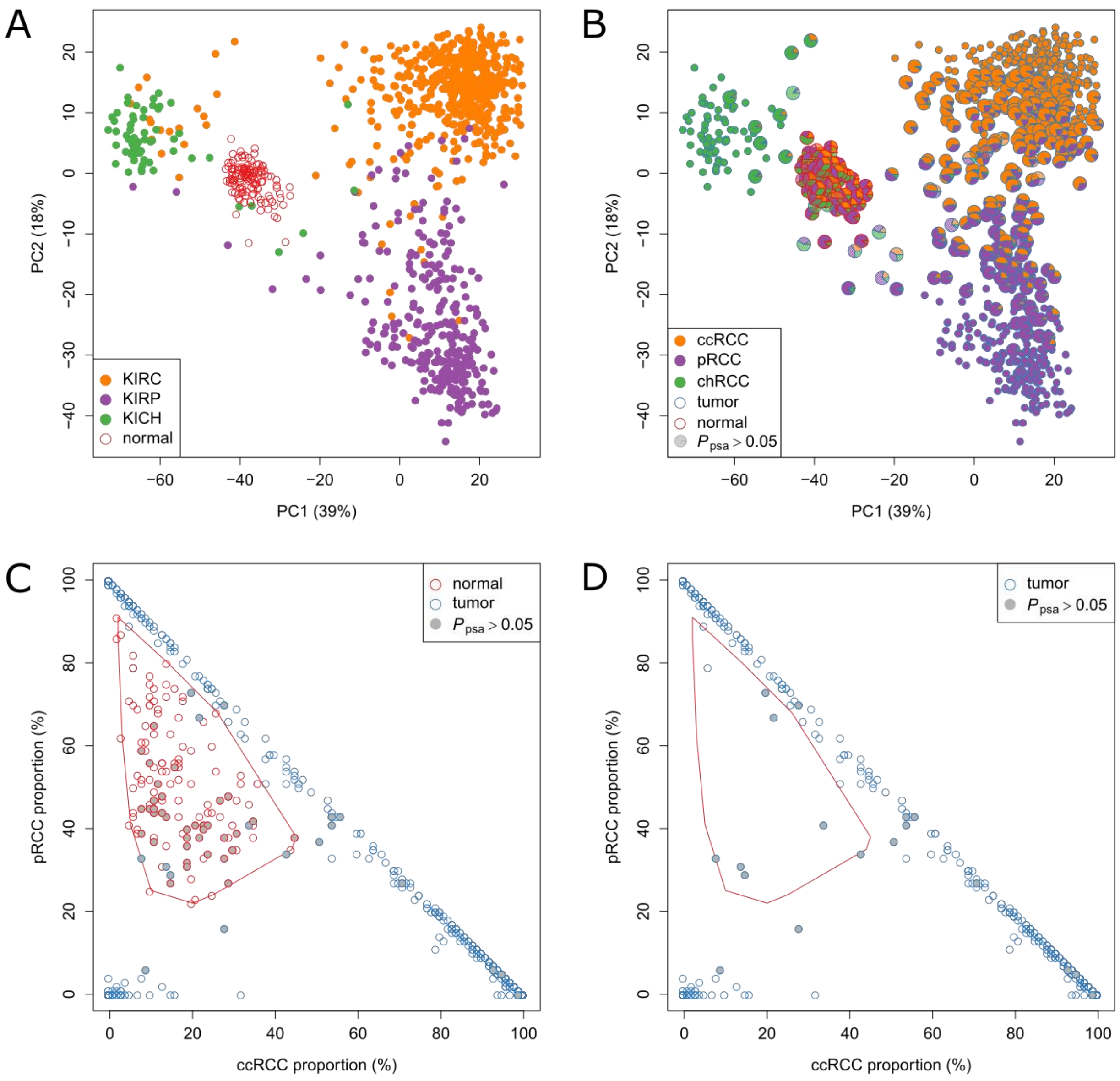

PSA of adjacent non-tumor tissue. (A) 864 tumor and 128 normal samples of the TCGA RCC cohort were analyzed together in a principal component analysis (PCA) using the set of 174 signature genes. (B) PSA were determined for 992 samples by computational deconvolution. The PSA of samples with maximum PSA value below 95% is visualized by pie charts, their symbols are also enlarged. Samples with $P_{psa} > 0.05$ as determined by a permutation P-value approach are displayed by shaded pie charts.

The sample type, i.e. whether the sample is from tumor or from normal tissue, is indicated by the color of the border. (C) Combinations of assigned proportions of ccRCC and pRCC are shown for 864 tumor and 128 normal samples. Samples with $P_{psa} > 0.05$ are indicated by a grey background. The convex hull, represented by the red line, was determined for the combinations of ccRCC and pRCC proportions assigned to normal samples. (D) Same illustration as (C), but without the normal samples.

Fig. S5.

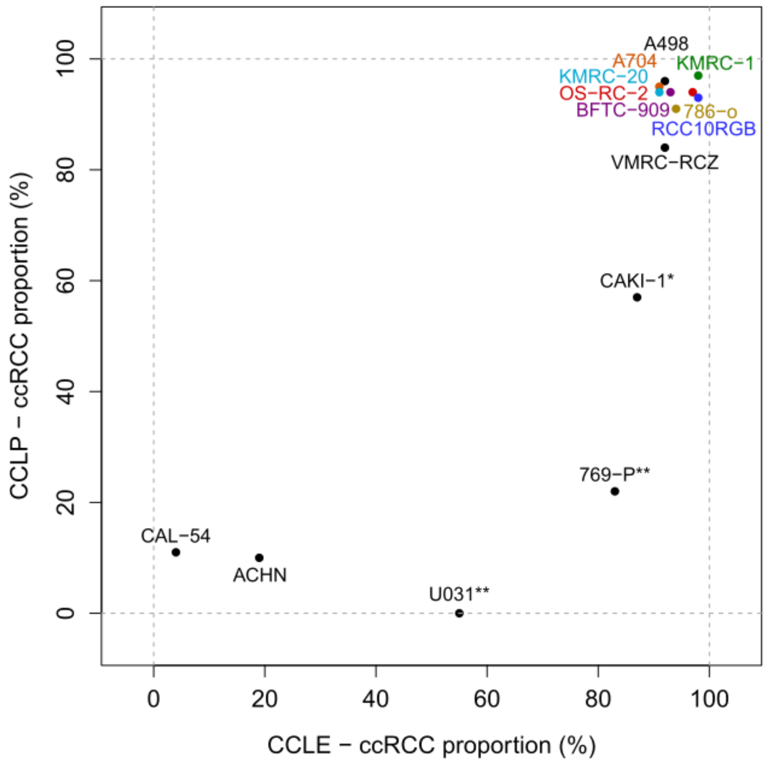

PSA of RCC cell lines. PSA for 14 RCC-derived cell lines were calculated using transcriptomic data as provided by the Broad-Novartis Cancer Cell Line Encyclopedia [1,2] (CCLE, RNA-Seq) as well as the COSMIC Cell Lines Project [3,4] (CCLP, Affymetrix Human Genome U219 Array), for details see Table S3. ccRCC proportions were sufficient to represent the PSA as chRCC proportions were consistently low in these cell lines (max. 3%). The asterisks indicate the number of PSA with $P_{psa} > 0.05$ (Additional file 5. Table S3).

Fig. S6.

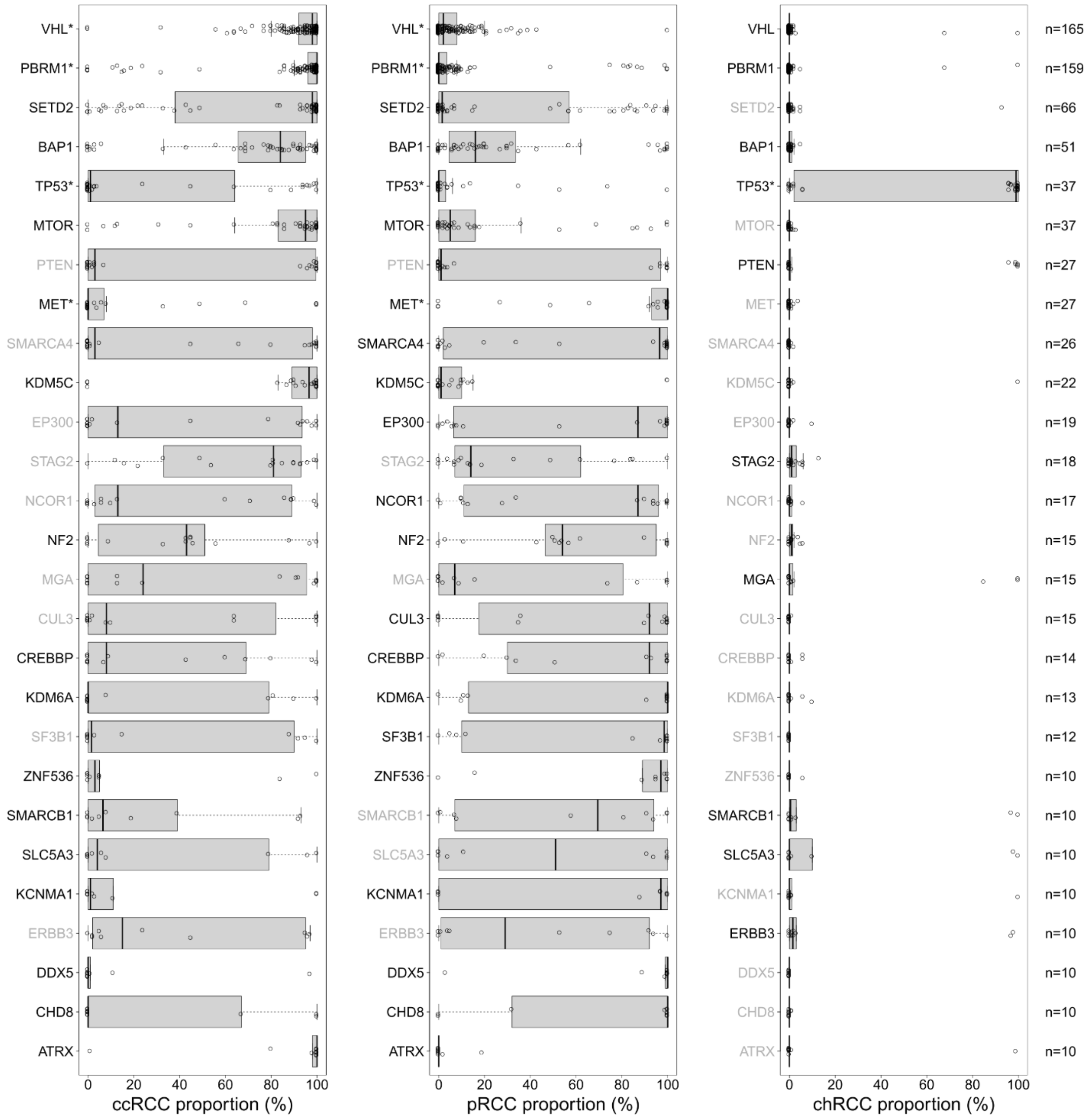

Subtype proportions of carriers of somatic mutations. PSA were correlated with the occurrence of somatic mutations in 310 frequently mutated genes in RCC subtypes. Mutations were found in 620 tumors of C3. For each gene and subtype, the distribution of assigned proportions was compared between tumors with and without a somatic mutation using the Cramer-von Mises test. Here, PSA of somatic mutation carriers are

shown for 27 genes, which were significant in minimum one RCC subtype (without correction for multiple testing) and additionally were mutated in minimum 10 tumors. On the right, the number of tumors from the 620 tumors with a somatic mutation in the respective gene is shown. Greyed out gene names indicate a P-value > 0.05 and star symbols point to significance after P-value adjustment. Boxes refer to median subtype scores and interquartile ranges with whiskers extending to a maximum of 1.5 times the interquartile range. Tumors with overlapping molecular features of RCC subtypes based on PSA (max. PSA < 95%) were significantly enriched for somatic mutations in *BAP1* (P=0.0010, Fisher-test) and *STAG2* (P=0.0056, Fisher-test).

Fig. S7.

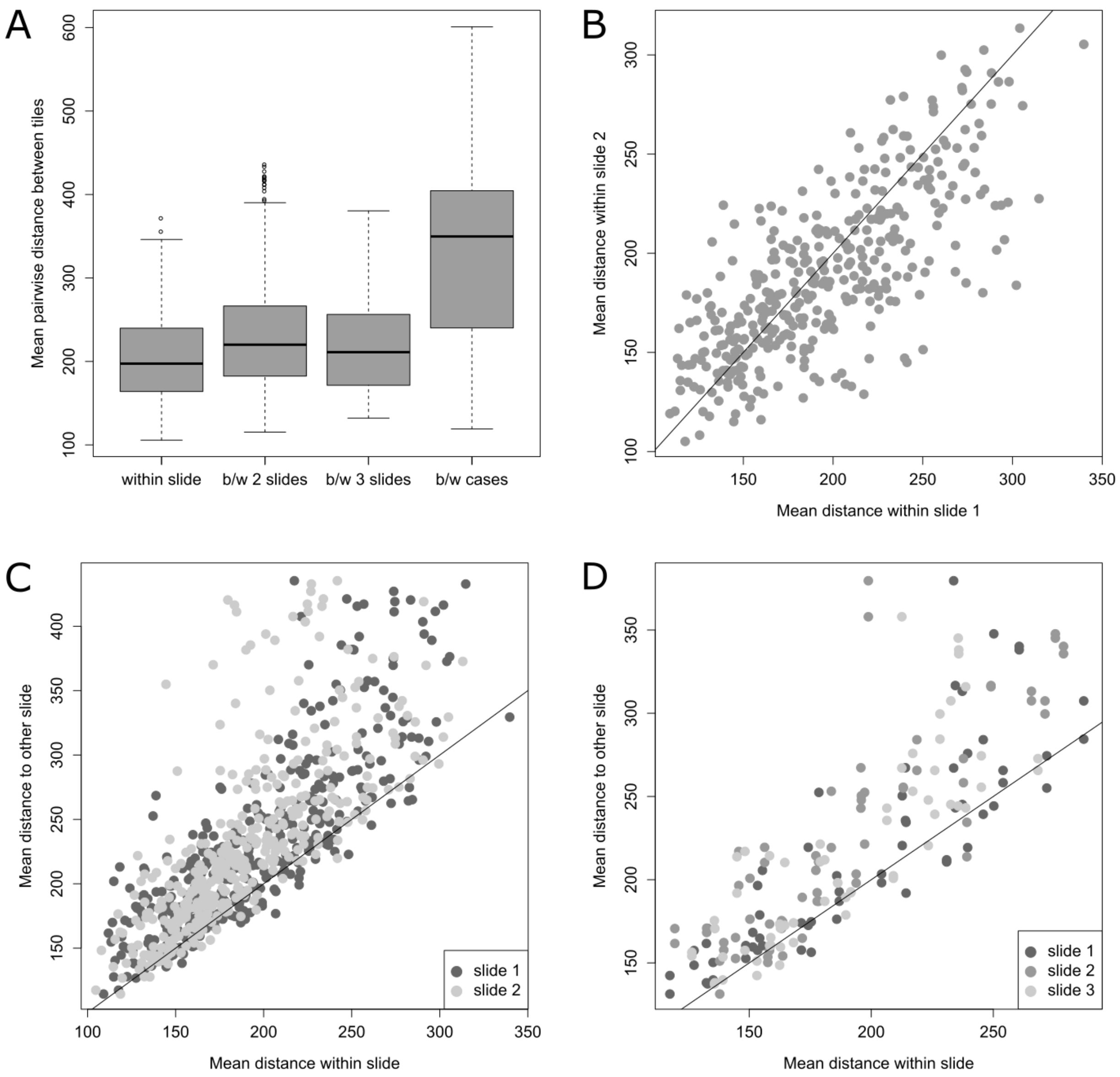

Distance measure based on computational histopathological features. Distances between 1,084 slides from 396 KIRC (729 slides), 187 KIRP (237) and 65 KICH (118) tumors were determined. The Manhattan distance for a pair of tiles was calculated using 1,536 histopathological features that were learned by a convolutional neural network [5]. The mean pairwise distance between tiles of one slide was referred to as “within slide” distance. The mean distances of all tile combinations between two slides was used as the distance between (b/w) them. (A) The distribution of 1,084 “within slide” distances

was compared with distances between related slides (from the same tumor) and unrelated slides (from different cases). Two slides were available for 374 cases and 3 slides for 93 cases. 586,519 distances between different tumors are shown. (B) For cases with two slides available, the “within slide” distances of slides from the same tumor are compared. For cases with two slides (C) or three slides (D) available, the “within slide” distances are compared to the corresponding “b/w slides” distances.

Fig. S8.

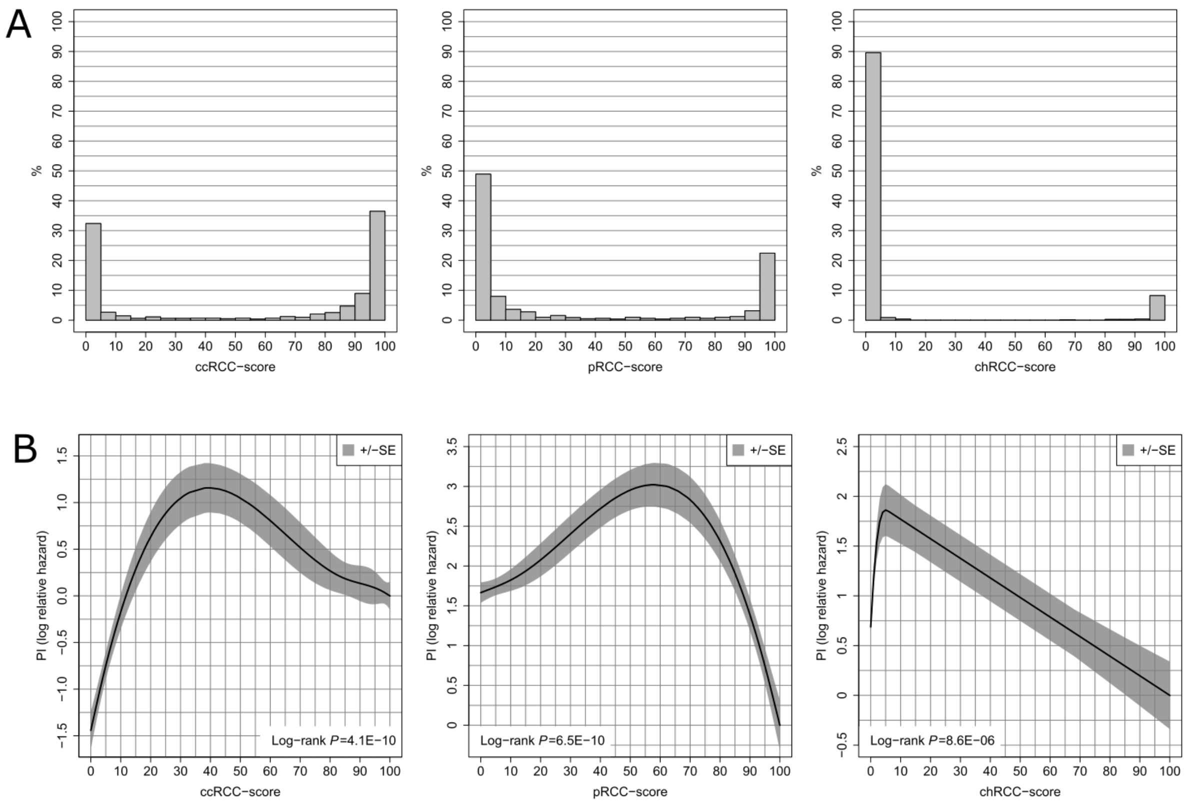

Subtype scores. 828 patients of C3 with available PSA ($P_{psa} < 0.05$) and survival data were used in these analyses. (A) Histograms showing the relative frequency of assigned proportions for each subtype score. (B) Restricted cubic spline estimates of the relationships between subtype scores and log relative hazards (here used as prognostic index (PI)) based on a Cox PH model with endpoint CSS are shown. Pointwise standard errors are displayed. 5, 4, and 3 knots were used for fitting ccRCC-score, pRCC-score, and chRCC-score to log relative hazards, respectively. The curves were shifted in such a way that patients whose tumor was assigned a score value of 100 had a PI value of zero, respectively.

Fig. S9.

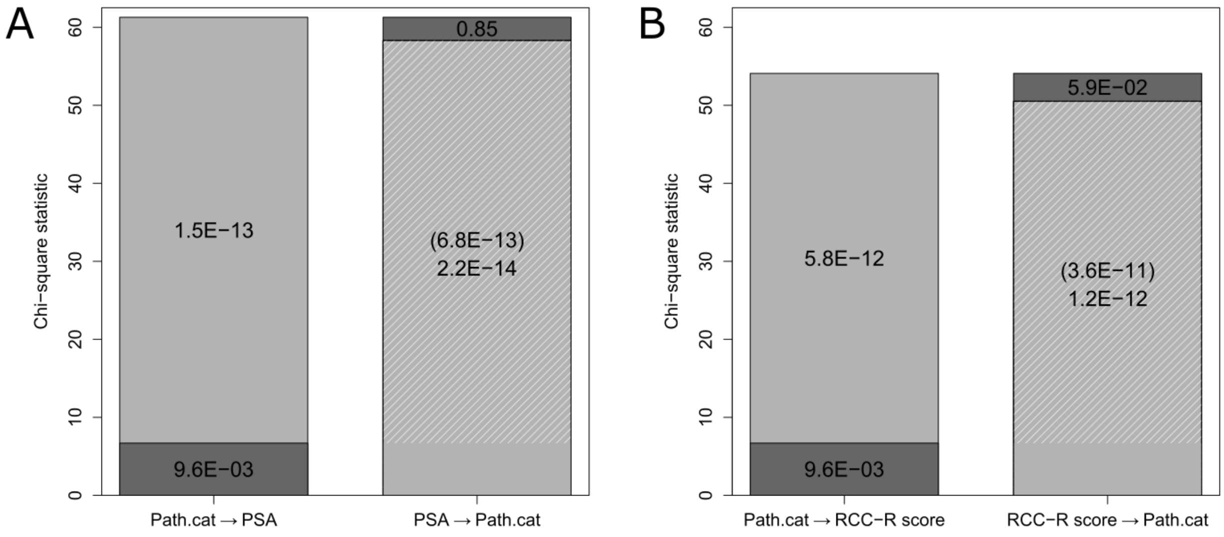

Model comparison by analysis of deviance. Models were compared using 789 patients of C3 with available PSA ($P_{psa} < 0.05$), survival data, and pathological classification (Path.cat: ccRCC (n=457), pRCC (n=261), and chRCC (n=71)). Chi-square statistic values show the different contributions of PSA or RCC-R score and pathological classification to the model likelihood. Chi-squared test P-values are shown in the bars. (A) Left: PSA (additive combination of ccRCC- and pRCC-score, both modeled via restricted cubic splines) represented by the prognostic index (PI) was added as predictor to the Cox model initially including only Path.cat as predictor. Right: Path.cat was used to complement the PI. The shaded area highlights the additional contribution to model likelihood by PSA in comparison to Path.cat, with the corresponding P-value in brackets. (B) Analogous to (A) with PSA replaced by the RCC-R score. In contrast to PSA in (A), which refers here to the use of both the ccRCC- and the pRCC-score as predictors, the RCC-R score involves only the ccRCC-score (see equation 1).

Fig. S10.

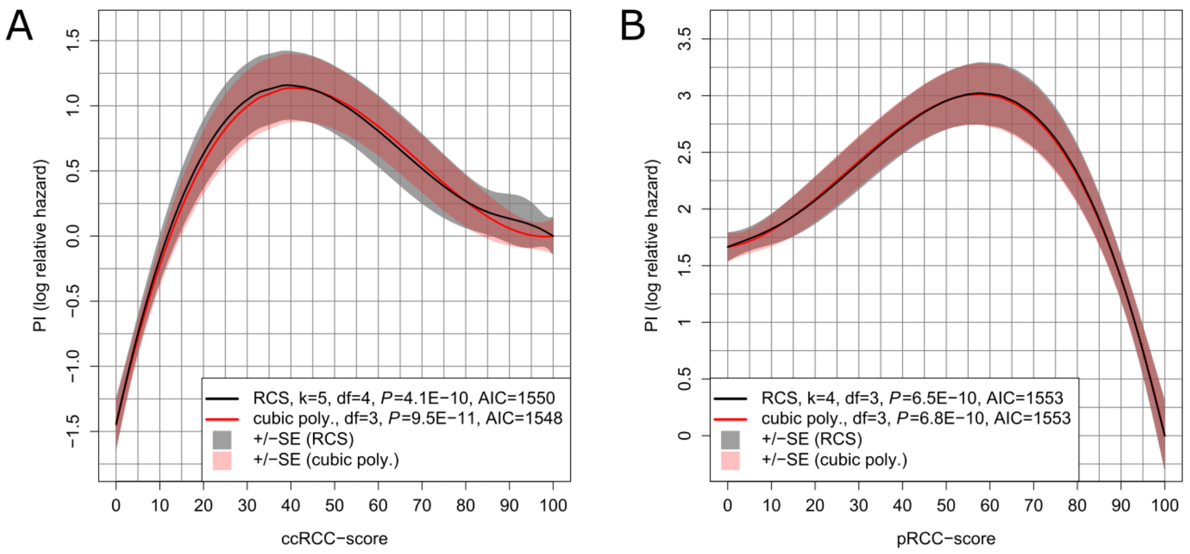

Relationship between subtype scores and log relative hazards. 828 patients of C3 with available PSA ($P_{psa} < 0.05$) and survival data were used in these analyses. PSA were modeled via restricted cubic spline (RCS) functions or cubic polynomials. Pointwise standard errors are displayed. The number of knots (k) as well as the degree of freedoms (df) are shown together with the log-rank test P-value resulting from Cox regression analysis and the Akaike information criterion (AIC, see statistical tools) values. (A) ccRCC-score. (B) pRCC-score.

Fig. S11.

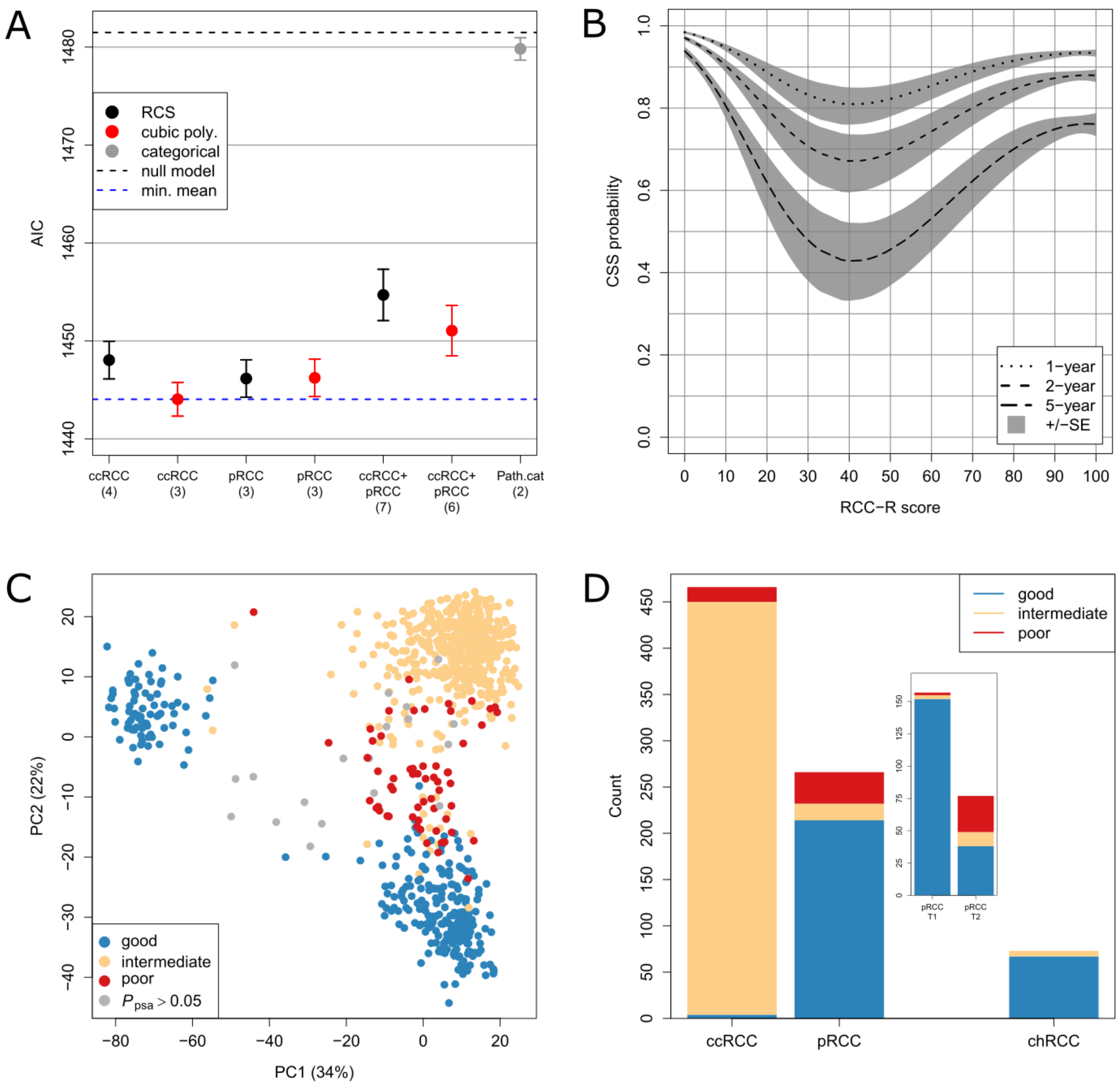

RCC-R score. (A) Predictive ability of subtype scores and pathological classifications was evaluated by repeated 10-fold cross validation using 786 patients of C3. Metabolically divergent (MD) chRCC cases were not included in this analysis. The Akaike information criterion (AIC) estimates the relative amount of information lost by a given model (see statistical tools). Points and error bars indicate mean +/- standard deviation of AIC based on 300 iterations. Both the minimum AIC mean value achieved

by a predictor and the AIC value of the null model are marked by dashed lines. The number of degrees of freedom of the Cox models is given in the brackets, respectively. Black: predictor(s) modeled via restricted cubic splines, red: predictor(s) modeled via cubic polynomials, grey: categorical predictor. Path.cat: ccRCC (n=457), pRCC (n=261), and chRCC (n=68). The ccRCC-score modeled via cubic polynomials achieved the minimum mean AIC value and is hereafter called RCC-R score. (B) Predicted CSS rates (1-, 2-, 5-year) in dependence on the RCC-R score (i.e. ccRCC proportions) were calculated for C3 using the prognostic index together with its pointwise standard error and the baseline survival function from the RCC-R score Cox model evaluated in cohort C3 (n=828). (C) Risk groups were defined based on the RCC-R score, for cutoffs see figure 5D. Samples of C3 were colored according to their predicted risk group. (D) Barplot showing the relationship between RCC-R score-related risk groups and pathological classification in C3 (n=805). Samples with $P_{psa} > 0.05$ were not considered here.

Fig. S12.

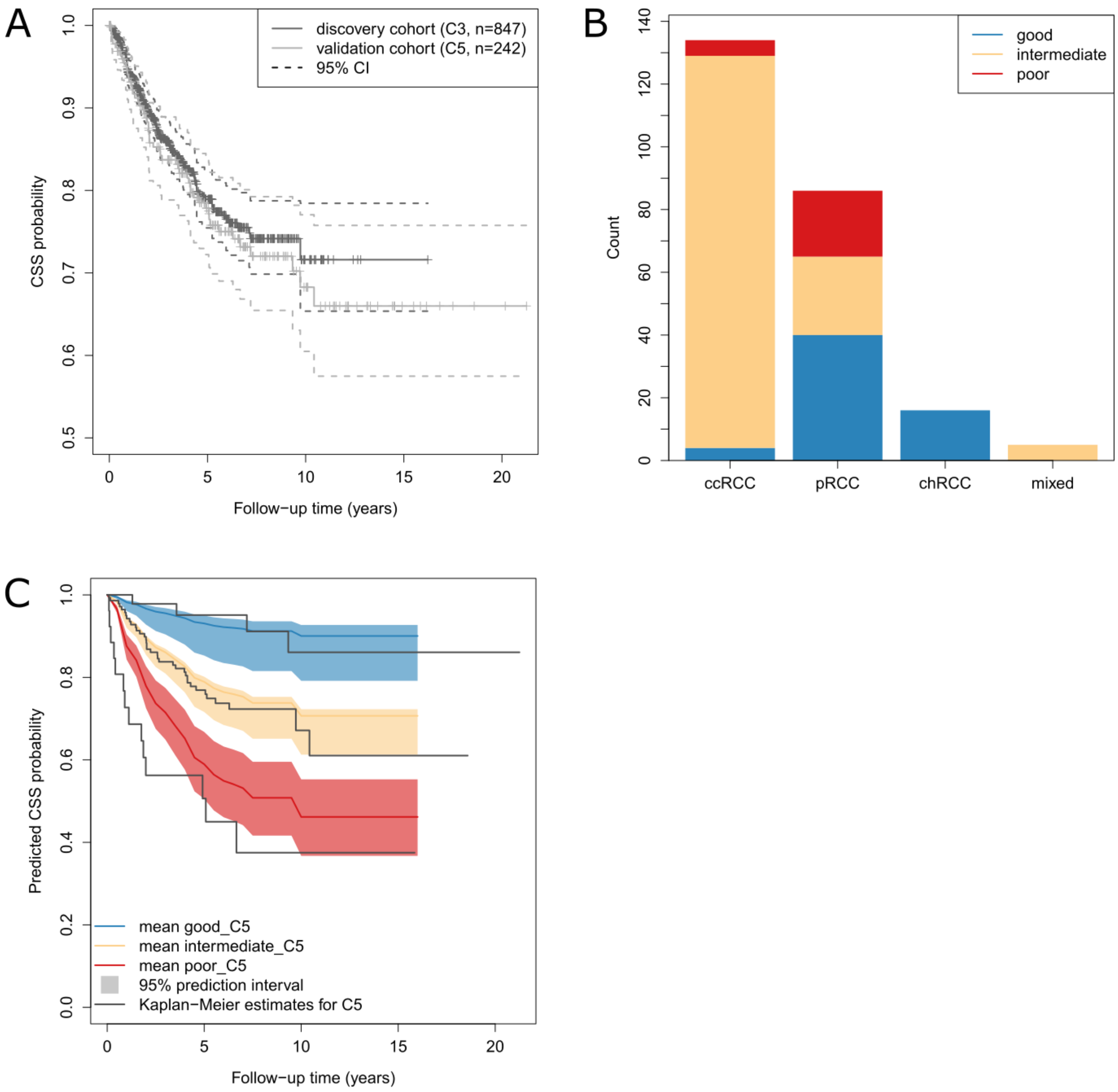

External validation of the RCC-R score. (A) Kaplan-Meier estimates together with 95% confidence interval of the discovery (C3, n=847) and the validation cohort (C5, n=242) are shown. (B) Barplot showing the relationship between RCC-R score-related risk groups and original pathological classification in C5 using 241 patients with available PSA ($P_{psa} < 0.05$). (C) The prognostic index, PI_{c5}, was calculated for 241 patients of cohort C5 using equation 1. Their CSS probabilities were then predicted at half-yearly intervals from 0 to 16 years using the baseline survival function from the

RCC-R score Cox model evaluated in the discovery cohort C3. Five patients of C5 whose follow-up time exceeded the maximum follow-up time in C3 were not used for the CSS prediction. The mean of the predicted probabilities along with the 95% prediction interval are displayed for each time point and risk group. For comparison the Kaplan-Meier estimates for CSS in the three risk groups in C5 are added.

Fig. S13.

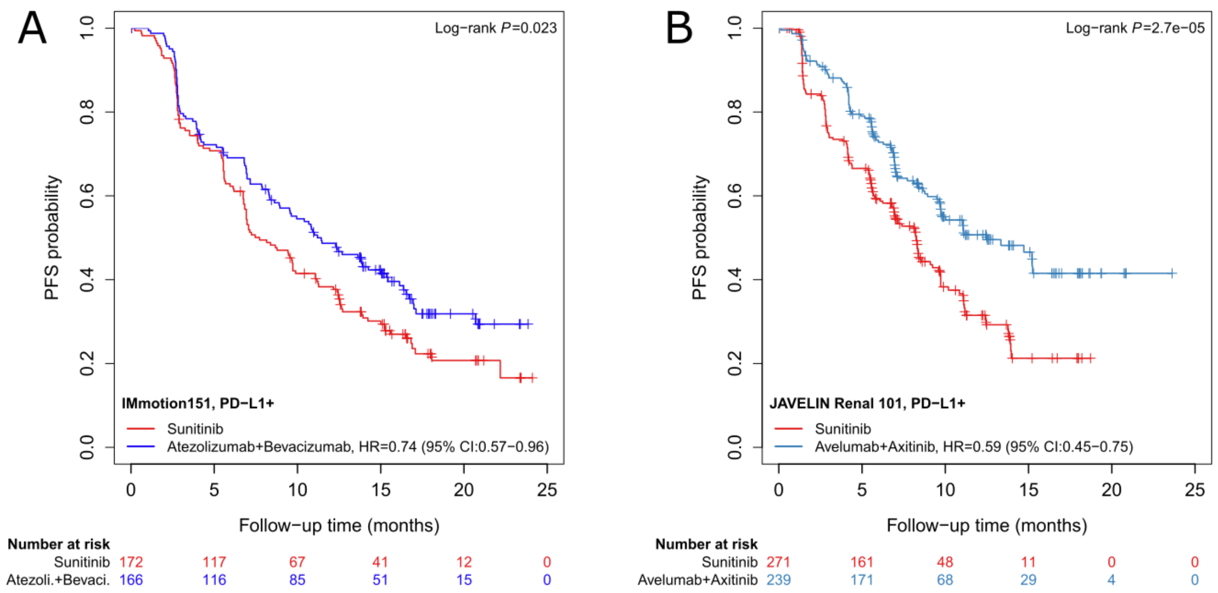

PFS of PD-L1-positive tumors treated with angiogenesis and/or checkpoint inhibition. Kaplan-Meier estimates for progression-free survival (PFS) in PD-L1-positive tumors are presented for both the IMmotion151 trial (n=338) [6] (A) and the JAVELIN Renal 101 trial (n=510) [7] (B). In both trials, a combination therapy including anti-angiogenic treatment as well as immunocheckpoint inhibition was compared against sunitinib monotherapy in advanced renal cell carcinoma. Patients with non-significant PSA ($P_{psa} > 0.05$) based on whole transcriptome analysis were not included here. Cox regression analysis was used to determine the log-rank test P-values as well as the hazard ratios (HR).

Fig. S14.

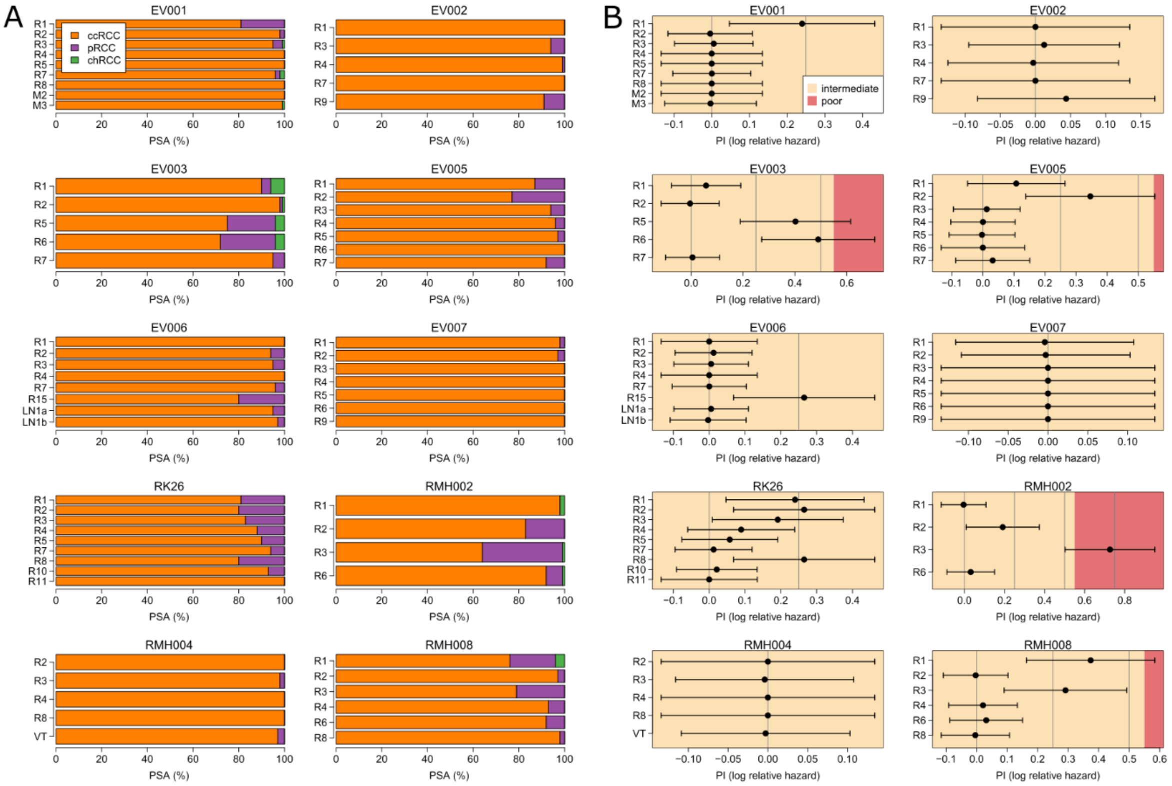

Intratumor heterogeneity of PSA and RCC-R score. Published gene expression data of 65 distinct regions derived from 10 ccRCC [8,9] were obtained from Gene Expression Omnibus (GSE31610, GSE53000). Expression data were generated with Affymetrix HuGene-1_0-st-v1. Microarrays were normalized individually using the SCAN method from the R-package SCAN.UPC and probe sets were summarized on Entrez GeneID level using the annotation provided by brainarray [10,11] (version 23). (A) 172 of 174 signatures genes were available for the computation of the PSA. (B) Points display the prognostic index (PI) (see equation 1) together with +/- one standard error based on the RCC-R score for each tumor region. PI cutoffs for risk group assignment based on the RCC-R score (see Fig. 5D) are indicated by the background color.

References

1. Broad Institute and Novartis Institutes for Biomedical Research. Broad-Novartis Cancer Cell Line Encyclopedia. www.broadinstitute.org/ccle.
2. Ghandi M, Huang FW, Jané-Valbuena J, Kryukov GV, Lo CC, McDonald ER, et al. Next-generation characterization of the Cancer Cell Line Encyclopedia. Nature. 2019;569:503–8. doi:10.1038/s41586-019-1186-3.
3. COSMIC Cell Lines project. http://cancer.sanger.ac.uk/cell_lines.
4. Tate JG, Bamford S, Jubb HC, Sondka Z, Beare DM, Bindal N, et al. COSMIC: The Catalogue Of Somatic Mutations In Cancer. Nucleic Acids Res. 2019;47:D941-D947. doi:10.1093/nar/gky1015.Broad Institute and Novartis Institutes for Biomedical Research. Broad-Novartis Cancer Cell Line Encyclopedia. www.broadinstitute.org/ccle.
5. Fu Y, Jung AW, Torne RV, Gonzalez S, Vöhringer H, Shmatko A, et al. Pan-cancer computational histopathology reveals mutations, tumor composition and prognosis. Nat Cancer. 2020;1:800–10. doi:10.1038/s43018-020-0085-8.
6. Motzer RJ, Banchereau R, Hamidi H, Powles T, McDermott D, Atkins MB, et al. Molecular Subsets in Renal Cancer Determine Outcome to Checkpoint and Angiogenesis Blockade. Cancer Cell. 2020;38:803-817.e4. doi:10.1016/j.ccell.2020.10.011.
7. Motzer RJ, Robbins PB, Powles T, Albiges L, Haanen JB, Larkin J, et al. Avelumab plus axitinib versus sunitinib in advanced renal cell carcinoma: Biomarker analysis of the phase 3 JAVELIN Renal 101 trial. Nat Med. 2020;26:1733–41. doi:10.1038/s41591-020-1044-8.
8. Gerlinger M, Horswell S, Larkin J, Rowan AJ, Salm MP, Varela I, et al. Genomic architecture and evolution of clear cell renal cell carcinomas defined by multiregion sequencing. Nat. Genet. 2014;46:225–33. doi:10.1038/ng.2891.
9. Gerlinger M, Rowan AJ, Horswell S, Math M, Larkin J, Endesfelder D, et al. Intratumor heterogeneity and branched evolution revealed by multiregion sequencing. N. Engl. J. Med. 2012;366:883–92. doi:10.1056/NEJMoa1113205.
10. Dai M, Wang P, Boyd AD, Kostov G, Athey B, Jones EG, et al. Evolving gene/transcript definitions significantly alter the interpretation of GeneChip data. Nucleic Acids Res. 2005;33:e175. doi:10.1093/nar/gni179.
11. Brainarray. http://brainarray.mbni.med.umich.edu.
