# Supplementary material for: A novel molecular signature identifies mixed subtypes in renal cell carcinoma with poor prognosis and independent response to immunotherapy
Source: Genome Med. 2022 Sep 15;14:105. doi: 10.1186/s13073-022-01105-y (PMC9476269; doi:10.1186/s13073-022-01105-y)
Supplement: Supplementary file 3 — Additional file 3: Table S1. Patient characteristics of cohort C1. [file 13073_2022_1105_MOESM3_ESM.pdf]

Table S1. Patient characteristics of cohort C1.

Characteristic	C1 (n=52)	
	n	%
**Sex**		
Male	35	67.3
Female	17	32.7
**T**		
1	30	57.7
2	6	11.5
3	16	30.8
4	0	0.0
**N**		
0	50	96.2
1/2	1	1.9
X	1	1.9
**M**		
0	46	88.5
1	5	9.6
X	1	1.9
**Histology**		
ccRCC	18	34.6
pRCC	18	34.6
chRCC	16	30.8
**Age, years**		
Median	63	
Range	22 to 87	
**Tumor size, cm**		
Median	5	
Range	1.7 - 16	
