# Supplementary material for: A novel molecular signature identifies mixed subtypes in renal cell carcinoma with poor prognosis and independent response to immunotherapy
Source: Genome Med. 2022 Sep 15;14:105. doi: 10.1186/s13073-022-01105-y (PMC9476269; doi:10.1186/s13073-022-01105-y)
Supplement: Supplementary file 4 — Additional file 4: Table S2. Composition of cohort C2. [file 13073_2022_1105_MOESM4_ESM.pdf]

Table S2. Composition of cohort C2. C2 (n=170) includes RCC samples from seven studies providing gene expression data on Gene Expression Omnibus and 12 RCC samples with expression data generated within the present project. If available, the number of type 1 and type 2 cases is reported for pRCC.

Study	GEO accession	ccRCC	pRCC	chRCC
Yusenko et al., 2009 [1]	GSE11151	26	19	4
Tan et al., 2010 [2]	GSE19982	0	0	15
Peña-Llopis et al., 2012 [3]	GSE36895	29	0	0
Furge et al., 2007 [4]	GSE7023	0	T1: 22, T2: 13	0
Ho et al., 2017 [5]	GSE85258	14	1	0
Koeman et al., 2008 [6]	GSE8271	0	0	10
Lang et al., 2016 [7]	GSE83820	5	0	0
present study		0	T1: 6, T2: 6	0

References

1. Yusenko MV, Kuiper RP, Boethe T, Ljungberg B, van Kessel AG, Kovacs G. High-resolution DNA copy number and gene expression analyses distinguish chromophobe renal cell carcinomas and renal oncocytomas. BMC Cancer. 2009;9:152. doi:10.1186/1471-2407-9-152.
2. Tan M-H, Wong CF, Tan HL, Yang XJ, Ditlev J, Matsuda D, et al. Genomic expression and single-nucleotide polymorphism profiling discriminates chromophobe renal cell carcinoma and oncocytoma. BMC Cancer. 2010;10:196. doi:10.1186/1471-2407-10-196.
3. Peña-Llopis S, Vega-Rubín-de-Celis S, Liao A, Leng N, Pavía-Jiménez A, Wang S, et al. BAP1 loss defines a new class of renal cell carcinoma. Nat. Genet. 2012;44:751–9. doi:10.1038/ng.2323.
4. Furge KA, Chen J, Koeman J, Swiatek P, Dykema K, Lucin K, et al. Detection of DNA copy number changes and oncogenic signaling abnormalities from gene expression data reveals MYC activation in high-grade papillary renal cell carcinoma. Cancer Res. 2007;67:3171–6. doi:10.1158/0008-5472.CAN-06-4571.
5. Ho TH, Serie DJ, Parasramka M, Cheville JC, Bot BM, Tan W, et al. Differential gene expression profiling of matched primary renal cell carcinoma and metastases reveals upregulation of extracellular matrix genes. Ann. Oncol. 2017;28:604–10. doi:10.1093/annonc/mdw652.
6. Koeman JM, Russell RC, Tan M-H, Petillo D, Westphal M, Koelzer K, et al. Somatic pairing of chromosome 19 in renal oncocytoma is associated with deregulated EGLN2-mediated corrected oxygen-sensing response. PLoS Genet. 2008;4:e1000176. doi:10.1371/journal.pgen.1000176.
7. Lang H, Béraud C, Bethry A, Danilin S, Lindner V, Coquard C, et al. Establishment of a large panel of patient-derived preclinical models of human renal cell carcinoma. Oncotarget. 2016;7:59336–59. doi:10.18632/oncotarget.10659.
