# Supplementary material for: A novel molecular signature identifies mixed subtypes in renal cell carcinoma with poor prognosis and independent response to immunotherapy
Source: Genome Med. 2022 Sep 15;14:105. doi: 10.1186/s13073-022-01105-y (PMC9476269; doi:10.1186/s13073-022-01105-y)
Supplement: Supplementary file 5 — Additional file 5: Table S3. PSA of RCC cell lines. [file 13073_2022_1105_MOESM5_ESM.pdf]

Table S3. PSA of RCC cell lines. PSA for 14 RCC-derived cell lines were calculated using transcriptomic data as provided by the Broad-Novartis Cancer Cell Line Encyclopedia [1,2] (CCLE, RNA-Seq) as well as the COSMIC Cell Lines Project [3,4] (CCLP, Affymetrix Human Genome U219 Array). RPKM values from CCLE (CCLE_RNAseq_genes_rpkm_20180929.gct.gz) were used for computation of PSA. Ensembl gene identifiers were mapped to Entrez GeneIDs using the annotation provided by the org.Hs.eg.db R-package. Microarray data from CCLP were downloaded from ArrayExpress (accession code: E-MTAB-3610). CCLP cell lines were normalized individually using the SCAN method from the R-package SCAN.UPC. Probe sets were summarized on Entrez GeneID level using the annotation provided by brainarray [5,6] (version 23). Three (*SLC2A3*, *UGT1A6*, *BNIP3*) of the 174 signature genes were not covered in the brainarray annotation of this microarray.

	**CCLE**				**CCLP**			
**Cell line**	**ccRCC**	**pRCC**	**chRCC**	P_{psa}	**ccRCC**	**pRCC**	**chRCC**	P_{psa}
769-P	83	15	2	0.0783	22	78	0	0.0861
786-o	94	3	3	0.0330	91	6	3	0.0002
A498	92	7	1	0.0001	96	3	1	0.0001
A704	91	8	1	0.0009	95	2	3	0.0001
ACHN	19	81	0	0.0263	10	90	0	0.0010
BFTC-909	93	5	2	0.0228	94	6	0	0.0045
CAKI-1	87	11	2	0.6291	57	42	1	0.0013
CAL-54	4	95	1	0.0023	11	89	0	0.0016
KMRC-1	98	0	2	0.0001	97	3	0	0.0001
KMRC-20	91	6	3	0.0001	94	4	2	0.0005
OS-RC-2	97	3	0	0.0001	94	6	0	0.0001
RCC10RGB	98	2	0	0.0008	93	5	2	0.0001
UO31	55	45	0	0.2047	0	100	0	0.0572
VMRC-RCZ	92	5	3	0.0043	84	16	0	0.0013

References

1. Broad Institute and Novartis Institutes for Biomedical Research. Broad-Novartis Cancer Cell Line Encyclopedia. www.broadinstitute.org/ccle.
2. Ghandi M, Huang FW, Jané-Valbuena J, Kryukov GV, Lo CC, McDonald ER, et al. Next-generation characterization of the Cancer Cell Line Encyclopedia. Nature. 2019;569:503–8. doi:10.1038/s41586-019-1186-3.
3. COSMIC Cell Lines project. http://cancer.sanger.ac.uk/cell_lines.
4. Tate JG, Bamford S, Jubb HC, Sondka Z, Beare DM, Bindal N, et al. COSMIC: The Catalogue Of Somatic Mutations In Cancer. Nucleic Acids Res. 2019;47:D941-D947. doi:10.1093/nar/gky1015.
5. Dai M, Wang P, Boyd AD, Kostov G, Athey B, Jones EG, et al. Evolving gene/transcript definitions significantly alter the interpretation of GeneChip data. Nucleic Acids Res. 2005;33:e175. doi:10.1093/nar/gni179.
6. Brainarray. http://brainarray.mbni.med.umich.edu.
