# Supplementary material for: A novel molecular signature identifies mixed subtypes in renal cell carcinoma with poor prognosis and independent response to immunotherapy
Source: Genome Med. 2022 Sep 15;14:105. doi: 10.1186/s13073-022-01105-y (PMC9476269; doi:10.1186/s13073-022-01105-y)
Supplement: Supplementary file 6 — Additional file 6: Table S4. Signature genes. [file 13073_2022_1105_MOESM6_ESM.pdf]

Table S4. Signature genes. The 58 subtype-specific genes per RCC subtype are ordered by decreasing log2 fold change. Associated Hallmark gene sets provided by MSigDB (https://www.gsea-msigdb.org/gsea/msigdb/, version 7.1) are displayed**.**

**ccRCC**	Hallmark gene sets (MSigDB 7.1)
CP	COMPLEMENT, HYPOXIA
LOX	EPITHELIAL_MESENCHYMAL_TRANSITION, HYPOXIA
EGLN3	GLYCOLYSIS, MTORC1_SIGNALING
CYP2J2	XENOBIOTIC_METABOLISM
CDR1	
NDUFA4L2	
ANGPTL4	ADIPOGENESIS, GLYCOLYSIS, HYPOXIA, KRAS_SIGNALING_UP
SLC5A12	
FRZB	
GSTA1	
DNAH11	
SLC17A4	
PLN	
PLCB1	PI3K_AKT_MTOR_SIGNALING
HILPDA	
ABCC2	XENOBIOTIC_METABOLISM
A1CF	
SERPINE1	COAGULATION, COMPLEMENT, EPITHELIAL_MESENCHYMAL_TRANSITION, HYPOXIA, INFLAMMATORY_RESPONSE, TGF_BETA_SIGNALING, TNFA_SIGNALING_VIA_NFKB, UV_RESPONSE_DN, XENOBIOTIC_METABOLISM
SLC2A3	HYPOXIA, IL2_STAT5_SIGNALING, MTORC1_SIGNALING, TNFA_SIGNALING_VIA_NFKB
GBP4	IL2_STAT5_SIGNALING, INTERFERON_ALPHA_RESPONSE, INTERFERON_GAMMA_RESPONSE
MME	
F8	COAGULATION, COMPLEMENT
FMO2	
CALCRL	INFLAMMATORY_RESPONSE
UGT1A6	
KMO	
FHL5	
ST8SIA4	ALLOGRAFT_REJECTION, INTERFERON_GAMMA_RESPONSE
SLC2A1	ESTROGEN_RESPONSE_EARLY, HEME_METABOLISM, HYPOXIA, MTORC1_SIGNALING, PI3K_AKT_MTOR_SIGNALING
PREX2	
IGFBP3	EPITHELIAL_MESENCHYMAL_TRANSITION, GLYCOLYSIS, HYPOXIA, KRAS_SIGNALING_UP, MYOGENESIS
ALDOC	CHOLESTEROL_HOMEOSTASIS, HYPOXIA
PDK4	OXIDATIVE_PHOSPHORYLATION, XENOBIOTIC_METABOLISM
CDH11	APICAL_JUNCTION, EPITHELIAL_MESENCHYMAL_TRANSITION

INHBB	ALLOGRAFT_REJECTION, ESTROGEN_RESPONSE_EARLY, P53_PATHWAY
EDN1	INFLAMMATORY_RESPONSE, KRAS_SIGNALING_DN, TNFA_SIGNALING_VIA_NFKB
TMEM45A	HYPOXIA
SLC6A3	KRAS_SIGNALING_DN
GIMAP7	
IFI44L	INTERFERON_ALPHA_RESPONSE, INTERFERON_GAMMA_RESPONSE, KRAS_SIGNALING_DN
HEG1	
P4HA1	GLYCOLYSIS, HYPOXIA, IL2_STAT5_SIGNALING, MTORC1_SIGNALING
GIMAP4	
ETS1	ALLOGRAFT_REJECTION, HYPOXIA, KRAS_SIGNALING_UP
LHFPL6	
IFITM1	INFLAMMATORY_RESPONSE, INTERFERON_ALPHA_RESPONSE
ARRDC3	
CA9	
SLC2A5	HYPOXIA, SPERMATOGENESIS
BNIP3	PROTEIN_SECRETION
ITGA5	EPITHELIAL_MESENCHYMAL_TRANSITION, INFLAMMATORY_RESPONSE
SLC39A14	
DEPP1	
LAMA4	ADIPOGENESIS
PHKA2	GLYCOLYSIS
TCF4	
PDE3A	
A2M	COAGULATION, IL6_JAK_STAT3_SIGNALING
**pRCC**	Hallmark gene sets (MSigDB 7.1)
LRRN4	
SLPI	KRAS_SIGNALING_UP
SLC34A2	
PROM1	
RELN	KRAS_SIGNALING_UP
SOSTDC1	
PIGR	KRAS_SIGNALING_UP
CNTN6	
SLC44A4	
UPK1B	
RBP4	KRAS_SIGNALING_UP, XENOBIOTIC_METABOLISM
CLDN3	GLYCOLYSIS
MMP7	COAGULATION
TSPAN1	KRAS_SIGNALING_UP
CLIC6	
SLC6A20	
BAMBI	
IL12RB2	SPERMATOGENESIS

MACC1	
CXCL6	ANGIOGENESIS, EPITHELIAL_MESENCHYMAL_TRANSITION, INFLAMMATORY_RESPONSE, TNFA_SIGNALING_VIA_NFKB
GPC4	GLYCOLYSIS, HYPOXIA
PTPRD	UV_RESPONSE_UP
IGFBP6	APOPTOSIS
LAMC2	APICAL_JUNCTION, EPITHELIAL_MESENCHYMAL_TRANSITION, ESTROGEN_RESPONSE_LATE
GALNT11	
TMEM139	
PAPPA	
HUNK	
SOX9	GLYCOLYSIS, KRAS_SIGNALING_UP
AIF1L	
CDHR2	
PRICKLE1	
SOX4	
PCLO	COMPLEMENT
IL17RD	
MYL3	MYOGENESIS
C17orf97	
ADCY2	PI3K_AKT_MTOR_SIGNALING
PTH1R	IL2_STAT5_SIGNALING
PGGHG	
ADGRA3	
ELF3	ESTROGEN_RESPONSE_EARLY, GLYCOLYSIS
LCN2	
TMEM163	
LAMB1	
FAM189A1	
SCARA3	
B3GALT5	
PAX2	
SLC15A2	
HYDIN	
FOXQ1	
AK7	
PLXNB1	ESTROGEN_RESPONSE_LATE
DCBLD2	APICAL_SURFACE, INFLAMMATORY_RESPONSE, KRAS_SIGNALING_UP
C21orf62	
NAPEPLD	
CNTN4	
**chRCC**	Hallmark gene sets (MSigDB 7.1)
ATP6V0D2	

SLC26A7	
PVALB	MYOGENESIS
STAP1	
CFTR	SPERMATOGENESIS
HEPACAM2	
ERP27	
CLNK	
RHCG	APICAL_SURFACE
CLDN8	APICAL_JUNCTION, KRAS_SIGNALING_DN
ATP6V1G3	
FOXI1	
ATP6V1C2	
SHOC1	
TMEM52B	
TMEM213	
BMPR1B	ANDROGEN_RESPONSE, FATTY_ACID_METABOLISM, KRAS_SIGNALING_DN
MUC20	
SLC4A1	HEME_METABOLISM
TMPRSS2	ANDROGEN_RESPONSE
MYZAP	
ATP6V1B1	KRAS_SIGNALING_DN, PROTEIN_SECRETION
CWH43	
EGF	G2M_CHECKPOINT, KRAS_SIGNALING_DN
CKMT2	MYOGENESIS
GRB14	
GPRC6A	
SEMA3C	PEROXISOME
SLC16A7	KRAS_SIGNALING_DN
SLC9A2	
CNTNAP5	
KLK1	COMPLEMENT
OXGR1	
NEDD4L	HYPOXIA
PRLR	ESTROGEN_RESPONSE_LATE
HS6ST3	
SLC2A12	
DHRS7	ADIPOGENESIS, XENOBIOTIC_METABOLISM
FYB2	
TMEM255A	
DMRT2	
GPAT3	
THRSP	
FXYD4	

STK32A	
RANBP3L	
FAM169A	
SYT17	
KLHL3	
TBC1D14	
UGT8	
PXK	
WNK3	
GALNT17	
PACRG	SPERMATOGENESIS
IL18	ALLOGRAFT_REJECTION, APOPTOSIS, INFLAMMATORY_RESPONSE, TNFA_SIGNALING_VIA_NFKB
PKIA	MYOGENESIS
ANGPTL1	
