# Supplementary material for: A novel molecular signature identifies mixed subtypes in renal cell carcinoma with poor prognosis and independent response to immunotherapy
Source: Genome Med. 2022 Sep 15;14:105. doi: 10.1186/s13073-022-01105-y (PMC9476269; doi:10.1186/s13073-022-01105-y)
Supplement: Supplementary file 7 — Additional file 7: Table S5. PSA of the TCGA RCC cohort (C3). [file 13073_2022_1105_MOESM7_ESM.pdf]

Additional file 7. Table S5. PSA of the TCGA RCC cohort (C3).

		Proportional subtype assignments					Pathological classification from Ricketts et al., 2018		Molecular classification from Chen et al., 2016
**Sample**	**Cohort**	**ccRCC**	**chRCC**	**pRCC**	**P_psa**	**risk_group**	**PanKidney.Pathology**	**Additional.Pathology.Data**	**final.subtype.call**
TCGA-B2-5633-01B-04R-A277-07	KIRC	99	0	1	0,0001	intermediate	ccRCC	Clear cell RCC	CC-e.1
TCGA-B2-3924-01B-03R-A277-07	KIRC	100	0	0	0,0085	intermediate	ccRCC	Clear cell RCC	CC-e.2
TCGA-B2-5635-01B-04R-A277-07	KIRC	88	0	12	0,0001	intermediate	ccRCC	Clear cell RCC	CC-e.2
TCGA-CJ-4882-01A-02R-1426-07	KIRC	96	1	3	0,0001	intermediate	ccRCC	Clear cell RCC	CC-e.3
TCGA-BP-4992-01A-01R-1334-07	KIRC	98	0	2	0,0023	intermediate	ccRCC	Clear cell RCC	CC-e.1
TCGA-BP-4351-01A-01R-1289-07	KIRC	100	0	0	0,0025	intermediate	ccRCC	Clear cell RCC	CC-e.2
TCGA-BP-4971-01A-01R-1334-07	KIRC	97	1	2	0,0002	intermediate	ccRCC	Clear cell RCC	CC-e.3
TCGA-AK-3454-01A-02R-1277-07	KIRC	100	0	0	0,0001	intermediate	ccRCC	Clear cell RCC	CC-e.1
TCGA-AK-3426-01A-02R-1325-07	KIRC	79	1	20	0,0005	intermediate			CC-e.3
TCGA-B2-3923-01B-10R-A277-07	KIRC	0	100	0	0,0001	good	ChRCC		Ch-e
TCGA-BP-4989-01A-01R-1334-07	KIRC	95	0	5	0,0001	intermediate	ccRCC	Clear cell RCC	CC-e.3
TCGA-B0-4811-01A-01R-1503-07	KIRC	94	0	6	0,0051	intermediate	ccRCC	Clear cell RCC	CC-e.2
TCGA-B0-4819-01A-01R-1277-07	KIRC	73	1	26	0,0001	intermediate	ccRCC	Clear cell RCC	CC-e.3
TCGA-AK-3453-01A-02R-1277-07	KIRC	96	0	4	0,0001	intermediate			mixed
TCGA-B0-4697-01A-01R-1277-07	KIRC	99	1	0	0,0236	intermediate	ccRCC	Clear cell RCC	CC-e.3
TCGA-B0-4714-01A-01R-1277-07	KIRC	100	0	0	0,0002	intermediate	ccRCC	Clear cell RCC	CC-e.2
TCGA-B0-4815-01A-01R-1503-07	KIRC	96	0	4	0,0001	intermediate	ccRCC	Clear cell RCC	CC-e.3
TCGA-B0-4843-01A-01R-1277-07	KIRC	99	0	1	0,0001	intermediate	ccRCC	Clear cell RCC	CC-e.3
TCGA-BP-4335-01A-01R-1289-07	KIRC	100	0	0	0,0001	intermediate	ccRCC	Clear cell RCC	CC-e.2
TCGA-A3-3347-01A-02R-1325-07	KIRC	95	0	5	0,0008	intermediate	ccRCC	Clear cell RCC	CC-e.3
TCGA-B0-5092-01A-01R-1420-07	KIRC	94	0	6	0,0019	intermediate	ccRCC	Clear cell RCC	CC-e.1
TCGA-CJ-4881-01A-01R-1305-07	KIRC	90	0	10	0,0001	intermediate	ccRCC	Clear cell RCC	CC-e.3
TCGA-CJ-4873-01A-01R-1305-07	KIRC	75	1	24	0,0053	intermediate	ccRCC	Clear cell RCC	CC-e.3
TCGA-MM-A563-01A-11R-A266-07	KIRC	89	1	10	0,0002	intermediate	ccRCC	Clear cell RCC	CC-e.1
TCGA-B0-4822-01A-01R-1277-07	KIRC	99	1	0	0,0001	intermediate	ccRCC	Clear cell RCC	CC-e.3
TCGA-B0-4821-01A-01R-1503-07	KIRC	99	0	1	0,0027	intermediate	ChRCC	Metabolically Divergent (MD-)ChRCC	CC-e.3
TCGA-BP-5010-01A-02R-1420-07	KIRC	93	0	7	0,0072	intermediate	ccRCC	Clear cell RCC	CC-e.3
TCGA-BP-4337-01A-01R-1289-07	KIRC	93	1	6	0,0001	intermediate	ccRCC	Clear cell RCC	CC-e.3
TCGA-BP-5199-01A-01R-1426-07	KIRC	99	0	1	0,0003	intermediate	ccRCC	Clear cell RCC	CC-e.3
TCGA-A3-3323-01A-02R-1325-07	KIRC	100	0	0	0,0001	intermediate	ccRCC	Clear cell RCC	CC-e.2
TCGA-B0-5095-01A-01R-1420-07	KIRC	100	0	0	0,0001	intermediate	ccRCC	Clear cell RCC	CC-e.3
TCGA-BP-4998-01A-01R-1334-07	KIRC	82	0	18	0,0001	intermediate	ccRCC	Clear cell RCC	CC-e.2
TCGA-BP-4346-01A-01R-1289-07	KIRC	91	0	9	0,0001	intermediate	ccRCC	Clear cell RCC	CC-e.1
TCGA-A3-3317-01A-02R-1325-07	KIRC	85	1	14	0,0001	intermediate	ccRCC	Clear cell RCC	CC-e.1
TCGA-B0-4706-01A-01R-1503-07	KIRC	99	0	1	0,0065	intermediate	ccRCC	Clear cell RCC	CC-e.2
TCGA-B0-4710-01A-01R-1503-07	KIRC	100	0	0	0,0011	intermediate	ccRCC	Clear cell RCC	CC-e.2
TCGA-CJ-4875-01A-01R-1305-07	KIRC	93	0	7	0,0001	intermediate	ccRCC	Clear cell RCC	CC-e.2
TCGA-CJ-4902-01A-01R-1426-07	KIRC	100	0	0	0,0001	intermediate	ccRCC	Clear cell RCC	CC-e.3
TCGA-B0-4847-01A-01R-1277-07	KIRC	96	0	4	0,0002	intermediate	ccRCC	Clear cell RCC	CC-e.2

TCGA-BP-4977-01A-01R-1334-07	KIRC	98	0	2	0,0005	intermediate	ccRCC	Clear cell RCC	CC-e.2
TCGA-B8-4143-01A-01R-1188-07	KIRC	100	0	0	0,0001	intermediate	ccRCC	Clear cell RCC	CC-e.3
TCGA-BP-4355-01A-01R-1289-07	KIRC	100	0	0	0,0002	intermediate	ccRCC	Clear cell RCC	CC-e.2
TCGA-B0-4718-01A-01R-1277-07	KIRC	99	1	0	0,0001	intermediate	ccRCC	Clear cell RCC	CC-e.2
TCGA-B0-5081-01A-01R-1334-07	KIRC	100	0	0	0,0001	intermediate	ccRCC	Clear cell RCC	CC-e.3
TCGA-B0-4833-01A-01R-1305-07	KIRC	99	0	1	0,0054	intermediate	ccRCC	Clear cell RCC	CC-e.2
TCGA-B2-4101-01A-02R-1277-07	KIRC	96	0	4	0,0001	intermediate	ccRCC	Clear cell RCC	CC-e.2
TCGA-B0-4810-01A-01R-1503-07	KIRC	96	0	4	0,0002	intermediate	ccRCC	Clear cell RCC	CC-e.3
TCGA-B8-A54F-01A-11R-A266-07	KIRC	96	0	4	0,0007	intermediate	ccRCC	Clear cell RCC	CC-e.2
TCGA-A3-3351-01A-02R-1325-07	KIRC	98	0	2	0,0001	intermediate	ccRCC	Clear cell RCC	CC-e.2
TCGA-CZ-4857-01A-01R-1305-07	KIRC	91	0	9	0,0002	intermediate	ccRCC	Clear cell RCC	CC-e.3
TCGA-CJ-4635-01A-02R-1305-07	KIRC	100	0	0	0,0001	intermediate	ccRCC	Clear cell RCC	CC-e.1
TCGA-CZ-4854-01A-01R-1305-07	KIRC	94	0	6	0,0001	intermediate	ccRCC	Clear cell RCC	CC-e.3
TCGA-BP-4804-01A-02R-1305-07	KIRC	95	0	5	0,0002	intermediate	ccRCC	Clear cell RCC	CC-e.3
TCGA-BP-5000-01A-01R-1334-07	KIRC	90	0	10	0,0001	intermediate	ccRCC	Clear cell RCC	CC-e.1
TCGA-BP-5001-01A-01R-1334-07	KIRC	96	1	3	0,0045	intermediate	ccRCC	Clear cell RCC	CC-e.2
TCGA-BP-4974-01A-01R-1334-07	KIRC	100	0	0	0,0001	intermediate	ccRCC	Clear cell RCC	CC-e.2
TCGA-B0-4707-01A-01R-1277-07	KIRC	90	0	10	0,0038	intermediate	ccRCC	Clear cell RCC	CC-e.3
TCGA-BP-4169-01A-02R-1289-07	KIRC	100	0	0	0,0001	intermediate	ccRCC	Clear cell RCC	CC-e.3
TCGA-BP-4349-01A-01R-1289-07	KIRC	99	0	1	0,0005	intermediate	ccRCC	Clear cell RCC	CC-e.2
TCGA-CZ-4862-01A-01R-1305-07	KIRC	83	0	17	0,0001	intermediate	ccRCC	Clear cell RCC	CC-e.2
TCGA-B0-5106-01A-01R-1420-07	KIRC	90	0	10	0,0001	intermediate	ccRCC	Clear cell RCC	CC-e.2
TCGA-CJ-4901-01A-01R-1426-07	KIRC	100	0	0	0,0001	intermediate	ccRCC	Clear cell RCC	CC-e.3
TCGA-B0-5088-01A-01R-1334-07	KIRC	99	0	1	0,0001	intermediate	ccRCC	Clear cell RCC	CC-e.2
TCGA-BP-5191-01A-01R-1426-07	KIRC	81	0	19	0,0004	intermediate	ccRCC	Clear cell RCC	CC-e.3
TCGA-BP-4960-01A-01R-1334-07	KIRC	100	0	0	0,0014	intermediate	ccRCC	Clear cell RCC	CC-e.1
TCGA-B0-4813-01A-01R-1277-07	KIRC	97	1	2	0,0038	intermediate	ccRCC	Clear cell RCC	CC-e.3
TCGA-B0-4817-01A-01R-1277-07	KIRC	73	0	27	0,0177	intermediate	ccRCC	Clear cell RCC	CC-e.3
TCGA-CJ-4636-01A-02R-1325-07	KIRC	80	1	19	0,0001	intermediate	ccRCC	Clear cell RCC	CC-e.1
TCGA-B8-A54D-01A-21R-A266-07	KIRC	84	0	16	0,0001	intermediate	ccRCC	Clear cell RCC	CC-e.2
TCGA-B0-5085-01A-01R-1334-07	KIRC	100	0	0	0,0135	intermediate	ccRCC	Clear cell RCC	CC-e.2
TCGA-CJ-4637-01A-02R-1325-07	KIRC	90	1	9	0,0001	intermediate	ccRCC	Clear cell RCC	CC-e.3
TCGA-B0-5077-01A-01R-1334-07	KIRC	89	0	11	0,0001	intermediate	ccRCC	Clear cell RCC	CC-e.2
TCGA-A3-3370-01A-02R-1420-07	KIRC	99	0	1	0,0031	intermediate	ccRCC	Clear cell RCC	CC-e.2
TCGA-BP-4327-01A-01R-1289-07	KIRC	97	1	2	0,0001	intermediate	ccRCC	Clear cell RCC	CC-e.1
TCGA-B0-4824-01A-01R-1277-07	KIRC	100	0	0	0,002	intermediate	ccRCC	Clear cell RCC	CC-e.2
TCGA-CJ-4912-01A-01R-1426-07	KIRC	92	0	8	0,0022	intermediate	ccRCC	Clear cell RCC	CC-e.1
TCGA-B8-A54I-01A-21R-A33J-07	KIRC	84	1	15	0,0001	intermediate	ccRCC	Clear cell RCC	CC-e.3
TCGA-BP-4330-01A-01R-1289-07	KIRC	100	0	0	0,0001	intermediate	ccRCC	Clear cell RCC	CC-e.2
TCGA-BP-4775-01A-01R-1289-07	KIRC	100	0	0	0,0001	intermediate	ccRCC	Clear cell RCC	CC-e.2
TCGA-A3-3316-01A-01R-0864-07	KIRC	100	0	0	0,0001	intermediate	ccRCC	Clear cell RCC	CC-e.3
TCGA-B0-5084-01A-01R-1334-07	KIRC	45	1	54	0,0151	poor			P.CIMP-e
TCGA-CJ-4872-01A-01R-1305-07	KIRC	100	0	0	0,0001	intermediate	ccRCC	Clear cell RCC	CC-e.2
TCGA-CJ-4899-01A-01R-1334-07	KIRC	97	0	3	0,0001	intermediate	ccRCC	Clear cell RCC	CC-e.2

TCGA-B0-4828-01A-01R-1277-07	KIRC	96	0	4	0,0117	intermediate	ccRCC	Clear cell RCC	CC-e.1
TCGA-BP-5169-01A-01R-1426-07	KIRC	99	1	0	0,0031	intermediate	ccRCC	Clear cell RCC	CC-e.3
TCGA-BP-5008-01A-01R-1334-07	KIRC	92	0	8	0,0001	intermediate	ccRCC	Clear cell RCC	CC-e.2
TCGA-CJ-4908-01A-01R-1426-07	KIRC	100	0	0	0,0001	intermediate	ccRCC	Clear cell RCC	CC-e.2
TCGA-BP-4798-01A-01R-1305-07	KIRC	98	0	2	0,0055	intermediate	ccRCC	Clear cell RCC	CC-e.3
TCGA-CJ-4868-01A-01R-1305-07	KIRC	100	0	0	0,0001	intermediate	ccRCC	Clear cell RCC	CC-e.3
TCGA-B0-4852-01A-01R-1503-07	KIRC	100	0	0	0,0001	intermediate	ccRCC	Clear cell RCC	CC-e.2
TCGA-CJ-4892-01A-01R-1305-07	KIRC	97	0	3	0,0019	intermediate	ccRCC	Clear cell RCC	CC-e.2
TCGA-CJ-4641-01A-02R-1325-07	KIRC	69	1	30	0,0002	poor	ccRCC	Clear cell RCC	CC-e.2
TCGA-BP-4332-01A-01R-1289-07	KIRC	88	1	11	0,0001	intermediate	ccRCC	Clear cell RCC	CC-e.2
TCGA-BP-5009-01A-01R-1334-07	KIRC	95	0	5	0,0001	intermediate	ccRCC	Clear cell RCC	CC-e.1
TCGA-BP-4345-01A-01R-1289-07	KIRC	99	1	0	0,0001	intermediate	ccRCC	Clear cell RCC	CC-e.3
TCGA-CJ-4887-01A-01R-1305-07	KIRC	98	0	2	0,0008	intermediate	ccRCC	Clear cell RCC	CC-e.1
TCGA-A3-3322-01A-02R-1325-07	KIRC	100	0	0	0,0001	intermediate	ccRCC	Clear cell RCC	CC-e.2
TCGA-CJ-4920-01A-01R-1426-07	KIRC	100	0	0	0,0001	intermediate	ccRCC	Clear cell RCC	CC-e.2
TCGA-MM-A564-01A-11R-A266-07	KIRC	100	0	0	0,0008	intermediate	ccRCC	Clear cell RCC	CC-e.1
TCGA-A3-3349-01A-01R-1188-07	KIRC	100	0	0	0,0001	intermediate	ccRCC	Clear cell RCC	CC-e.2
TCGA-A3-3325-01A-01R-0864-07	KIRC	96	0	4	0,0009	intermediate	ccRCC	Clear cell RCC	CC-e.1
TCGA-B0-4836-01A-01R-1305-07	KIRC	100	0	0	0,0001	intermediate	ccRCC	Clear cell RCC	CC-e.3
TCGA-BP-4161-01A-02R-1325-07	KIRC	100	0	0	0,0002	intermediate	ccRCC	Clear cell RCC	CC-e.1
TCGA-A3-3319-01A-02R-1325-07	KIRC	66	0	34	0,0001	poor	ccRCC	Clear cell RCC	CC-e.3
TCGA-CJ-4638-01A-02R-1325-07	KIRC	56	1	43	0,0398	poor	ccRCC	Clear cell RCC	CC-e.1
TCGA-A3-3362-01A-02R-1325-07	KIRC	90	1	9	0,0017	intermediate	ccRCC	Clear cell RCC	CC-e.2
TCGA-B0-4690-01A-01R-1277-07	KIRC	95	0	5	0,0001	intermediate	ccRCC	Clear cell RCC	CC-e.3
TCGA-B0-4691-01A-01R-1277-07	KIRC	77	1	22	0,0071	intermediate	ccRCC	Clear cell RCC	CC-e.1
TCGA-B0-5399-01A-01R-1503-07	KIRC	100	0	0	0,0002	intermediate	ccRCC	Clear cell RCC	CC-e.2
TCGA-B0-4701-01A-01R-1277-07	KIRC	100	0	0	0,002	intermediate	ccRCC	Clear cell RCC	CC-e.2
TCGA-A3-3376-01A-02R-1420-07	KIRC	99	0	1	0,0001	intermediate	ccRCC	Clear cell RCC	CC-e.2
TCGA-BP-4341-01A-01R-1289-07	KIRC	100	0	0	0,0018	intermediate	ccRCC	Clear cell RCC	CC-e.2
TCGA-A3-A8OX-01A-11R-A37O-07	KIRC	92	0	8	0,0027	intermediate	ccRCC	Clear cell RCC	CC-e.2
TCGA-A3-3308-01A-02R-1325-07	KIRC	100	0	0	0,0001	intermediate	ccRCC	Clear cell RCC	CC-e.3
TCGA-BP-4173-01A-02R-1289-07	KIRC	93	0	7	0,0001	intermediate	ccRCC	Clear cell RCC	CC-e.2
TCGA-A3-3326-01A-01R-0864-07	KIRC	100	0	0	0,0013	intermediate	ccRCC	Clear cell RCC	CC-e.2
TCGA-B0-4837-01A-01R-1305-07	KIRC	100	0	0	0,0002	intermediate	ccRCC	Clear cell RCC	CC-e.1
TCGA-A3-3358-01A-01R-1541-07	KIRC	100	0	0	0,0001	intermediate	ccRCC	Clear cell RCC	CC-e.2
TCGA-A3-3380-01A-01R-0864-07	KIRC	100	0	0	0,0014	intermediate	ccRCC	Clear cell RCC	CC-e.2
TCGA-B2-4102-01A-02R-1325-07	KIRC	91	0	9	0,0002	intermediate	ccRCC	Clear cell RCC	CC-e.2
TCGA-B0-4700-01A-02R-1541-07	KIRC	98	0	2	0,0003	intermediate	ccRCC	Clear cell RCC	CC-e.3
TCGA-BP-4963-01A-01R-1334-07	KIRC	100	0	0	0,0001	intermediate	ccRCC	Clear cell RCC	CC-e.1
TCGA-BP-4776-01A-01R-1289-07	KIRC	98	0	2	0,0024	intermediate	ccRCC	Clear cell RCC	CC-e.2
TCGA-B0-5094-01A-01R-1420-07	KIRC	91	0	9	0,0001	intermediate	ccRCC	Clear cell RCC	CC-e.3
TCGA-B2-A4SR-01A-11R-A266-07	KIRC	100	0	0	0,0001	intermediate	ccRCC	Clear cell RCC	CC-e.2
TCGA-B0-5109-01A-02R-1420-07	KIRC	79	1	20	0,0001	intermediate	ccRCC	Clear cell RCC	CC-e.3
TCGA-AK-3461-01A-02R-1277-07	KIRC	95	0	5	0,0023	intermediate	ccRCC	Clear cell RCC	CC-e.2

TCGA-BP-4981-01A-01R-1334-07	KIRC	94	0	6	0,0013	intermediate	ccRCC	Clear cell RCC	CC-e.2
TCGA-CJ-4890-01A-01R-1305-07	KIRC	94	1	5	0,0001	intermediate	ccRCC	Clear cell RCC	CC-e.3
TCGA-BP-4991-01A-01R-1334-07	KIRC	100	0	0	0,0001	intermediate	ccRCC	Clear cell RCC	CC-e.2
TCGA-BP-4968-01A-01R-1334-07	KIRC	94	0	6	0,0009	intermediate	ccRCC	Clear cell RCC	CC-e.2
TCGA-B0-4699-01A-01R-1277-07	KIRC	15	85	0	0,0002	intermediate	ChRCC	Metabolically Divergent (MD-)ChRCC	Ch-e
TCGA-A3-3387-01A-01R-1541-07	KIRC	100	0	0	0,0001	intermediate	ccRCC	Clear cell RCC	CC-e.1
TCGA-CJ-4889-01A-01R-1305-07	KIRC	96	0	4	0,0001	intermediate	ccRCC	Clear cell RCC	CC-e.3
TCGA-AK-3458-01A-01R-1503-07	KIRC	94	0	6	0,0018	intermediate	ccRCC	Clear cell RCC	CC-e.1
TCGA-B8-4620-01A-02R-1325-07	KIRC	100	0	0	0,0002	intermediate	ccRCC	Clear cell RCC	CC-e.3
TCGA-B8-4622-01A-02R-1277-07	KIRC	100	0	0	0,0002	intermediate	ccRCC	Clear cell RCC	CC-e.2
TCGA-CJ-4643-01A-02R-1325-07	KIRC	100	0	0	0,0002	intermediate	ccRCC	Clear cell RCC	CC-e.2
TCGA-B0-4703-01A-01R-1277-07	KIRC	100	0	0	0,0001	intermediate	ccRCC	Clear cell RCC	CC-e.3
TCGA-BP-4790-01A-01R-1305-07	KIRC	98	0	2	0,0001	intermediate	ccRCC	Clear cell RCC	CC-e.2
TCGA-AS-3778-01A-01R-A32Z-07	KIRC	95	0	5	0,0001	intermediate	ccRCC	Clear cell RCC	CC-e.2
TCGA-CJ-4916-01A-01R-1426-07	KIRC	100	0	0	0,0005	intermediate	ccRCC	Clear cell RCC	CC-e.2
TCGA-G6-A8L7-01A-11R-A37O-07	KIRC	96	2	2	0,0001	intermediate	ccRCC	Clear cell RCC	CC-e.3
TCGA-BP-5007-01A-01R-1334-07	KIRC	95	0	5	0,0002	intermediate	ccRCC	Clear cell RCC	CC-e.2
TCGA-A3-A6NL-01A-11R-A33J-07	KIRC	100	0	0	0,0001	intermediate	ccRCC	Clear cell RCC	CC-e.2
TCGA-BP-4969-01A-01R-1334-07	KIRC	100	0	0	0,0121	intermediate	ccRCC	Clear cell RCC	CC-e.2
TCGA-CJ-4894-01A-01R-1305-07	KIRC	93	0	7	0,0001	intermediate	ccRCC	Clear cell RCC	CC-e.2
TCGA-AK-3445-01A-02R-1277-07	KIRC	99	0	1	0,0001	intermediate	ccRCC	Clear cell RCC	CC-e.3
TCGA-A3-3359-01A-01R-0864-07	KIRC	97	0	3	0,0016	intermediate	ccRCC	Clear cell RCC	CC-e.2
TCGA-BP-5201-01A-01R-1426-07	KIRC	90	0	10	0,0001	intermediate	ccRCC	Clear cell RCC	CC-e.1
TCGA-BP-5175-01A-01R-1426-07	KIRC	64	0	36	0,009	poor	ccRCC	Clear cell RCC	CC-e.3
TCGA-BP-4331-01A-01R-1289-07	KIRC	88	0	12	0,0001	intermediate	ccRCC	Clear cell RCC	CC-e.1
TCGA-A3-A6NN-01A-12R-A33J-07	KIRC	100	0	0	0,0001	intermediate	ccRCC	Clear cell RCC	CC-e.2
TCGA-B0-5691-01A-11R-1541-07	KIRC	86	1	13	0,0001	intermediate	ccRCC	Clear cell RCC	CC-e.2
TCGA-B0-5108-01A-01R-1420-07	KIRC	99	1	0	0,0001	intermediate	ccRCC	Clear cell RCC	CC-e.3
TCGA-MM-A84U-01A-11R-A37O-07	KIRC	83	1	16	0,0061	intermediate	ccRCC	Clear cell RCC	CC-e.1
TCGA-A3-3378-01A-02R-1325-07	KIRC	93	0	7	0,0001	intermediate	ccRCC	Clear cell RCC	CC-e.3
TCGA-CZ-5464-01A-01R-1503-07	KIRC	96	0	4	0,0012	intermediate	ccRCC	Clear cell RCC	CC-e.3
TCGA-CJ-4888-01A-01R-1305-07	KIRC	100	0	0	0,0001	intermediate	ccRCC	Clear cell RCC	CC-e.3
TCGA-BP-4982-01A-01R-1334-07	KIRC	100	0	0	0,0001	intermediate	ccRCC	Clear cell RCC	CC-e.2
TCGA-BP-4165-01A-02R-1289-07	KIRC	100	0	0	0,0017	intermediate	ccRCC	Clear cell RCC	CC-e.2
TCGA-B0-5115-01A-01R-1420-07	KIRC	100	0	0	0,0001	intermediate	ccRCC	Clear cell RCC	CC-e.2
TCGA-A3-3372-01A-02R-1325-07	KIRC	100	0	0	0,0001	intermediate	ccRCC	Clear cell RCC	CC-e.1
TCGA-B2-4098-01A-02R-1325-07	KIRC	8	1	91	0,0001	good	ccRCC	Clear cell RCC	P-e.2
TCGA-BP-4964-01A-01R-1334-07	KIRC	100	0	0	0,0001	intermediate	ccRCC	Clear cell RCC	CC-e.2
TCGA-BP-4777-01A-01R-1289-07	KIRC	94	0	6	0,0001	intermediate	ccRCC	Clear cell RCC	CC-e.1
TCGA-A3-A8OU-01A-11R-A37O-07	KIRC	93	0	7	0,0031	intermediate	ccRCC	Clear cell RCC	CC-e.2
TCGA-CJ-4891-01A-01R-1305-07	KIRC	94	0	6	0,0012	intermediate	ccRCC	Clear cell RCC	CC-e.3
TCGA-BP-4787-01A-01R-1305-07	KIRC	95	0	5	0,0001	intermediate	ccRCC	Clear cell RCC	CC-e.3
TCGA-BP-4973-01A-01R-1334-07	KIRC	100	0	0	0,0001	intermediate	ccRCC	Clear cell RCC	CC-e.1
TCGA-A3-3331-01A-02R-1325-07	KIRC	100	0	0	0,0001	intermediate	ccRCC	Clear cell RCC	CC-e.2

TCGA-B8-4148-01A-02R-1325-07	KIRC	100	0	0	0,0001	intermediate	ccRCC	Clear cell RCC	CC-e.2
TCGA-BP-4347-01A-01R-1289-07	KIRC	100	0	0	0,0001	intermediate	ccRCC	Clear cell RCC	CC-e.1
TCGA-BP-4158-01A-02R-1289-07	KIRC	100	0	0	0,0001	intermediate	ccRCC	Clear cell RCC	CC-e.2
TCGA-BP-4999-01A-01R-1334-07	KIRC	100	0	0	0,0025	intermediate	ccRCC	Clear cell RCC	CC-e.2
TCGA-B0-4814-01A-01R-1277-07	KIRC	100	0	0	0,0003	intermediate	ccRCC	Clear cell RCC	CC-e.1
TCGA-B8-A8YJ-01A-13R-A39I-07	KIRC	100	0	0	0,0033	intermediate	ccRCC	Clear cell RCC	CC-e.2
TCGA-B0-5100-01A-01R-1420-07	KIRC	83	1	16	0,0029	intermediate	ccRCC	Clear cell RCC	mixed
TCGA-A3-3365-01A-01R-0864-07	KIRC	99	1	0	0,0001	intermediate	ccRCC	Clear cell RCC	CC-e.2
TCGA-BP-4758-01A-01R-1289-07	KIRC	93	0	7	0,0001	intermediate	ccRCC	Clear cell RCC	CC-e.2
TCGA-B0-4844-01A-01R-1277-07	KIRC	100	0	0	0,0007	intermediate	ccRCC	Clear cell RCC	CC-e.1
TCGA-CJ-4895-01A-01R-1305-07	KIRC	99	1	0	0,0001	intermediate	ccRCC	Clear cell RCC	CC-e.3
TCGA-BP-4774-01A-01R-1289-07	KIRC	100	0	0	0,0022	intermediate	ccRCC	Clear cell RCC	CC-e.2
TCGA-B0-4849-01A-01R-1277-07	KIRC	100	0	0	0,0001	intermediate	ccRCC	Clear cell RCC	CC-e.1
TCGA-CJ-4918-01A-01R-1426-07	KIRC	99	0	1	0,0001	intermediate	ccRCC	Clear cell RCC	CC-e.3
TCGA-B0-5120-01A-01R-1420-07	KIRC	100	0	0	0,0001	intermediate	ccRCC	Clear cell RCC	CC-e.2
TCGA-A3-3367-01A-02R-1420-07	KIRC	100	0	0	0,0005	intermediate	ccRCC	Clear cell RCC	CC-e.2
TCGA-B0-5121-01A-02R-1420-07	KIRC	100	0	0	0,0004	intermediate	ccRCC	Clear cell RCC	CC-e.2
TCGA-CZ-5459-01A-01R-1503-07	KIRC	96	0	4	0,0033	intermediate	ccRCC	Clear cell RCC	CC-e.2
TCGA-A3-A8OW-01A-11R-A37O-07	KIRC	99	0	1	0,0017	intermediate	ccRCC	Clear cell RCC	CC-e.2
TCGA-BP-4756-01A-01R-1289-07	KIRC	54	5	41	0,1568	poor	ccRCC	Clear cell RCC	P-e.2
TCGA-BP-4176-01A-02R-1289-07	KIRC	93	0	7	0,0002	intermediate	ccRCC	Clear cell RCC	CC-e.3
TCGA-AK-3460-01A-02R-1277-07	KIRC	100	0	0	0,0011	intermediate	ccRCC	Clear cell RCC	CC-e.2
TCGA-B0-4945-01A-01R-1420-07	KIRC	89	0	11	0,0001	intermediate	ccRCC	Clear cell RCC	CC-e.2
TCGA-B0-5113-01A-01R-1420-07	KIRC	100	0	0	0,0001	intermediate	ccRCC	Clear cell RCC	CC-e.2
TCGA-CZ-5469-01A-01R-1503-07	KIRC	83	0	17	0,0053	intermediate	ccRCC	Clear cell RCC	CC-e.3
TCGA-MW-A4EC-01A-11R-A266-07	KIRC	96	0	4	0,0013	intermediate	ccRCC	Clear cell RCC	CC-e.2
TCGA-B0-4818-01A-01R-1503-07	KIRC	100	0	0	0,0028	intermediate	ccRCC	Clear cell RCC	CC-e.2
TCGA-B0-4816-01A-01R-1503-07	KIRC	95	0	5	0,0002	intermediate	ccRCC	Clear cell RCC	CC-e.2
TCGA-BP-4167-01A-02R-1325-07	KIRC	100	0	0	0,0024	intermediate	ccRCC	Clear cell RCC	CC-e.3
TCGA-B0-4713-01A-01R-1277-07	KIRC	80	0	20	0,0043	intermediate	ccRCC	Clear cell RCC	CC-e.1
TCGA-B0-4694-01A-01R-1277-07	KIRC	90	0	10	0,0001	intermediate	ccRCC	Clear cell RCC	CC-e.3
TCGA-BP-4995-01A-01R-1334-07	KIRC	32	68	0	0,0001	poor	ccRCC	Clear cell RCC	CC-e.1
TCGA-A3-3352-01A-01R-0864-07	KIRC	100	0	0	0,0001	intermediate	ccRCC	Clear cell RCC	CC-e.1
TCGA-CJ-4884-01A-01R-1305-07	KIRC	94	0	6	0,0002	intermediate	ccRCC	Clear cell RCC	CC-e.2
TCGA-BP-5196-01A-01R-1426-07	KIRC	92	0	8	0,0001	intermediate	ccRCC	Clear cell RCC	CC-e.3
TCGA-A3-3324-01A-02R-1325-07	KIRC	100	0	0	0,0001	intermediate	ccRCC	Clear cell RCC	CC-e.1
TCGA-B8-A54K-01A-11R-A33J-07	KIRC	77	1	22	0,0001	intermediate	ccRCC	Clear cell RCC	mixed
TCGA-A3-A8CQ-01A-11R-A37O-07	KIRC	100	0	0	0,0014	intermediate	ccRCC	Clear cell RCC	CC-e.2
TCGA-BP-4967-01A-01R-1334-07	KIRC	84	0	16	0,0001	intermediate	ccRCC	Clear cell RCC	CC-e.2
TCGA-BP-4994-01A-01R-1334-07	KIRC	16	84	0	0,0001	intermediate	ChRCC		Ch-e
TCGA-CJ-4907-01A-01R-1426-07	KIRC	98	0	2	0,0001	intermediate	ccRCC	Clear cell RCC	CC-e.1
TCGA-BP-4325-01A-02R-1289-07	KIRC	100	0	0	0,0004	intermediate	ccRCC	Clear cell RCC	CC-e.1
TCGA-DV-5574-01A-01R-1541-07	KIRC	88	2	10	0,0001	intermediate	ccRCC	Clear cell RCC	CC-e.3
TCGA-A3-3382-01A-02R-1325-07	KIRC	100	0	0	0,0001	intermediate	ccRCC	Clear cell RCC	CC-e.3

TCGA-B0-4841-01A-01R-1277-07	KIRC	100	0	0	0,0002	intermediate	ccRCC	Clear cell RCC	CC-e.2
TCGA-BP-4326-01A-01R-1289-07	KIRC	100	0	0	0,0001	intermediate	ccRCC	Clear cell RCC	CC-e.3
TCGA-B0-4838-01A-01R-1305-07	KIRC	99	0	1	0,0003	intermediate	ccRCC	Clear cell RCC	CC-e.2
TCGA-B2-4099-01A-02R-1188-07	KIRC	100	0	0	0,0017	intermediate	ccRCC	Clear cell RCC	CC-e.2
TCGA-CJ-6031-01A-11R-1672-07	KIRC	90	1	9	0,0001	intermediate	ccRCC	Clear cell RCC	CC-e.3
TCGA-CZ-5984-01A-11R-1672-07	KIRC	69	1	30	0,0001	poor	ccRCC	Clear cell RCC	CC-e.3
TCGA-CJ-4639-01A-02R-1325-07	KIRC	100	0	0	0,0001	intermediate	ccRCC	Clear cell RCC	CC-e.2
TCGA-AK-3434-01A-02R-1277-07	KIRC	100	0	0	0,0001	intermediate	ccRCC	Clear cell RCC	CC-e.1
TCGA-BP-4352-01A-01R-1289-07	KIRC	94	1	5	0,0001	intermediate	ccRCC	Clear cell RCC	CC-e.3
TCGA-CZ-5466-01A-01R-1503-07	KIRC	90	1	9	0,0019	intermediate	ccRCC	Clear cell RCC	CC-e.1
TCGA-B8-5165-01A-01R-1420-07	KIRC	97	3	0	0,0001	intermediate	ccRCC	Clear cell RCC	CC-e.2
TCGA-CJ-4886-01A-01R-1305-07	KIRC	100	0	0	0,0001	intermediate	ccRCC	Clear cell RCC	CC-e.2
TCGA-BP-4765-01A-01R-1289-07	KIRC	100	0	0	0,0001	intermediate	ccRCC	Clear cell RCC	CC-e.2
TCGA-A3-3320-01A-02R-1325-07	KIRC	100	0	0	0,0012	intermediate	ccRCC	Clear cell RCC	CC-e.2
TCGA-BP-4766-01A-01R-1289-07	KIRC	100	0	0	0,0002	intermediate	ccRCC	Clear cell RCC	CC-e.2
TCGA-CZ-4864-01A-01R-1503-07	KIRC	100	0	0	0,0001	intermediate	ccRCC	Clear cell RCC	CC-e.1
TCGA-B8-5552-01B-11R-1672-07	KIRC	91	0	9	0,0001	intermediate	ccRCC	Clear cell RCC	CC-e.2
TCGA-A3-A6NI-01A-11R-A33J-07	KIRC	80	0	20	0,0042	intermediate	ccRCC	Clear cell RCC	CC-e.2
TCGA-G6-A8L6-01A-11R-A37O-07	KIRC	100	0	0	0,0027	intermediate	ccRCC	Clear cell RCC	CC-e.2
TCGA-CJ-5689-01A-11R-1541-07	KIRC	93	3	4	0,0001	intermediate	ccRCC	Clear cell RCC	CC-e.2
TCGA-3Z-A93Z-01A-11R-A37O-07	KIRC	87	1	12	0,0012	intermediate	ccRCC	Clear cell RCC	CC-e.2
TCGA-B0-5098-01A-01R-1420-07	KIRC	45	2	53	0,0025	poor			P.CIMP-e
TCGA-CZ-4863-01A-01R-1503-07	KIRC	84	0	16	0,0015	intermediate	ccRCC	Clear cell RCC	CC-e.2
TCGA-B8-A54J-01A-11R-A33J-07	KIRC	100	0	0	0,0001	intermediate	ccRCC	Clear cell RCC	CC-e.2
TCGA-EU-5905-01A-11R-1672-07	KIRC	100	0	0	0,0001	intermediate	ccRCC	Clear cell RCC	CC-e.3
TCGA-CJ-4903-01A-01R-1426-07	KIRC	100	0	0	0,0001	intermediate	ccRCC	Clear cell RCC	CC-e.1
TCGA-B0-5102-01A-01R-1420-07	KIRC	80	1	19	0,0002	intermediate	ccRCC	Clear cell RCC	CC-e.2
TCGA-B0-5697-01A-11R-1541-07	KIRC	31	0	69	0,0002	poor	ccRCC	Clear cell RCC	CC-e.2
TCGA-CJ-5672-01A-11R-1541-07	KIRC	88	0	12	0,0007	intermediate	ccRCC	Clear cell RCC	CC-e.1
TCGA-BP-4972-01A-01R-1334-07	KIRC	97	0	3	0,0001	intermediate	ccRCC	Clear cell RCC	CC-e.2
TCGA-CW-5589-01A-01R-1541-07	KIRC	92	1	7	0,0001	intermediate	ccRCC	Clear cell RCC	CC-e.2
TCGA-BP-4769-01A-01R-1289-07	KIRC	82	2	16	0,0001	intermediate			mixed
TCGA-AK-3455-01A-01R-0864-07	KIRC	93	0	7	0,0051	intermediate	ccRCC	Clear cell RCC	CC-e.2
TCGA-B0-5099-01A-01R-1420-07	KIRC	100	0	0	0,0001	intermediate	ccRCC	Clear cell RCC	CC-e.2
TCGA-BP-4987-01A-01R-1334-07	KIRC	100	0	0	0,0001	intermediate	ccRCC	Clear cell RCC	CC-e.2
TCGA-BP-5185-01A-01R-1426-07	KIRC	98	0	2	0,0022	intermediate	ccRCC	Clear cell RCC	CC-e.1
TCGA-BP-4768-01A-01R-1289-07	KIRC	89	0	11	0,0001	intermediate	ccRCC	Clear cell RCC	CC-e.1
TCGA-DV-A4VX-01A-11R-A266-07	KIRC	83	2	15	0,0007	intermediate	ccRCC	Clear cell RCC	CC-e.3
TCGA-BP-4795-01A-02R-1420-07	KIRC	78	2	20	0,0001	intermediate			CC-e.2
TCGA-AK-3465-01A-02R-1325-07	KIRC	4	93	3	0,0001	good	ChRCC		Ch-e
TCGA-B0-5400-01A-01R-1503-07	KIRC	95	1	4	0,0022	intermediate	ccRCC	Clear cell RCC	CC-e.3
TCGA-CJ-4634-01A-02R-1325-07	KIRC	100	0	0	0,0005	intermediate	ccRCC	Clear cell RCC	CC-e.2
TCGA-B0-5110-01A-01R-1420-07	KIRC	100	0	0	0,0001	intermediate	ccRCC	Clear cell RCC	CC-e.2
TCGA-B8-A54H-01A-11R-A33J-07	KIRC	95	0	5	0,0001	intermediate	ccRCC	Clear cell RCC	CC-e.2

TCGA-CZ-5458-01A-01R-1503-07	KIRC	96	0	4	0,0001	intermediate	ccRCC	Clear cell RCC	CC-e.2
TCGA-B8-A54G-01A-11R-A266-07	KIRC	93	0	7	0,0001	intermediate	ccRCC	Clear cell RCC	CC-e.1
TCGA-CZ-4858-01A-01R-1305-07	KIRC	99	1	0	0,0001	intermediate	ccRCC	Clear cell RCC	CC-e.3
TCGA-BP-5183-01A-01R-1426-07	KIRC	98	0	2	0,0002	intermediate	ccRCC	Clear cell RCC	CC-e.1
TCGA-B0-5709-01A-11R-1541-07	KIRC	90	0	10	0,0001	intermediate	ccRCC	Clear cell RCC	CC-e.2
TCGA-B0-5706-01A-11R-1541-07	KIRC	100	0	0	0,0001	intermediate	ccRCC	Clear cell RCC	CC-e.3
TCGA-A3-3335-01A-01R-0864-07	KIRC	100	0	0	0,0001	intermediate			CC-e.1
TCGA-DV-5576-01A-01R-1541-07	KIRC	85	2	13	0,0002	intermediate	ccRCC	Clear cell RCC	mixed
TCGA-B0-4693-01A-01R-1277-07	KIRC	95	0	5	0,0001	intermediate	ccRCC	Clear cell RCC	CC-e.2
TCGA-A3-3329-01A-01R-0864-07	KIRC	100	0	0	0,0001	intermediate	ccRCC	Clear cell RCC	CC-e.2
TCGA-B0-5097-01A-01R-1420-07	KIRC	100	0	0	0,0001	intermediate	ccRCC	Clear cell RCC	CC-e.3
TCGA-BP-4162-01A-02R-1325-07	KIRC	100	0	0	0,0005	intermediate	ccRCC	Clear cell RCC	CC-e.2
TCGA-BP-5177-01A-01R-1426-07	KIRC	95	0	5	0,0001	intermediate	ccRCC	Clear cell RCC	CC-e.2
TCGA-B8-5545-01A-01R-1672-07	KIRC	89	0	11	0,0001	intermediate	ccRCC	Clear cell RCC	CC-e.2
TCGA-A3-3346-01A-01R-1766-07	KIRC	97	0	3	0,0003	intermediate	ccRCC	Clear cell RCC	CC-e.3
TCGA-CZ-5470-01A-01R-1503-07	KIRC	84	0	16	0,0004	intermediate	ccRCC	Clear cell RCC	CC-e.1
TCGA-B0-5694-01A-11R-1541-07	KIRC	96	0	4	0,0002	intermediate	ccRCC	Clear cell RCC	CC-e.1
TCGA-A3-3307-01A-01R-0864-07	KIRC	100	0	0	0,0002	intermediate	ccRCC	Clear cell RCC	CC-e.2
TCGA-CJ-5671-01A-11R-1541-07	KIRC	99	1	0	0,0001	intermediate	ccRCC	Clear cell RCC	CC-e.3
TCGA-BP-4993-01A-02R-1420-07	KIRC	97	0	3	0,0001	intermediate	ccRCC	Clear cell RCC	CC-e.1
TCGA-6D-AA2E-01A-11R-A37O-07	KIRC	39	3	58	0,0049	poor	ccRCC	Clear cell RCC	P-e.2
TCGA-A3-3311-01A-02R-1325-07	KIRC	98	0	2	0,0001	intermediate	ccRCC	Clear cell RCC	CC-e.3
TCGA-B8-5158-01A-01R-1420-07	KIRC	100	0	0	0,0001	intermediate	ccRCC	Clear cell RCC	CC-e.3
TCGA-BP-4803-01A-01R-1305-07	KIRC	100	0	0	0,0001	intermediate	ccRCC	Clear cell RCC	CC-e.1
TCGA-BP-5187-01A-01R-1426-07	KIRC	100	0	0	0,0001	intermediate	ccRCC	Clear cell RCC	CC-e.2
TCGA-B0-5080-01A-01R-1503-07	KIRC	97	0	3	0,0001	intermediate	ccRCC	Clear cell RCC	CC-e.3
TCGA-DV-A4W0-01A-11R-A266-07	KIRC	92	0	8	0,002	intermediate	ccRCC	Clear cell RCC	CC-e.1
TCGA-G6-A8L8-01A-21R-A37O-07	KIRC	100	0	0	0,0002	intermediate	ccRCC	Clear cell RCC	CC-e.2
TCGA-A3-3313-01A-02R-1325-07	KIRC	63	4	33	0,0033	poor			P-e.2
TCGA-BP-4781-01A-01R-1305-07	KIRC	90	0	10	0,0001	intermediate	ccRCC	Clear cell RCC	CC-e.1
TCGA-BP-4970-01A-01R-1334-07	KIRC	85	0	15	0,0001	intermediate	ccRCC	Clear cell RCC	CC-e.2
TCGA-CJ-6033-01A-11R-1672-07	KIRC	99	0	1	0,0001	intermediate	ccRCC	Clear cell RCC	CC-e.3
TCGA-A3-3343-01A-01R-0864-07	KIRC	100	0	0	0,0004	intermediate	ccRCC	Clear cell RCC	CC-e.2
TCGA-B0-5402-01A-01R-1503-07	KIRC	100	0	0	0,0022	intermediate	ccRCC	Clear cell RCC	CC-e.1
TCGA-BP-4166-01A-02R-1289-07	KIRC	100	0	0	0,0001	intermediate	ccRCC	Clear cell RCC	CC-e.3
TCGA-A3-A8OV-01A-11R-A37O-07	KIRC	98	0	2	0,0006	intermediate	ccRCC	Clear cell RCC	CC-e.2
TCGA-DV-5575-01A-01R-1541-07	KIRC	95	0	5	0,0001	intermediate	ccRCC	Clear cell RCC	CC-e.2
TCGA-CJ-4878-01A-01R-1305-07	KIRC	100	0	0	0,0011	intermediate	ccRCC	Clear cell RCC	CC-e.2
TCGA-CJ-6028-01A-11R-1672-07	KIRC	99	1	0	0,0001	intermediate	ccRCC	Clear cell RCC	CC-e.3
TCGA-B0-5690-01A-11R-1541-07	KIRC	100	0	0	0,0001	intermediate	ccRCC	Clear cell RCC	CC-e.2
TCGA-A3-3363-01A-01R-0864-07	KIRC	0	0	100	0,0006	good	ccRCC	Clear cell RCC	P-e.1a
TCGA-BP-4762-01A-02R-1289-07	KIRC	93	0	7	0,0002	intermediate	ccRCC	Clear cell RCC	CC-e.2
TCGA-CZ-5460-01A-01R-1503-07	KIRC	98	0	2	0,0002	intermediate	ccRCC	Clear cell RCC	CC-e.1
TCGA-BP-4163-01A-02R-1325-07	KIRC	80	0	20	0,0044	intermediate	ccRCC	Clear cell RCC	CC-e.2

TCGA-CJ-6030-01A-11R-1672-07	KIRC	92	0	8	0,0001	intermediate	ccRCC	Clear cell RCC	CC-e.1
TCGA-B8-A7U6-01A-12R-A37O-07	KIRC	100	0	0	0,003	intermediate	ccRCC	Clear cell RCC	CC-e.2
TCGA-BP-5182-01A-01R-1426-07	KIRC	77	2	21	0,0001	intermediate	ccRCC	Clear cell RCC	CC-e.2
TCGA-BP-4961-01A-01R-1334-07	KIRC	100	0	0	0,0001	intermediate	ccRCC	Clear cell RCC	CC-e.2
TCGA-CJ-5679-01A-11R-1541-07	KIRC	95	0	5	0,0009	intermediate	ccRCC	Clear cell RCC	CC-e.3
TCGA-BP-4782-01A-02R-1420-07	KIRC	86	0	14	0,0001	intermediate	ccRCC	Clear cell RCC	CC-e.2
TCGA-BP-5195-01A-02R-1426-07	KIRC	100	0	0	0,0001	intermediate	ccRCC	Clear cell RCC	CC-e.2
TCGA-BP-4340-01A-01R-1289-07	KIRC	100	0	0	0,0097	intermediate	ccRCC	Clear cell RCC	CC-e.2
TCGA-CJ-5676-01A-11R-1541-07	KIRC	100	0	0	0,001	intermediate	ccRCC	Clear cell RCC	CC-e.3
TCGA-CJ-5684-01A-11R-1541-07	KIRC	98	0	2	0,0001	intermediate	ccRCC	Clear cell RCC	CC-e.2
TCGA-BP-4985-01A-01R-1334-07	KIRC	99	1	0	0,0001	intermediate	ccRCC	Clear cell RCC	CC-e.3
TCGA-B8-4153-01B-11R-1672-07	KIRC	97	0	3	0,0001	intermediate	ccRCC	Clear cell RCC	CC-e.2
TCGA-BP-5004-01A-01R-1334-07	KIRC	100	0	0	0,0019	intermediate	ccRCC	Clear cell RCC	CC-e.3
TCGA-CW-5583-01A-02R-1541-07	KIRC	94	0	6	0,0009	intermediate	ccRCC	Clear cell RCC	CC-e.2
TCGA-B0-5075-01A-01R-1334-07	KIRC	88	1	11	0,0001	intermediate	ccRCC	Clear cell RCC	CC-e.1
TCGA-A3-3383-01A-02R-1325-07	KIRC	100	0	0	0,0024	intermediate	ccRCC	Clear cell RCC	CC-e.2
TCGA-BP-5184-01A-01R-1426-07	KIRC	100	0	0	0,0027	intermediate	ccRCC	Clear cell RCC	CC-e.2
TCGA-BP-5189-01A-02R-1426-07	KIRC	100	0	0	0,0001	intermediate	ccRCC	Clear cell RCC	CC-e.2
TCGA-CW-5588-01A-01R-1541-07	KIRC	100	0	0	0,0008	intermediate	ccRCC	Clear cell RCC	CC-e.3
TCGA-BP-4789-01A-01R-1305-07	KIRC	100	0	0	0,0001	intermediate	ccRCC	Clear cell RCC	CC-e.2
TCGA-B0-4712-01A-01R-1503-07	KIRC	99	1	0	0,0017	intermediate	ccRCC	Clear cell RCC	CC-e.3
TCGA-CW-5590-01A-01R-1541-07	KIRC	100	0	0	0,0001	intermediate	ccRCC	Clear cell RCC	CC-e.1
TCGA-B0-5104-01A-01R-1420-07	KIRC	95	5	0	0,0018	intermediate	ccRCC	Clear cell RCC	CC-e.2
TCGA-BP-4763-01A-01R-1289-07	KIRC	100	0	0	0,0001	intermediate	ccRCC	Clear cell RCC	CC-e.3
TCGA-AK-3425-01A-02R-1277-07	KIRC	94	1	5	0,0001	intermediate	ccRCC	Clear cell RCC	CC-e.3
TCGA-BP-4329-01A-02R-1289-07	KIRC	100	0	0	0,0003	intermediate	ccRCC	Clear cell RCC	CC-e.2
TCGA-CW-6088-01A-11R-1672-07	KIRC	100	0	0	0,0001	intermediate	ccRCC	Clear cell RCC	CC-e.2
TCGA-CW-6097-01A-11R-1672-07	KIRC	97	0	3	0,0001	intermediate			CC-e.1
TCGA-A3-3357-01A-02R-1420-07	KIRC	71	1	28	0,0003	intermediate	ccRCC	Clear cell RCC	CC-e.1
TCGA-CJ-4871-01A-01R-1305-07	KIRC	100	0	0	0,0001	intermediate	ccRCC	Clear cell RCC	CC-e.1
TCGA-CJ-5686-01A-11R-1672-07	KIRC	92	0	8	0,0001	intermediate	ccRCC	Clear cell RCC	CC-e.2
TCGA-BP-4801-01A-02R-1420-07	KIRC	99	0	1	0,0001	intermediate	ccRCC	Clear cell RCC	CC-e.2
TCGA-CZ-5987-01A-11R-1672-07	KIRC	86	0	14	0,0002	intermediate	ccRCC	Clear cell RCC	CC-e.1
TCGA-CJ-5682-01A-11R-1541-07	KIRC	100	0	0	0,0002	intermediate	ccRCC	Clear cell RCC	CC-e.3
TCGA-BP-5190-01A-01R-1426-07	KIRC	98	0	2	0,0012	intermediate	ccRCC	Clear cell RCC	CC-e.2
TCGA-A3-A6NJ-01A-12R-A33J-07	KIRC	98	0	2	0,0001	intermediate	ccRCC	Clear cell RCC	CC-e.2
TCGA-B4-5378-01A-01R-1503-07	KIRC	95	0	5	0,0001	intermediate			mixed
TCGA-BP-4807-01A-01R-1305-07	KIRC	92	0	8	0,0001	intermediate	ccRCC	Clear cell RCC	CC-e.2
TCGA-CJ-5677-01A-11R-1541-07	KIRC	89	0	11	0,0086	intermediate	ccRCC	Clear cell RCC	CC-e.1
TCGA-BP-5180-01A-01R-1426-07	KIRC	86	0	14	0,0001	intermediate	ccRCC	Clear cell RCC	CC-e.1
TCGA-BP-4159-01A-02R-1289-07	KIRC	100	0	0	0,0001	intermediate	ccRCC	Clear cell RCC	CC-e.2
TCGA-DV-5566-01A-01R-1541-07	KIRC	89	1	10	0,0042	intermediate	ccRCC	Clear cell RCC	CC-e.2
TCGA-BP-5198-01A-01R-1426-07	KIRC	100	0	0	0,0006	intermediate	ccRCC	Clear cell RCC	CC-e.3
TCGA-B8-5163-01A-01R-1420-07	KIRC	98	0	2	0,0001	intermediate	ccRCC	Clear cell RCC	CC-e.3

TCGA-B0-5812-01A-11R-1672-07	KIRC	100	0	0	0,0001	intermediate	ccRCC	Clear cell RCC	CC-e.2
TCGA-BP-4962-01A-01R-1334-07	KIRC	92	1	7	0,0001	intermediate	ccRCC	Clear cell RCC	CC-e.1
TCGA-B8-5551-01A-01R-1541-07	KIRC	88	1	11	0,0001	intermediate	ccRCC	Clear cell RCC	CC-e.3
TCGA-B8-4621-01A-01R-1503-07	KIRC	7	0	93	0,0001	good	ccRCC	Clear cell RCC	P-e.1b
TCGA-B0-4848-01A-01R-1277-07	KIRC	95	0	5	0,0001	intermediate	ccRCC	Clear cell RCC	CC-e.1
TCGA-B8-4154-01A-01R-1188-07	KIRC	100	0	0	0,0115	intermediate	ccRCC	Clear cell RCC	CC-e.2
TCGA-BP-5186-01A-01R-1426-07	KIRC	100	0	0	0,0036	intermediate	ccRCC	Clear cell RCC	CC-e.2
TCGA-CW-5580-01A-01R-1672-07	KIRC	100	0	0	0,0001	intermediate	ccRCC	Clear cell RCC	CC-e.1
TCGA-CZ-5985-01A-11R-1672-07	KIRC	72	1	27	0,0001	intermediate	ccRCC	Clear cell RCC	CC-e.1
TCGA-B2-5641-01A-01R-1541-07	KIRC	87	0	13	0,0001	intermediate	ccRCC	Clear cell RCC	CC-e.1
TCGA-CJ-4640-01A-02R-1325-07	KIRC	61	0	39	0,0031	poor	ccRCC	Clear cell RCC	CC-e.1
TCGA-BP-5178-01A-01R-1426-07	KIRC	98	0	2	0,0031	intermediate	ccRCC	Clear cell RCC	CC-e.3
TCGA-B0-5692-01A-11R-1541-07	KIRC	87	0	13	0,0018	intermediate	ccRCC	Clear cell RCC	CC-e.2
TCGA-AS-3777-01A-01R-0864-07	KIRC	9	85	6	0,0634	good	ChRCC		Ch-e
TCGA-B2-5636-01A-02R-1541-07	KIRC	90	0	10	0,0001	intermediate	ccRCC	Clear cell RCC	mixed
TCGA-B0-4688-01A-01R-1277-07	KIRC	95	0	5	0,063	intermediate	ChRCC	Metabolically Divergent (MD-)ChRCC	CC-e.3
TCGA-DV-A4VZ-01A-11R-A266-07	KIRC	71	3	26	0,0014	intermediate	ccRCC	Clear cell RCC	mixed
TCGA-B0-5707-01A-11R-1541-07	KIRC	0	0	100	0,0015	good	ccRCC	Clear cell RCC	P-e.1a
TCGA-BP-4797-01A-01R-1305-07	KIRC	93	1	6	0,0001	intermediate	ccRCC	Clear cell RCC	CC-e.1
TCGA-GK-A6C7-01A-11R-A33J-07	KIRC	100	0	0	0,0001	intermediate	ccRCC	Clear cell RCC	CC-e.2
TCGA-BP-4784-01A-01R-1305-07	KIRC	93	1	6	0,0001	intermediate			mixed
TCGA-CJ-4869-01A-02R-1426-07	KIRC	79	1	20	0,0029	intermediate	ccRCC	Clear cell RCC	CC-e.3
TCGA-BP-4975-01A-01R-1334-07	KIRC	100	0	0	0,0001	intermediate	ccRCC	Clear cell RCC	CC-e.2
TCGA-CJ-6027-01A-11R-1672-07	KIRC	98	0	2	0,0001	intermediate	ccRCC	Clear cell RCC	CC-e.3
TCGA-B0-4827-01A-02R-1420-07	KIRC	93	1	6	0,0006	intermediate	ccRCC	Clear cell RCC	CC-e.3
TCGA-B0-5710-01A-11R-1672-07	KIRC	100	0	0	0,0001	intermediate	ccRCC	Clear cell RCC	CC-e.2
TCGA-BP-4759-01A-01R-1289-07	KIRC	100	0	0	0,0001	intermediate	ccRCC	Clear cell RCC	CC-e.2
TCGA-BP-5006-01A-01R-1334-07	KIRC	100	0	0	0,0017	intermediate	ccRCC	Clear cell RCC	CC-e.2
TCGA-BP-4177-01A-02R-1420-07	KIRC	75	1	24	0,0001	intermediate			mixed
TCGA-CZ-5455-01A-01R-1503-07	KIRC	99	0	1	0,0001	intermediate	ccRCC	Clear cell RCC	CC-e.2
TCGA-CJ-5678-01A-11R-1541-07	KIRC	98	0	2	0,0006	intermediate	ccRCC	Clear cell RCC	CC-e.1
TCGA-BP-4170-01A-02R-1289-07	KIRC	100	0	0	0,0001	intermediate	ccRCC	Clear cell RCC	CC-e.2
TCGA-CJ-4876-01A-01R-1305-07	KIRC	96	0	4	0,0013	intermediate	ccRCC	Clear cell RCC	CC-e.3
TCGA-BP-4983-01A-01R-1334-07	KIRC	99	1	0	0,003	intermediate	ccRCC	Clear cell RCC	CC-e.3
TCGA-B0-5107-01A-01R-1420-07	KIRC	56	1	43	0,0701	poor	ccRCC	Clear cell RCC	CC-e.3
TCGA-CZ-5456-01A-01R-1503-07	KIRC	100	0	0	0,0001	intermediate	ccRCC	Clear cell RCC	CC-e.3
TCGA-AK-3431-01A-02R-1277-07	KIRC	100	0	0	0,0001	intermediate	ccRCC	Clear cell RCC	CC-e.3
TCGA-B8-5550-01A-01R-1541-07	KIRC	98	0	2	0,0001	intermediate	ccRCC	Clear cell RCC	CC-e.1
TCGA-EU-5906-01A-11R-1672-07	KIRC	100	0	0	0,0002	intermediate	ccRCC	Clear cell RCC	CC-e.2
TCGA-B0-5703-01A-11R-1541-07	KIRC	100	0	0	0,0015	intermediate	ccRCC	Clear cell RCC	CC-e.1
TCGA-DV-5569-01A-01R-1541-07	KIRC	93	1	6	0,0001	intermediate	ccRCC	Clear cell RCC	CC-e.2
TCGA-BP-5194-01A-02R-1426-07	KIRC	100	0	0	0,0002	intermediate	ccRCC	Clear cell RCC	CC-e.2
TCGA-B0-5083-01A-02R-1420-07	KIRC	3	96	1	0,0001	good	ChRCC		mixed
TCGA-B0-4823-01A-02R-1420-07	KIRC	96	0	4	0,0001	intermediate	ccRCC	Clear cell RCC	CC-e.2

TCGA-CZ-4853-01A-01R-1426-07	KIRC	100	0	0	0,0001	intermediate	ccRCC	Clear cell RCC	CC-e.1
TCGA-BP-4164-01A-02R-1325-07	KIRC	100	0	0	0,0006	intermediate	ccRCC	Clear cell RCC	CC-e.1
TCGA-BP-5173-01A-01R-1426-07	KIRC	100	0	0	0,0001	intermediate	ccRCC	Clear cell RCC	CC-e.1
TCGA-B0-5701-01A-11R-1541-07	KIRC	98	0	2	0,0001	intermediate	ccRCC	Clear cell RCC	CC-e.1
TCGA-B0-5117-01A-01R-1420-07	KIRC	0	100	0	0,0001	good	ChRCC		Ch-e
TCGA-B4-5843-01A-11R-1672-07	KIRC	90	0	10	0,0001	intermediate			CC-e.2
TCGA-BP-5170-01A-01R-1426-07	KIRC	98	0	2	0,0001	intermediate	ccRCC	Clear cell RCC	CC-e.3
TCGA-BP-4760-01A-02R-1420-07	KIRC	80	1	19	0,0001	intermediate			mixed
TCGA-B0-4842-01A-02R-1420-07	KIRC	86	1	13	0,0065	intermediate	ccRCC	Clear cell RCC	CC-e.3
TCGA-CZ-5461-01A-01R-1503-07	KIRC	100	0	0	0,0001	intermediate	ccRCC	Clear cell RCC	CC-e.1
TCGA-BP-4343-01A-02R-1289-07	KIRC	100	0	0	0,0001	intermediate	ccRCC	Clear cell RCC	CC-e.3
TCGA-B0-5119-01A-02R-1420-07	KIRC	100	0	0	0,0009	intermediate	ccRCC	Clear cell RCC	CC-e.2
TCGA-BP-4160-01A-02R-1289-07	KIRC	84	1	15	0,0001	intermediate	ccRCC	Clear cell RCC	CC-e.1
TCGA-CZ-5465-01A-01R-1503-07	KIRC	100	0	0	0,0001	intermediate	ccRCC	Clear cell RCC	CC-e.1
TCGA-B0-5699-01A-11R-1541-07	KIRC	98	0	2	0,0009	intermediate	ccRCC	Clear cell RCC	CC-e.2
TCGA-EU-5904-01A-11R-1672-07	KIRC	100	0	0	0,0018	intermediate	ccRCC	Clear cell RCC	CC-e.2
TCGA-CZ-4856-01A-02R-1426-07	KIRC	97	0	3	0,0001	intermediate	ccRCC	Clear cell RCC	CC-e.2
TCGA-CW-5591-11A-01R-1541-07	KIRC	100	0	0	0,0001	intermediate	ccRCC	Clear cell RCC	CC-e.1
TCGA-B8-5549-01A-01R-1541-07	KIRC	100	0	0	0,0001	intermediate	ccRCC	Clear cell RCC	CC-e.2
TCGA-CZ-5988-01A-11R-1672-07	KIRC	97	0	3	0,0001	intermediate	ccRCC	Clear cell RCC	CC-e.2
TCGA-B0-4839-01A-01R-1305-07	KIRC	90	0	10	0,0011	intermediate	ccRCC	Clear cell RCC	CC-e.2
TCGA-B0-5702-01A-11R-1541-07	KIRC	38	5	57	0,011	poor	ccRCC	Clear cell RCC	CC-e.3
TCGA-A3-3306-01A-01R-0864-07	KIRC	100	0	0	0,0003	intermediate			CC-e.1
TCGA-CZ-4866-01A-01R-1503-07	KIRC	81	1	18	0,0053	intermediate	ccRCC	Clear cell RCC	CC-e.1
TCGA-AK-3450-01A-02R-1277-07	KIRC	98	0	2	0,0116	intermediate	ccRCC	Clear cell RCC	CC-e.2
TCGA-B0-5711-01A-11R-1672-07	KIRC	100	0	0	0,0001	intermediate	ccRCC	Clear cell RCC	CC-e.2
TCGA-CJ-5681-01A-11R-1541-07	KIRC	89	2	9	0,0043	intermediate	ccRCC	Clear cell RCC	P-e.2
TCGA-BP-5181-01A-01R-1426-07	KIRC	96	0	4	0,0001	intermediate	ccRCC	Clear cell RCC	CC-e.2
TCGA-CW-5584-01A-01R-1541-07	KIRC	100	0	0	0,0002	intermediate	ccRCC	Clear cell RCC	CC-e.2
TCGA-BP-5200-01A-01R-1426-07	KIRC	100	0	0	0,0001	intermediate	ccRCC	Clear cell RCC	CC-e.3
TCGA-AK-3436-01A-02R-1325-07	KIRC	100	0	0	0,0021	intermediate	ccRCC	Clear cell RCC	CC-e.3
TCGA-B8-5553-01A-01R-1541-07	KIRC	95	0	5	0,0002	intermediate	ccRCC	Clear cell RCC	CC-e.2
TCGA-CW-5587-01A-01R-1541-07	KIRC	100	0	0	0,0085	intermediate	ccRCC	Clear cell RCC	CC-e.2
TCGA-CJ-4893-01A-01R-1305-07	KIRC	100	0	0	0,0008	intermediate	ccRCC	Clear cell RCC	CC-e.2
TCGA-B0-5693-01A-11R-1541-07	KIRC	100	0	0	0,001	intermediate	ccRCC	Clear cell RCC	CC-e.2
TCGA-CJ-4905-01A-02R-1426-07	KIRC	100	0	0	0,0001	intermediate	ccRCC	Clear cell RCC	CC-e.2
TCGA-CZ-5452-01A-01R-1503-07	KIRC	93	0	7	0,0003	intermediate	ccRCC	Clear cell RCC	CC-e.1
TCGA-B0-5116-01A-02R-1420-07	KIRC	100	0	0	0,0001	intermediate	ccRCC	Clear cell RCC	CC-e.1
TCGA-AK-3456-01A-02R-1325-07	KIRC	15	1	84	0,0002	intermediate	ccRCC	Clear cell RCC	P-e.2
TCGA-CJ-4897-01A-03R-1426-07	KIRC	96	0	4	0,0001	intermediate	ccRCC	Clear cell RCC	CC-e.2
TCGA-B0-5712-01A-11R-1672-07	KIRC	93	0	7	0,0052	intermediate	ccRCC	Clear cell RCC	CC-e.2
TCGA-BP-4174-01A-02R-1289-07	KIRC	100	0	0	0,0042	intermediate	ccRCC	Clear cell RCC	CC-e.1
TCGA-CZ-5462-01A-01R-1503-07	KIRC	60	1	39	0,0001	poor	ccRCC	Clear cell RCC	CC-e.3
TCGA-BP-4761-01A-01R-1289-07	KIRC	79	0	21	0,0282	intermediate	ccRCC	Clear cell RCC	CC-e.3

TCGA-CZ-5986-01A-11R-1672-07	KIRC	100	0	0	0,0001	intermediate	ccRCC	Clear cell RCC	CC-e.2
TCGA-CW-5581-01A-02R-1541-07	KIRC	99	1	0	0,0008	intermediate	ccRCC	Clear cell RCC	CC-e.2
TCGA-B4-5834-01A-11R-1672-07	KIRC	100	0	0	0,0001	intermediate			CC-e.2
TCGA-CZ-5468-01A-01R-1503-07	KIRC	100	0	0	0,003	intermediate	ccRCC	Clear cell RCC	CC-e.3
TCGA-B8-A54E-01A-11R-A266-07	KIRC	51	12	37	0,358	poor	ccRCC	Clear cell RCC	P-e.2
TCGA-B8-4619-01A-02R-1325-07	KIRC	8	88	4	0,0038	good			Ch-e
TCGA-DV-5567-01A-01R-1541-07	KIRC	77	2	21	0,0001	intermediate	ccRCC	Clear cell RCC	mixed
TCGA-CZ-5451-01A-01R-1503-07	KIRC	100	0	0	0,0011	intermediate	ccRCC	Clear cell RCC	CC-e.2
TCGA-B0-5700-01A-11R-1541-07	KIRC	100	0	0	0,0016	intermediate	ccRCC	Clear cell RCC	CC-e.2
TCGA-CJ-4874-01A-01R-1305-07	KIRC	100	0	0	0,0001	intermediate	ccRCC	Clear cell RCC	CC-e.2
TCGA-CZ-5457-01A-01R-1503-07	KIRC	100	0	0	0,0017	intermediate	ccRCC	Clear cell RCC	CC-e.2
TCGA-B4-5836-01A-11R-1672-07	KIRC	100	0	0	0,0004	intermediate			CC-e.2
TCGA-B8-5546-01A-01R-1541-07	KIRC	38	11	51	0,014	poor	ccRCC	Clear cell RCC	P-e.2
TCGA-A3-3385-01A-02R-1420-07	KIRC	100	0	0	0,0107	intermediate	ccRCC	Clear cell RCC	CC-e.2
TCGA-B0-5096-01A-01R-1420-07	KIRC	100	0	0	0,0003	intermediate	ccRCC	Clear cell RCC	CC-e.3
TCGA-AK-3429-01A-02R-1325-07	KIRC	100	0	0	0,0001	intermediate	ccRCC	Clear cell RCC	CC-e.2
TCGA-BP-5176-01A-01R-1426-07	KIRC	96	0	4	0,0018	intermediate	ccRCC	Clear cell RCC	CC-e.2
TCGA-BP-5174-01A-01R-1426-07	KIRC	86	0	14	0,0088	intermediate	ccRCC	Clear cell RCC	CC-e.2
TCGA-B0-5695-01A-11R-1541-07	KIRC	99	1	0	0,0002	intermediate	ccRCC	Clear cell RCC	CC-e.2
TCGA-CW-6093-01A-11R-1672-07	KIRC	100	0	0	0,0001	intermediate	ccRCC	Clear cell RCC	CC-e.2
TCGA-CZ-5453-01A-01R-1503-07	KIRC	100	0	0	0,0001	intermediate	ccRCC	Clear cell RCC	CC-e.2
TCGA-BP-5202-01A-02R-1426-07	KIRC	100	0	0	0,0034	intermediate	ccRCC	Clear cell RCC	CC-e.2
TCGA-CJ-4904-01A-02R-1426-07	KIRC	100	0	0	0,0001	intermediate	ccRCC	Clear cell RCC	CC-e.2
TCGA-BP-4959-01A-01R-1334-07	KIRC	84	0	16	0,0001	intermediate	ccRCC	Clear cell RCC	CC-e.1
TCGA-BP-4799-01A-01R-1305-07	KIRC	100	0	0	0,0001	intermediate	ccRCC	Clear cell RCC	CC-e.3
TCGA-G6-A5PC-01A-11R-A33J-07	KIRC	69	1	30	0,0202	poor	ccRCC	Clear cell RCC	CC-e.1
TCGA-BP-4770-01A-01R-1503-07	KIRC	99	1	0	0,0005	intermediate	ccRCC	Clear cell RCC	CC-e.3
TCGA-BP-4334-01A-01R-1289-07	KIRC	0	100	0	0,0024	good	ChRCC		Ch-e
TCGA-B0-5713-01A-11R-1672-07	KIRC	100	0	0	0,0001	intermediate	ccRCC	Clear cell RCC	CC-e.2
TCGA-B0-5696-01A-11R-1541-07	KIRC	100	0	0	0,0011	intermediate	ccRCC	Clear cell RCC	CC-e.2
TCGA-CJ-4644-01A-02R-1325-07	KIRC	100	0	0	0,0001	intermediate	ccRCC	Clear cell RCC	CC-e.2
TCGA-CJ-5675-01A-11R-1541-07	KIRC	92	0	8	0,0027	intermediate	ccRCC	Clear cell RCC	CC-e.1
TCGA-BP-5192-01A-01R-1426-07	KIRC	95	0	5	0,0046	intermediate	ccRCC	Clear cell RCC	CC-e.2
TCGA-A3-3373-01A-02R-1420-07	KIRC	100	0	0	0,0088	intermediate	ccRCC	Clear cell RCC	CC-e.2
TCGA-CJ-5680-01A-11R-1541-07	KIRC	64	1	35	0,0019	poor	ccRCC	Clear cell RCC	CC-e.1
TCGA-DV-5565-01A-01R-1541-07	KIRC	93	0	7	0,0001	intermediate	ccRCC	Clear cell RCC	CC-e.1
TCGA-B8-5164-01A-01R-1420-07	KIRC	92	0	8	0,0001	intermediate	ccRCC	Clear cell RCC	CC-e.2
TCGA-CJ-6032-01A-11R-1672-07	KIRC	98	0	2	0,0001	intermediate	ccRCC	Clear cell RCC	CC-e.2
TCGA-B4-5377-01A-01R-1503-07	KIRC	97	0	3	0,001	intermediate	ccRCC	Clear cell RCC	CC-e.2
TCGA-B8-5159-01A-01R-1420-07	KIRC	97	0	3	0,0146	intermediate	ccRCC	Clear cell RCC	CC-e.2
TCGA-B0-5698-01A-11R-1672-07	KIRC	100	0	0	0,0001	intermediate	ccRCC	Clear cell RCC	CC-e.2
TCGA-CW-6090-01A-11R-1672-07	KIRC	100	0	0	0,0001	intermediate	ccRCC	Clear cell RCC	CC-e.1
TCGA-BP-5168-01A-01R-1420-07	KIRC	100	0	0	0,0001	intermediate	ccRCC	Clear cell RCC	CC-e.2
TCGA-A3-3374-01A-02R-1325-07	KIRC	0	100	0	0,0001	good	ChRCC		Ch-e

TCGA-B4-5838-01A-11R-1672-07	KIRC	98	0	2	0,0002	intermediate			CC-e.1
TCGA-CJ-4870-01A-01R-1305-07	KIRC	99	0	1	0,0012	intermediate	ccRCC	Clear cell RCC	CC-e.2
TCGA-B4-5844-01A-11R-1672-07	KIRC	100	0	0	0,0001	intermediate			CC-e.2
TCGA-CZ-5463-01A-01R-1503-07	KIRC	100	0	0	0,0001	intermediate	ccRCC	Clear cell RCC	CC-e.2
TCGA-B0-5705-01A-11R-1541-07	KIRC	99	0	1	0,0043	intermediate	ccRCC	Clear cell RCC	CC-e.2
TCGA-B0-4696-01A-01R-1277-07	KIRC	96	2	2	0,0009	intermediate	ChRCC	Metabolically Divergent (MD-)ChRCC	CC-e.3
TCGA-BP-4976-01A-01R-1334-07	KIRC	95	0	5	0,0016	intermediate	ccRCC	Clear cell RCC	CC-e.2
TCGA-CW-6087-01A-11R-1672-07	KIRC	33	1	66	0,0066	poor	ccRCC	Clear cell RCC	CC-e.3
TCGA-CJ-4885-01A-01R-1305-07	KIRC	97	0	3	0,0015	intermediate	ccRCC	Clear cell RCC	CC-e.2
TCGA-BP-4338-01A-01R-1289-07	KIRC	91	0	9	0,0001	intermediate	ccRCC	Clear cell RCC	CC-e.3
TCGA-CZ-5454-01A-01R-1503-07	KIRC	99	1	0	0,0001	intermediate	ccRCC	Clear cell RCC	CC-e.2
TCGA-CZ-5467-01A-01R-1503-07	KIRC	100	0	0	0,0001	intermediate	ccRCC	Clear cell RCC	CC-e.2
TCGA-AK-3428-01A-02R-1277-07	KIRC	100	0	0	0,001	intermediate	ccRCC	Clear cell RCC	CC-e.2
TCGA-CW-5585-01A-01R-1541-07	KIRC	100	0	0	0,002	intermediate	ccRCC	Clear cell RCC	CC-e.2
TCGA-T7-A92I-01A-11R-A37O-07	KIRC	40	2	58	0,0049	poor	ccRCC	Clear cell RCC	P-e.2
TCGA-B4-5835-01A-11R-1672-07	KIRC	54	3	43	0,0621	poor			P-e.2
TCGA-AK-3451-01A-02R-1188-07	KIRC	79	1	20	0,0058	intermediate	ccRCC	Clear cell RCC	CC-e.2
TCGA-CZ-4860-01A-01R-1305-07	KIRC	99	1	0	0,067	intermediate			CC-e.3
TCGA-CZ-5982-01A-11R-1672-07	KIRC	100	0	0	0,0008	intermediate	ccRCC	Clear cell RCC	CC-e.2
TCGA-B0-4698-01A-01R-1503-07	KIRC	99	1	0	0,154	intermediate			CC-e.3
TCGA-CZ-4859-01A-02R-1426-07	KIRC	96	0	4	0,0048	intermediate	ccRCC	Clear cell RCC	CC-e.2
TCGA-EU-5907-01A-11R-1672-07	KIRC	100	0	0	0,0001	intermediate	ccRCC	Clear cell RCC	CC-e.1
TCGA-B8-4151-01A-01R-1188-07	KIRC	100	0	0	0,0084	intermediate	ccRCC	Clear cell RCC	CC-e.2
TCGA-AK-3433-01A-02R-1277-07	KIRC	0	100	0	0,001	good	ChRCC		Ch-e
TCGA-B0-4834-01A-01R-1305-07	KIRC	10	90	0	0,0001	good			Ch-e
TCGA-B8-4146-01B-11R-1672-07	KIRC	100	0	0	0,0002	intermediate	ccRCC	Clear cell RCC	CC-e.2
TCGA-A3-3328-01A-01R-0864-07	KIRC	0	100	0	0,0001	good			Ch-e
TCGA-AK-3427-01A-01R-0864-07	KIRC	3	97	0	0,0001	good			Ch-e
TCGA-AK-3440-01A-02R-1277-07	KIRC	0	100	0	0,0219	good	ChRCC		Ch-e
TCGA-B4-5832-01A-11R-1672-07	KIRC	91	0	9	0,0028	intermediate			CC-e.3
TCGA-CZ-5989-01A-11R-1672-07	KIRC	99	0	1	0,0001	intermediate	ccRCC	Clear cell RCC	CC-e.2
TCGA-AK-3443-01A-02R-1325-07	KIRC	0	100	0	0,0003	good			Ch-e
TCGA-AK-3447-01A-01R-1766-07	KIRC	0	100	0	0,0043	good	ChRCC		Ch-e
TCGA-B9-A8YH-01A-11R-A37K-07	KIRP	1	0	99	0,0001	good	PRCC T1	Type 1 Papillary RCC	P-e.1a
TCGA-A4-A4ZT-01A-11R-A26U-07	KIRP	0	0	100	0,0001	good	PRCC T1	Type 1 Papillary RCC	P-e.1a
TCGA-BQ-7051-01A-12R-1965-07	KIRP	0	0	100	0,0001	good	PRCC T1	Type 1 Papillary RCC	P-e.1a
TCGA-G7-7501-01A-11R-2204-07	KIRP	26	1	73	0,0001	poor	PRCC T2	Type 2 Papillary RCC	P-e.2
TCGA-2Z-A9J6-01A-11R-A38C-07	KIRP	0	0	100	0,0001	good	PRCC T1	Type 1 Papillary RCC	P-e.1b
TCGA-IA-A83S-01A-11R-A355-07	KIRP	0	0	100	0,0001	good	PRCC T1	Type 1 Papillary RCC	P-e.1b
TCGA-2Z-A9J9-01A-11R-A42S-07	KIRP	69	2	29	0,0001	poor			mixed
TCGA-F9-A7VF-01A-11R-A33Z-07	KIRP	0	0	100	0,004	good	PRCC T1	Type 1 Papillary RCC	P-e.1a
TCGA-2Z-A9J8-01A-11R-A42S-07	KIRP	0	0	100	0,002	good	PRCC T1	Type 1 Papillary RCC	P-e.1a
TCGA-G7-6792-01A-21R-1965-07	KIRP	0	0	100	0,0002	good	PRCC T1	Type 1 Papillary RCC	P-e.1a
TCGA-Y8-A896-01A-11R-A36F-07	KIRP	9	1	90	0,0001	good	PRCC Unc	Unclassified Papillary RCC	P.CIMP-e

TCGA-Y8-A8RZ-01A-11R-A37K-07	KIRP	1	0	99	0,0001	good	PRCC T1	Type 1 Papillary RCC	P-e.1a
TCGA-2Z-A9JT-01A-11R-A42S-07	KIRP	0	0	100	0,0002	good	PRCC T1	Type 1 Papillary RCC	P-e.1b
TCGA-SX-A71U-01A-12R-A33Z-07	KIRP	0	0	100	0,0001	good	PRCC T1	Type 1 Papillary RCC	P-e.1b
TCGA-IA-A83T-01A-11R-A355-07	KIRP	0	0	100	0,0001	good	PRCC T1	Type 1 Papillary RCC	P-e.1b
TCGA-UZ-A9PL-01A-11R-A38C-07	KIRP	0	0	100	0,0001	good	PRCC T1	Type 1 Papillary RCC	P-e.1a
TCGA-A4-A5DU-01A-11R-A28H-07	KIRP	0	0	100	0,0001	good	PRCC T1	Type 1 Papillary RCC	P-e.1a
TCGA-KV-A6GE-01A-11R-A31O-07	KIRP	0	0	100	0,0002	good	PRCC T1	Type 1 Papillary RCC	P-e.1a
TCGA-SX-A7SR-01A-12R-A36F-07	KIRP	0	0	100	0,0001	good	PRCC T1	Type 1 Papillary RCC	P-e.1b
TCGA-2Z-A9JR-01A-12R-A42S-07	KIRP	0	0	100	0,0001	good	PRCC T1	Type 1 Papillary RCC	P-e.1b
TCGA-A4-A5XZ-01A-11R-A31O-07	KIRP	0	0	100	0,001	good	PRCC T1	Type 1 Papillary RCC	P-e.1a
TCGA-SX-A71V-01A-11R-A33Z-07	KIRP	0	0	100	0,0001	good	PRCC T1	Type 1 Papillary RCC	P-e.1a
TCGA-A4-7287-01A-11R-2139-07	KIRP	54	13	33	0,0017	poor			CC-e.3
TCGA-BQ-5881-01A-11R-1592-07	KIRP	0	0	100	0,0001	good	PRCC T2	Type 2 Papillary RCC	P-e.1b
TCGA-F9-A97G-01A-11R-A38C-07	KIRP	32	5	63	0,0068	poor	PRCC T2	Type 2 Papillary RCC	P-e.2
TCGA-BQ-7062-01A-11R-1965-07	KIRP	0	0	100	0,0001	good	PRCC T1	Type 1 Papillary RCC	P-e.1b
TCGA-A4-8311-01A-11R-2404-07	KIRP	0	0	100	0,0001	good	PRCC T1	Type 1 Papillary RCC	P-e.1a
TCGA-PJ-A5Z8-01A-11R-A28H-07	KIRP	15	0	85	0,0103	intermediate	PRCC T1	Type 1 Papillary RCC	P-e.1b
TCGA-AL-3468-01A-02R-1351-07	KIRP	1	0	99	0,0001	good			P-e.1a
TCGA-Y8-A895-01A-11R-A36F-07	KIRP	0	0	100	0,0009	good	PRCC T1	Type 1 Papillary RCC	P-e.1a
TCGA-F9-A7Q0-01A-11R-A36F-07	KIRP	5	1	94	0,002	good	PRCC T1	Type 1 Papillary RCC	P-e.1a
TCGA-BQ-5878-01A-11R-1592-07	KIRP	0	0	100	0,0056	good	PRCC T2	Type 2 Papillary RCC	P-e.1a
TCGA-WN-AB4C-01A-11R-A42S-07	KIRP	20	7	73	0,1242	poor	PRCC Unc	Unclassified Papillary RCC	P-e.2
TCGA-UZ-A9Q0-01A-12R-A42S-07	KIRP	0	0	100	0,0002	good	PRCC T1	Type 1 Papillary RCC	P-e.1a
TCGA-A4-7996-01A-11R-2204-07	KIRP	0	0	100	0,0002	good	PRCC T1	Type 1 Papillary RCC	P-e.1b
TCGA-BQ-5879-01A-11R-1592-07	KIRP	54	2	44	0,0161	poor	KIRP CIMP	Unclassified Papillary RCC	P.CIMP-e
TCGA-BQ-5882-01A-11R-1592-07	KIRP	69	4	27	0,0002	poor	PRCC T2	Type 2 Papillary RCC	P-e.2
TCGA-MH-A55Z-01A-11R-A26U-07	KIRP	0	0	100	0,0001	good	PRCC T1	Type 1 Papillary RCC	P-e.1a
TCGA-5P-A9K4-01A-11R-A42S-07	KIRP	4	0	96	0,0051	good	PRCC T1	Type 1 Papillary RCC	P-e.1a
TCGA-UZ-A9PZ-01A-11R-A42S-07	KIRP	0	0	100	0,0002	good	PRCC T1	Type 1 Papillary RCC	P-e.1a
TCGA-A4-A5Y0-01A-11R-A31O-07	KIRP	10	0	90	0,0043	good	PRCC T2	Type 2 Papillary RCC	P-e.1a
TCGA-B1-5398-01A-02R-1592-07	KIRP	5	0	95	0,0001	good	PRCC T2	Type 2 Papillary RCC	P-e.2
TCGA-AL-3466-01A-02R-1351-07	KIRP	21	2	77	0,0001	poor	PRCC T2	Type 2 Papillary RCC	P.CIMP-e
TCGA-HE-A5NF-01A-11R-A26U-07	KIRP	0	0	100	0,0006	good	PRCC T1	Type 1 Papillary RCC	P-e.1b
TCGA-MH-A55W-01A-11R-A26U-07	KIRP	0	0	100	0,0001	good	PRCC Unc	Unclassified Papillary RCC	P-e.1a
TCGA-WN-A9G9-01A-12R-A37K-07	KIRP	61	0	39	0,0203	poor	PRCC T2	Type 2 Papillary RCC	CC-e.3
TCGA-UZ-A9PQ-01A-11R-A42S-07	KIRP	25	1	74	0,0042	poor	PRCC T1	Type 1 Papillary RCC	P-e.2
TCGA-AL-3471-01A-02R-1351-07	KIRP	0	0	100	0,0012	good	PRCC T1	Type 1 Papillary RCC	P-e.1a
TCGA-BQ-7059-01A-11R-1965-07	KIRP	3	1	96	0,0001	good	PRCC T1	Type 1 Papillary RCC	P-e.1b
TCGA-PJ-A5Z9-01A-11R-A28H-07	KIRP	0	0	100	0,0001	good	PRCC T1	Type 1 Papillary RCC	P-e.1a
TCGA-2Z-A9J2-01A-11R-A38C-07	KIRP	3	1	96	0,0002	good	PRCC T2	Type 2 Papillary RCC	P-e.1b
TCGA-5P-A9JZ-01A-11R-A42S-07	KIRP	0	0	100	0,0001	good	PRCC T1	Type 1 Papillary RCC	P-e.1a
TCGA-AL-A5DJ-01A-11R-A26U-07	KIRP	5	1	94	0,0024	good			P.CIMP-e
TCGA-IZ-A6M9-01A-11R-A31O-07	KIRP	0	0	100	0,0005	good	PRCC T1	Type 1 Papillary RCC	P-e.1a
TCGA-SX-A7SM-01A-11R-A355-07	KIRP	6	0	94	0,0002	good	PRCC T2	Type 2 Papillary RCC	P-e.2

TCGA-B9-A5W8-01A-11R-A28H-07	KIRP	0	0	100	0,0001	good	PRCC T1	Type 1 Papillary RCC	P-e.1b
TCGA-A4-A57E-01A-11R-A26U-07	KIRP	64	1	35	0,0014	poor	PRCC T2	Type 2 Papillary RCC	P-e.2
TCGA-BQ-7049-01A-11R-1965-07	KIRP	25	1	74	0,0005	poor			P-e.1a
TCGA-DW-7841-01A-11R-A32Z-07	KIRP	0	0	100	0,0001	good	PRCC T1	Type 1 Papillary RCC	P-e.1b
TCGA-G7-6790-01A-11R-1965-07	KIRP	0	0	100	0,0001	good	PRCC T2	Type 2 Papillary RCC	P-e.1b
TCGA-5P-A9JV-01A-12R-A42S-07	KIRP	70	0	30	0,0011	intermediate	PRCC T1	Type 1 Papillary RCC	CC-e.2
TCGA-G7-A8LB-01A-11R-A36F-07	KIRP	0	0	100	0,0043	good	PRCC T1	Type 1 Papillary RCC	P-e.1a
TCGA-IZ-8196-01A-11R-2404-07	KIRP	0	0	100	0,0001	good	PRCC T1	Type 1 Papillary RCC	P-e.1a
TCGA-V9-A7HT-01A-11R-A33Z-07	KIRP	3	1	96	0,0001	good	PRCC T1	Type 1 Papillary RCC	P-e.1b
TCGA-SX-A71R-01A-12R-A33Z-07	KIRP	0	0	100	0,0001	good	PRCC T1	Type 1 Papillary RCC	P-e.1a
TCGA-B9-4117-01A-02R-1351-07	KIRP	0	0	100	0,0005	good	PRCC Unc	Unclassified Papillary RCC	P-e.1b
TCGA-GL-6846-01A-11R-1965-07	KIRP	0	0	100	0,0001	good	PRCC T1	Type 1 Papillary RCC	P-e.1a
TCGA-DW-7834-01A-11R-2139-07	KIRP	0	0	100	0,0001	good	PRCC T1	Type 1 Papillary RCC	P-e.1a
TCGA-UZ-A9PJ-01A-11R-A38C-07	KIRP	0	0	100	0,0005	good	PRCC T1	Type 1 Papillary RCC	P-e.1a
TCGA-SX-A7SN-01A-11R-A355-07	KIRP	0	0	100	0,0001	good	PRCC T1	Type 1 Papillary RCC	P-e.1b
TCGA-AL-3467-01A-02R-1351-07	KIRP	53	0	47	0,0215	poor			P-e.1a
TCGA-BQ-5880-01A-11R-1592-07	KIRP	27	2	71	0,0014	poor	PRCC T2	Type 2 Papillary RCC	P-e.2
TCGA-G7-6796-01A-11R-1965-07	KIRP	0	0	100	0,0001	good	PRCC T2	Type 2 Papillary RCC	P-e.2
TCGA-UZ-A9PM-01A-21R-A38C-07	KIRP	5	0	95	0,0002	good	PRCC T1	Type 1 Papillary RCC	P-e.1b
TCGA-A4-7734-01A-11R-A32Z-07	KIRP	0	0	100	0,0011	good	PRCC T1	Type 1 Papillary RCC	P-e.1a
TCGA-A4-A7UZ-01A-12R-A355-07	KIRP	6	0	94	0,0026	good	PRCC Unc	Unclassified Papillary RCC	P-e.1a
TCGA-Y8-A894-01A-11R-A36F-07	KIRP	0	0	100	0,0028	good	PRCC T1	Type 1 Papillary RCC	P-e.1a
TCGA-BQ-5877-01A-11R-1592-07	KIRP	43	0	57	0,0014	poor	PRCC Unc	Unclassified Papillary RCC	P.CIMP-e
TCGA-DW-7963-01B-11R-A28H-07	KIRP	0	0	100	0,0001	good	PRCC T1	Type 1 Papillary RCC	P-e.1b
TCGA-IA-A83V-01A-11R-A355-07	KIRP	1	0	99	0,0001	good	PRCC T2	Type 2 Papillary RCC	P-e.1b
TCGA-P4-AAVO-01A-11R-A42S-07	KIRP	0	0	100	0,0001	good			P-e.2
TCGA-2Z-A9JK-01A-11R-A42S-07	KIRP	69	1	30	0,0001	poor	PRCC T2	Type 2 Papillary RCC	CC-e.2
TCGA-Y8-A8RY-01A-11R-A37K-07	KIRP	0	0	100	0,0001	good	PRCC T1	Type 1 Papillary RCC	P-e.1b
TCGA-5P-A9K8-01A-11R-A42S-07	KIRP	43	23	34	0,1297	poor	PRCC T1	Type 1 Papillary RCC	mixed
TCGA-UZ-A9PN-01A-11R-A38C-07	KIRP	24	1	75	0,0001	poor	PRCC Unc	Unclassified Papillary RCC	P-e.1a
TCGA-2Z-A9JE-01A-11R-A42S-07	KIRP	0	0	100	0,0004	good	PRCC T1	Type 1 Papillary RCC	P-e.1a
TCGA-2Z-A9J3-01A-12R-A38C-07	KIRP	0	0	100	0,0016	good	PRCC T1	Type 1 Papillary RCC	P-e.1a
TCGA-UZ-A9PV-01A-11R-A42S-07	KIRP	0	0	100	0,0112	good	PRCC T1	Type 1 Papillary RCC	P-e.1a
TCGA-BQ-7056-01A-11R-1965-07	KIRP	6	0	94	0,0069	good			P-e.1a
TCGA-BQ-7044-01A-11R-1965-07	KIRP	0	0	100	0,0001	good	PRCC T2	Type 2 Papillary RCC	P-e.2
TCGA-5P-A9JW-01A-11R-A42S-07	KIRP	0	0	100	0,0001	good	PRCC T1	Type 1 Papillary RCC	P-e.1b
TCGA-HE-A5NK-01A-11R-A26U-07	KIRP	28	2	70	0,0019	poor			P-e.1b
TCGA-B3-3925-01A-02R-1351-07	KIRP	0	0	100	0,0001	good	PRCC Unc	Unclassified Papillary RCC	P-e.1a
TCGA-B1-A655-01A-11R-A31O-07	KIRP	24	2	74	0,0011	poor	PRCC T2	Type 2 Papillary RCC	P-e.2
TCGA-A4-8518-01A-11R-2404-07	KIRP	0	1	99	0,0004	good	PRCC T2	Type 2 Papillary RCC	P-e.1b
TCGA-SX-A71S-01A-11R-A33Z-07	KIRP	0	0	100	0,0002	good	PRCC T1	Type 1 Papillary RCC	P-e.1a
TCGA-P4-AAVL-01A-11R-A42S-07	KIRP	5	0	95	0,0001	good	PRCC T1	Type 1 Papillary RCC	P-e.1a
TCGA-SX-A7SP-01A-11R-A355-07	KIRP	0	0	100	0,0007	good	PRCC T1	Type 1 Papillary RCC	P-e.1b
TCGA-G7-6797-01A-11R-1965-07	KIRP	0	0	100	0,0061	good	PRCC T1	Type 1 Papillary RCC	P-e.1a

TCGA-B9-4116-01A-02R-1351-07	KIRP	13	0	87	0,0001	intermediate	PRCC T1	Type 1 Papillary RCC	P.CIMP-e
TCGA-IA-A40Y-01A-11R-A24Z-07	KIRP	15	0	85	0,0001	intermediate	PRCC Unc	Unclassified Papillary RCC	P.CIMP-e
TCGA-GL-A4EM-01A-11R-A24Z-07	KIRP	12	3	85	0,001	intermediate	PRCC Unc	Unclassified Papillary RCC	P-e.2
TCGA-A4-8517-01A-11R-2404-07	KIRP	0	0	100	0,0001	good	PRCC Unc	Unclassified Papillary RCC	P-e.1a
TCGA-B9-5155-01A-01R-1592-07	KIRP	0	0	100	0,0079	good	PRCC T1	Type 1 Papillary RCC	P-e.1a
TCGA-5P-A9K3-01A-11R-A42S-07	KIRP	28	2	70	0,0909	poor	PRCC T1	Type 1 Papillary RCC	P.CIMP-e
TCGA-2Z-A9JG-01A-11R-A42S-07	KIRP	0	0	100	0,0001	good	PRCC T1	Type 1 Papillary RCC	P-e.1a
TCGA-BQ-7046-01A-11R-1965-07	KIRP	0	0	100	0,0003	good	PRCC T1	Type 1 Papillary RCC	P-e.1b
TCGA-DW-7842-01A-11R-A32Z-07	KIRP	0	0	100	0,0013	good	PRCC T1	Type 1 Papillary RCC	P-e.1a
TCGA-DW-5561-01A-01R-1592-07	KIRP	0	0	100	0,0001	good	PRCC T1	Type 1 Papillary RCC	P-e.1a
TCGA-SX-A7SL-01A-11R-A355-07	KIRP	0	0	100	0,0039	good	PRCC Unc	Unclassified Papillary RCC	P-e.1a
TCGA-B9-A5W9-01A-11R-A28H-07	KIRP	0	0	100	0,0001	good	PRCC Unc	Unclassified Papillary RCC	P-e.1a
TCGA-BQ-7058-01A-11R-1965-07	KIRP	11	0	89	0,0002	intermediate	PRCC T2	Type 2 Papillary RCC	P-e.2
TCGA-B9-A8YI-01A-21R-A37K-07	KIRP	2	0	98	0,0001	good	PRCC T2	Type 2 Papillary RCC	P-e.2
TCGA-A4-7583-01A-11R-A32Z-07	KIRP	0	0	100	0,0001	good	PRCC T1	Type 1 Papillary RCC	P-e.1b
TCGA-BQ-5888-01A-11R-1592-07	KIRP	0	0	100	0,0001	good	PRCC Unc	Unclassified Papillary RCC	CC-e.3
TCGA-UZ-A9PS-01A-11R-A42S-07	KIRP	0	0	100	0,0001	good	PRCC T1	Type 1 Papillary RCC	P-e.1a
TCGA-MH-A854-01A-11R-A355-07	KIRP	1	0	99	0,0008	good	PRCC T1	Type 1 Papillary RCC	P-e.1a
TCGA-BQ-5891-01A-11R-1592-07	KIRP	14	0	86	0,0002	intermediate	PRCC T2	Type 2 Papillary RCC	CC-e.3
TCGA-BQ-5875-01A-11R-1592-07	KIRP	33	5	62	0,0028	poor	PRCC T2	Type 2 Papillary RCC	P-e.2
TCGA-B1-7332-01A-11R-A32Z-07	KIRP	0	0	100	0,0075	good	PRCC T1	Type 1 Papillary RCC	P-e.1a
TCGA-MH-A857-01A-11R-A355-07	KIRP	8	59	33	0,6587	good			mixed
TCGA-IZ-8195-01A-31R-2404-07	KIRP	7	0	93	0,0024	good	PRCC T2	Type 2 Papillary RCC	P-e.2
TCGA-A4-7997-01A-11R-2204-07	KIRP	0	0	100	0,0001	good	PRCC T1	Type 1 Papillary RCC	P-e.1a
TCGA-A4-A5Y1-01A-11R-A28H-07	KIRP	1	0	99	0,0001	good	PRCC T2	Type 2 Papillary RCC	P.CIMP-e
TCGA-DZ-6134-01A-11R-1965-07	KIRP	5	0	95	0,0005	good	PRCC Unc	Unclassified Papillary RCC	P-e.1b
TCGA-Y8-A8S0-01A-11R-A37K-07	KIRP	0	0	100	0,0009	good	PRCC T1	Type 1 Papillary RCC	P-e.1a
TCGA-5P-A9K0-01A-11R-A42S-07	KIRP	0	0	100	0,0007	good	PRCC T1	Type 1 Papillary RCC	P-e.1a
TCGA-BQ-7048-01A-11R-1965-07	KIRP	39	3	58	0,0029	poor	PRCC T2	Type 2 Papillary RCC	P-e.2
TCGA-EV-5902-01A-11R-1592-07	KIRP	0	0	100	0,0007	good	PRCC Unc	Unclassified Papillary RCC	P-e.1a
TCGA-DZ-6135-01A-11R-1965-07	KIRP	0	0	100	0,0001	good	PRCC Unc	Unclassified Papillary RCC	P-e.1a
TCGA-A4-8516-01A-11R-2404-07	KIRP	0	0	100	0,0001	good	PRCC T1	Type 1 Papillary RCC	P-e.1a
TCGA-IZ-A6M8-01A-11R-A31O-07	KIRP	0	0	100	0,0001	good	PRCC T1	Type 1 Papillary RCC	P-e.1b
TCGA-GL-A59R-01A-11R-A26U-07	KIRP	0	0	100	0,0017	good	PRCC T1	Type 1 Papillary RCC	P-e.1a
TCGA-5P-A9KE-01A-11R-A42S-07	KIRP	0	0	100	0,0001	good	PRCC Unc	Unclassified Papillary RCC	P-e.1b
TCGA-2Z-A9JD-01A-11R-A42S-07	KIRP	0	0	100	0,0001	good	PRCC T2	Type 2 Papillary RCC	P-e.2
TCGA-HE-A5NJ-01A-11R-A26U-07	KIRP	0	0	100	0,0001	good	PRCC T1	Type 1 Papillary RCC	P-e.1a
TCGA-B9-5156-01A-01R-1592-07	KIRP	0	0	100	0,0001	good	PRCC T1	Type 1 Papillary RCC	P-e.1b
TCGA-B9-4617-01A-01R-1193-07	KIRP	0	0	100	0,0233	good	PRCC T1	Type 1 Papillary RCC	P-e.1a
TCGA-Y8-A898-01A-11R-A355-07	KIRP	0	0	100	0,0022	good	PRCC T1	Type 1 Papillary RCC	P-e.1b
TCGA-BQ-5885-01A-11R-1592-07	KIRP	0	0	100	0,0001	good	PRCC Unc	Unclassified Papillary RCC	P-e.2
TCGA-B3-3926-01A-02R-1351-07	KIRP	4	2	94	0,0083	good	PRCC Unc	Unclassified Papillary RCC	P-e.1a
TCGA-UZ-A9Q1-01A-11R-A42S-07	KIRP	2	1	97	0,0009	good	PRCC T1	Type 1 Papillary RCC	P-e.2
TCGA-AL-3473-01A-01R-1193-07	KIRP	85	0	15	0,0013	intermediate	PRCC T2	Type 2 Papillary RCC	CC-e.3

TCGA-B1-A654-01A-11R-A31O-07	KIRP	0	0	100	0,0001	good	PRCC T1	Type 1 Papillary RCC	P-e.1a
TCGA-G7-6795-01A-11R-1965-07	KIRP	0	0	100	0,0005	good	PRCC T1	Type 1 Papillary RCC	P-e.1b
TCGA-B9-A44B-01A-11R-A24Z-07	KIRP	24	1	75	0,0001	poor	PRCC T2	Type 2 Papillary RCC	P.CIMP-e
TCGA-P4-A5E8-01A-11R-A28H-07	KIRP	38	0	62	0,0206	poor	KIRP CIMP	Type 2 Papillary RCC	P.CIMP-e
TCGA-DW-7839-01A-11R-2139-07	KIRP	0	0	100	0,0023	good	PRCC T1	Type 1 Papillary RCC	P-e.1a
TCGA-SX-A7SQ-01A-12R-A36F-07	KIRP	0	0	100	0,0001	good	PRCC T1	Type 1 Papillary RCC	P-e.1a
TCGA-2Z-A9J5-01A-21R-A38C-07	KIRP	3	0	97	0,0001	good	PRCC T1	Type 1 Papillary RCC	P-e.1b
TCGA-B9-7268-01A-11R-A32Z-07	KIRP	0	0	100	0,0009	good	PRCC T1	Type 1 Papillary RCC	P-e.1a
TCGA-2Z-A9JN-01A-21R-A42S-07	KIRP	5	6	89	0,008	good	PRCC T2	Type 2 Papillary RCC	mixed
TCGA-GL-7773-01A-11R-A32Z-07	KIRP	2	0	98	0,0001	good	PRCC T1	Type 1 Papillary RCC	P-e.1a
TCGA-B1-A656-01A-11R-A31O-07	KIRP	15	2	83	0,0001	intermediate	PRCC T2	Type 2 Papillary RCC	P-e.2
TCGA-2Z-A9JP-01A-11R-A42S-07	KIRP	0	0	100	0,0001	good	PRCC T1	Type 1 Papillary RCC	P-e.1a
TCGA-BQ-7053-01A-11R-1965-07	KIRP	0	0	100	0,0009	good	PRCC T2	Type 2 Papillary RCC	P-e.1b
TCGA-P4-A5E7-01A-31R-A28H-07	KIRP	3	0	97	0,0001	good	PRCC T2	Type 2 Papillary RCC	P-e.1a
TCGA-MH-A562-01A-11R-A26U-07	KIRP	0	0	100	0,0001	good	PRCC T1	Type 1 Papillary RCC	P-e.1b
TCGA-2Z-A9JS-01A-21R-A42S-07	KIRP	0	0	100	0,0043	good	PRCC T1	Type 1 Papillary RCC	P-e.1a
TCGA-P4-A5E6-01A-11R-A28H-07	KIRP	0	0	100	0,0002	good	PRCC T2	Type 2 Papillary RCC	P-e.1b
TCGA-Y8-A8S1-01A-11R-A37K-07	KIRP	26	4	70	0,0019	poor	PRCC Unc	Unclassified Papillary RCC	P-e.2
TCGA-P4-A5ED-01A-11R-A28H-07	KIRP	24	2	74	0,0297	poor	PRCC T2	Type 2 Papillary RCC	P-e.1a
TCGA-A4-A48D-01A-11R-A24Z-07	KIRP	0	0	100	0,0002	good	PRCC T2	Type 2 Papillary RCC	P-e.1b
TCGA-B9-A69E-01A-11R-A31O-07	KIRP	45	3	52	0,002	poor	PRCC T2	Type 2 Papillary RCC	P-e.2
TCGA-A4-8098-01A-11R-2404-07	KIRP	22	1	77	0,0074	poor	PRCC T2	Type 2 Papillary RCC	P-e.1a
TCGA-SX-A71W-01A-12R-A355-07	KIRP	0	0	100	0,0012	good	PRCC T1	Type 1 Papillary RCC	P-e.1a
TCGA-2Z-A9JL-01A-11R-A42S-07	KIRP	0	0	100	0,0001	good	PRCC T1	Type 1 Papillary RCC	P-e.1a
TCGA-G7-A4TM-01A-11R-A31O-07	KIRP	0	0	100	0,0001	good	PRCC T1	Type 1 Papillary RCC	P-e.1a
TCGA-HE-7129-01A-11R-1965-07	KIRP	0	0	100	0,0001	good	PRCC T2	Type 2 Papillary RCC	P-e.1a
TCGA-J7-8537-01A-11R-2404-07	KIRP	23	1	76	0,0038	poor	PRCC T2	Type 2 Papillary RCC	P-e.2
TCGA-4A-A93W-01A-11R-A37K-07	KIRP	0	0	100	0,0002	good	PRCC T1	Type 1 Papillary RCC	P-e.1a
TCGA-A4-8630-01A-11R-2404-07	KIRP	0	0	100	0,0014	good	PRCC T1	Type 1 Papillary RCC	P-e.1a
TCGA-A4-A6HP-01A-11R-A31O-07	KIRP	0	0	100	0,0004	good	PRCC T1	Type 1 Papillary RCC	P-e.1b
TCGA-B3-8121-01A-21R-2404-07	KIRP	8	0	92	0,0001	good	PRCC Unc	Unclassified Papillary RCC	P-e.1b
TCGA-A4-A772-01A-11R-A33Z-07	KIRP	0	0	100	0,0001	good	PRCC T1	Type 1 Papillary RCC	P-e.1b
TCGA-UZ-A9PR-01A-11R-A42S-07	KIRP	0	0	100	0,0002	good	PRCC T1	Type 1 Papillary RCC	P-e.1a
TCGA-F9-A4JJ-01A-11R-A24Z-07	KIRP	43	2	55	0,0009	poor	KIRP CIMP	Type 2 Papillary RCC	P.CIMP-e
TCGA-DZ-6132-01A-11R-1965-07	KIRP	4	0	96	0,0001	good	PRCC Unc	Unclassified Papillary RCC	P-e.1a
TCGA-B9-4113-01A-01R-1193-07	KIRP	0	0	100	0,0001	good	PRCC T1	Type 1 Papillary RCC	P-e.1b
TCGA-GL-A9DC-01A-11R-A37K-07	KIRP	0	0	100	0,0165	good	PRCC T1	Type 1 Papillary RCC	P-e.1a
TCGA-G7-7502-01A-11R-2204-07	KIRP	0	0	100	0,0001	good	PRCC T1	Type 1 Papillary RCC	P-e.1b
TCGA-2Z-A9JO-01A-11R-A42S-07	KIRP	29	5	66	0,0011	poor			P-e.2
TCGA-5P-A9K6-01A-11R-A42S-07	KIRP	0	0	100	0,0001	good	PRCC T1	Type 1 Papillary RCC	P-e.1b
TCGA-B1-A47O-01A-11R-A24Z-07	KIRP	2	0	98	0,0001	good			P-e.1b
TCGA-5P-A9KA-01A-11R-A42S-07	KIRP	0	0	100	0,0001	good	PRCC T2	Type 2 Papillary RCC	P-e.1a
TCGA-2Z-A9J7-01A-11R-A38C-07	KIRP	0	0	100	0,0001	good	PRCC T1	Type 1 Papillary RCC	P-e.1a
TCGA-UZ-A9PX-01A-11R-A42S-07	KIRP	0	0	100	0,0001	good	PRCC T1	Type 1 Papillary RCC	P-e.1a

TCGA-A4-7915-01A-11R-2204-07	KIRP	54	3	43	0,0223	poor	KIRP CIMP	Type 2 Papillary RCC	P.CIMP-e
TCGA-GL-A9DD-01A-11R-A37K-07	KIRP	0	0	100	0,0001	good	PRCC T1	Type 1 Papillary RCC	P-e.1b
TCGA-IA-A40U-01A-11R-A24Z-07	KIRP	6	0	94	0,0001	good	PRCC Unc	Unclassified Papillary RCC	P-e.1b
TCGA-IA-A40X-01A-11R-A24Z-07	KIRP	1	0	99	0,004	good	PRCC T1	Type 1 Papillary RCC	P-e.1a
TCGA-2K-A9WE-01A-11R-A38C-07	KIRP	6	0	94	0,0001	good	PRCC T1	Type 1 Papillary RCC	P-e.1b
TCGA-AT-A5NU-01A-11R-A28H-07	KIRP	0	0	100	0,0001	good	PRCC T1	Type 1 Papillary RCC	P-e.1a
TCGA-AL-7173-01A-11R-2139-07	KIRP	8	0	92	0,0001	good	PRCC T1	Type 1 Papillary RCC	P-e.1a
TCGA-DW-7840-01A-11R-A32Z-07	KIRP	0	0	100	0,0001	good	PRCC T1	Type 1 Papillary RCC	P-e.1a
TCGA-5P-A9KC-01A-11R-A42S-07	KIRP	5	1	94	0,0011	good	PRCC T1	Type 1 Papillary RCC	P-e.2
TCGA-4A-A93Y-01A-11R-A37K-07	KIRP	60	6	34	0,0004	poor	PRCC T1	Type 1 Papillary RCC	P-e.2
TCGA-2Z-A9J1-01A-11R-A38C-07	KIRP	0	0	100	0,0001	good	PRCC T1	Type 1 Papillary RCC	P-e.1a
TCGA-5P-A9KH-01A-11R-A42S-07	KIRP	0	100	0	0,0001	good			Ch-e
TCGA-DZ-6133-01A-11R-1965-07	KIRP	0	0	100	0,0001	good	PRCC Unc	Unclassified Papillary RCC	P-e.1a
TCGA-O9-A75Z-01A-11R-A33Z-07	KIRP	0	0	100	0,0014	good	PRCC T1	Type 1 Papillary RCC	P-e.1a
TCGA-MH-A855-01A-11R-A355-07	KIRP	1	0	99	0,0014	good	PRCC T1	Type 1 Papillary RCC	P-e.2
TCGA-B1-A47N-01A-11R-A24Z-07	KIRP	0	0	100	0,0018	good	PRCC T1	Type 1 Papillary RCC	P-e.1a
TCGA-4A-A93X-01A-11R-A37K-07	KIRP	49	2	49	0,0038	poor	PRCC T2	Type 2 Papillary RCC	P.CIMP-e
TCGA-G7-6793-01A-11R-1965-07	KIRP	74	3	23	0,0002	intermediate	KIRP CIMP	Type 2 Papillary RCC	P.CIMP-e
TCGA-KV-A74V-01A-11R-A33Z-07	KIRP	0	0	100	0,0001	good	PRCC T1	Type 1 Papillary RCC	P-e.1a
TCGA-HE-A5NL-01A-11R-A26U-07	KIRP	0	0	100	0,0001	good	PRCC T1	Type 1 Papillary RCC	P-e.1b
TCGA-P4-AAVM-01A-11R-A42S-07	KIRP	0	0	100	0,0001	good	PRCC T1	Type 1 Papillary RCC	P-e.1a
TCGA-BQ-5886-01A-11R-1592-07	KIRP	0	0	100	0,0001	good	PRCC Unc	Unclassified Papillary RCC	P-e.1b
TCGA-UZ-A9PU-01A-11R-A42S-07	KIRP	0	0	100	0,0001	good	PRCC T1	Type 1 Papillary RCC	P-e.1a
TCGA-UZ-A9PK-01A-11R-A38C-07	KIRP	0	0	100	0,0001	good	PRCC T1	Type 1 Papillary RCC	P-e.1a
TCGA-SX-A7SO-01A-11R-A355-07	KIRP	16	1	83	0,0004	intermediate	PRCC Unc	Unclassified Papillary RCC	P-e.2
TCGA-KV-A6GD-01A-11R-A31O-07	KIRP	5	0	95	0,0001	good	PRCC T2	Type 2 Papillary RCC	P-e.1b
TCGA-B9-A5W7-01A-11R-A31O-07	KIRP	0	0	100	0,0001	good	PRCC T1	Type 1 Papillary RCC	P-e.1a
TCGA-UZ-A9PO-01A-11R-A38C-07	KIRP	0	0	100	0,0001	good	PRCC T1	Type 1 Papillary RCC	P-e.1b
TCGA-Y8-A897-01A-11R-A36F-07	KIRP	0	0	100	0,0016	good	PRCC T1	Type 1 Papillary RCC	P-e.1b
TCGA-DW-7837-01A-11R-2139-07	KIRP	0	0	100	0,0001	good	PRCC T1	Type 1 Papillary RCC	P-e.1b
TCGA-GL-8500-01A-11R-2404-07	KIRP	0	0	100	0,0132	good	PRCC T2	Type 2 Papillary RCC	P-e.1a
TCGA-HE-7128-01A-11R-1965-07	KIRP	0	0	100	0,0001	good	PRCC T2	Type 2 Papillary RCC	P-e.1b
TCGA-G7-A8LE-01A-11R-A36F-07	KIRP	0	0	100	0,0007	good	PRCC T1	Type 1 Papillary RCC	P-e.1a
TCGA-BQ-7061-01A-11R-1965-07	KIRP	13	0	87	0,0001	intermediate	PRCC T2	Type 2 Papillary RCC	P-e.2
TCGA-BQ-7060-01A-11R-1965-07	KIRP	0	0	100	0,0001	good	PRCC T1	Type 1 Papillary RCC	P-e.1b
TCGA-DW-5560-01A-01R-1592-07	KIRP	0	0	100	0,0004	good	PRCC T1	Type 1 Papillary RCC	P-e.1a
TCGA-B1-A47M-01A-11R-A24Z-07	KIRP	0	0	100	0,0002	good	PRCC T1	Type 1 Papillary RCC	P-e.1b
TCGA-BQ-5883-01A-11R-1592-07	KIRP	22	11	67	0,3347	poor	PRCC T2	Type 2 Papillary RCC	mixed
TCGA-MH-A856-01A-11R-A355-07	KIRP	9	0	91	0,0022	good	PRCC T1	Type 1 Papillary RCC	P-e.1a
TCGA-BQ-5890-01A-11R-1592-07	KIRP	16	0	84	0,0004	intermediate	PRCC T2	Type 2 Papillary RCC	P-e.2
TCGA-G7-A8LC-01A-11R-A36F-07	KIRP	0	0	100	0,0084	good	PRCC T2	Type 2 Papillary RCC	P-e.1a
TCGA-2Z-A9JI-01A-11R-A42S-07	KIRP	3	1	96	0,0008	good	PRCC T2	Type 2 Papillary RCC	P-e.1a
TCGA-A4-7286-01A-11R-A32Z-07	KIRP	0	0	100	0,0001	good	PRCC Unc	Unclassified Papillary RCC	P-e.1b
TCGA-2Z-A9JJ-01A-11R-A42S-07	KIRP	11	1	88	0,0088	intermediate	PRCC Unc	Unclassified Papillary RCC	P-e.1a

TCGA-2Z-A9JQ-01A-11R-A42S-07	KIRP	0	0	100	0,0037	good	PRCC T1	Type 1 Papillary RCC	P-e.1a
TCGA-BQ-5884-01A-11R-1592-07	KIRP	10	1	89	0,0012	good	PRCC T2	Type 2 Papillary RCC	P-e.2
TCGA-UN-AAZ9-01A-11R-A38C-07	KIRP	5	0	95	0,0043	good	PRCC T1	Type 1 Papillary RCC	P-e.1a
TCGA-A4-8312-01A-11R-2404-07	KIRP	6	15	79	0,016	good	PRCC T2	Type 2 Papillary RCC	mixed
TCGA-5P-A9JU-01A-11R-A42S-07	KIRP	19	0	81	0,0001	intermediate	PRCC T2	Type 2 Papillary RCC	P-e.1a
TCGA-B9-4115-01A-01R-1193-07	KIRP	0	0	100	0,0008	good	PRCC T1	Type 1 Papillary RCC	P-e.1b
TCGA-5P-A9KF-01A-11R-A42S-07	KIRP	0	0	100	0,0014	good	PRCC T1	Type 1 Papillary RCC	P-e.1a
TCGA-HE-A5NH-01A-11R-A26U-07	KIRP	0	0	100	0,0001	good	PRCC T1	Type 1 Papillary RCC	P-e.1a
TCGA-A4-8310-01A-11R-2404-07	KIRP	0	0	100	0,0001	good	PRCC Unc	Unclassified Papillary RCC	P-e.1a
TCGA-A4-7732-01A-11R-2139-07	KIRP	0	0	100	0,0001	good	PRCC T1	Type 1 Papillary RCC	P-e.1a
TCGA-SX-A7SS-01A-11R-A36F-07	KIRP	0	0	100	0,0203	good	PRCC T1	Type 1 Papillary RCC	P-e.1a
TCGA-A4-7828-01A-11R-2139-07	KIRP	93	1	6	0,0519	intermediate			mixed
TCGA-GL-A9DE-01A-11R-A37K-07	KIRP	5	0	95	0,0007	good	PRCC T2	Type 2 Papillary RCC	P-e.2
TCGA-5P-A9JY-01A-11R-A42S-07	KIRP	0	0	100	0,01	good	PRCC T1	Type 1 Papillary RCC	P-e.1a
TCGA-B1-A657-01A-11R-A31O-07	KIRP	0	0	100	0,0001	good	PRCC Unc	Unclassified Papillary RCC	P-e.1b
TCGA-Q2-A5QZ-01A-11R-A28H-07	KIRP	18	2	80	0,0011	intermediate	PRCC T2	Type 2 Papillary RCC	P.CIMP-e
TCGA-P4-A5EB-01A-11R-A28H-07	KIRP	0	0	100	0,0001	good	PRCC T2	Type 2 Papillary RCC	P-e.1a
TCGA-EV-5903-01A-11R-1592-07	KIRP	1	0	99	0,0002	good	PRCC T1	Type 1 Papillary RCC	P-e.1a
TCGA-BQ-5876-01A-11R-1592-07	KIRP	8	1	91	0,0012	good	PRCC T2	Type 2 Papillary RCC	P-e.2
TCGA-G7-6789-01A-11R-1965-07	KIRP	53	2	45	0,0024	poor	PRCC T2	Type 2 Papillary RCC	P.CIMP-e
TCGA-J7-A8I2-01A-12R-A36F-07	KIRP	79	10	11	0,0001	intermediate			mixed
TCGA-IA-A83W-01A-11R-A355-07	KIRP	0	1	99	0,0001	good	PRCC T1	Type 1 Papillary RCC	P-e.1b
TCGA-B3-A6W5-01A-12R-A33Z-07	KIRP	0	0	100	0,0001	good	PRCC T1	Type 1 Papillary RCC	P-e.1a
TCGA-BQ-7050-01A-11R-1965-07	KIRP	0	0	100	0,0001	good	PRCC T2	Type 2 Papillary RCC	P-e.2
TCGA-5P-A9K9-01A-11R-A42S-07	KIRP	14	0	86	0,0005	intermediate	PRCC T2	Type 2 Papillary RCC	P-e.2
TCGA-SX-A7SU-01A-11R-A36F-07	KIRP	0	0	100	0,0072	good	PRCC T1	Type 1 Papillary RCC	P-e.1a
TCGA-MH-A560-01A-11R-A26U-07	KIRP	6	2	92	0,0001	good	PRCC T2	Type 2 Papillary RCC	P-e.2
TCGA-BQ-5894-01A-11R-1592-07	KIRP	55	2	43	0,017	poor	KIRP CIMP	Type 2 Papillary RCC	P.CIMP-e
TCGA-2Z-A9JM-01A-12R-A42S-07	KIRP	0	0	100	0,0001	good	PRCC T1	Type 1 Papillary RCC	P-e.1b
TCGA-DW-7838-01A-11R-2139-07	KIRP	6	0	94	0,0001	good	PRCC T1	Type 1 Papillary RCC	P-e.1b
TCGA-BQ-5893-01A-11R-1592-07	KIRP	47	1	52	0,0045	poor	KIRP CIMP	Type 2 Papillary RCC	P.CIMP-e
TCGA-HE-7130-01A-11R-1965-07	KIRP	81	6	13	0,0049	intermediate			mixed
TCGA-MH-A561-01A-11R-A26U-07	KIRP	0	0	100	0,0001	good	PRCC T1	Type 1 Papillary RCC	P-e.1a
TCGA-P4-AAVK-01A-11R-A42S-07	KIRP	7	1	92	0,0001	good	PRCC T2	Type 2 Papillary RCC	P-e.2
TCGA-B3-4103-01A-02R-1351-07	KIRP	0	0	100	0,0001	good	PRCC Unc	Unclassified Papillary RCC	P-e.1b
TCGA-G7-A8LD-01A-11R-A36F-07	KIRP	3	0	97	0,0002	good	PRCC T1	Type 1 Papillary RCC	P.CIMP-e
TCGA-A4-7584-01A-11R-2139-07	KIRP	0	0	100	0,0001	good	PRCC T1	Type 1 Papillary RCC	P-e.1a
TCGA-5P-A9K2-01A-11R-A42S-07	KIRP	0	0	100	0,0028	good	PRCC T1	Type 1 Papillary RCC	P-e.1a
TCGA-GL-7966-01A-11R-2204-07	KIRP	46	4	50	0,0009	poor	KIRP CIMP	Type 2 Papillary RCC	P.CIMP-e
TCGA-BQ-7045-01A-31R-1965-07	KIRP	4	0	96	0,0001	good	PRCC T2	Type 2 Papillary RCC	P-e.1b
TCGA-BQ-5887-01A-11R-1965-07	KIRP	70	3	27	0,0002	intermediate	PRCC T2	Type 2 Papillary RCC	P-e.2
TCGA-DW-7836-01A-11R-2139-07	KIRP	0	0	100	0,0001	good	PRCC T1	Type 1 Papillary RCC	P-e.1a
TCGA-F9-A8NY-01A-11R-A36F-07	KIRP	43	3	54	0,0056	poor	KIRP CIMP	Type 2 Papillary RCC	P.CIMP-e
TCGA-BQ-5892-01A-11R-1592-07	KIRP	67	1	32	0,0022	poor	PRCC T2	Type 2 Papillary RCC	CC-e.1

TCGA-AL-3472-01A-01R-1193-07	KIRP	0	0	100	0,0008	good	PRCC T1	Type 1 Papillary RCC	P-e.1a
TCGA-EV-5901-01A-11R-1592-07	KIRP	0	0	100	0,0001	good	PRCC T1	Type 1 Papillary RCC	P-e.1a
TCGA-GL-A59T-01A-21R-A28H-07	KIRP	0	0	100	0,0001	good	PRCC T1	Type 1 Papillary RCC	P-e.1b
TCGA-UZ-A9PP-01A-11R-A42S-07	KIRP	0	0	100	0,0002	good	PRCC T1	Type 1 Papillary RCC	P-e.1a
TCGA-P4-A5EA-01A-11R-A28H-07	KIRP	43	6	51	0,0151	poor	KIRP CIMP	Type 2 Papillary RCC	P.CIMP-e
TCGA-J7-6720-01A-11R-2139-07	KIRP	0	0	100	0,0031	good	PRCC T1	Type 1 Papillary RCC	P-e.1b
TCGA-BQ-7055-01A-11R-1965-07	KIRP	1	99	0	0,0001	good	PRCC T2	Type 2 Papillary RCC	Ch-e
TCGA-HE-A5NI-01A-11R-A26U-07	KIRP	3	0	97	0,0002	good	PRCC T1	Type 1 Papillary RCC	P-e.1a
TCGA-A4-7585-01A-11R-2139-07	KIRP	46	3	51	0,0024	poor	PRCC T2	Type 2 Papillary RCC	P-e.2
TCGA-A4-7288-01A-11R-A32Z-07	KIRP	0	0	100	0,0004	good	PRCC T2	Type 2 Papillary RCC	P-e.1a
TCGA-B3-4104-01A-02R-1351-07	KIRP	80	6	14	0,0004	intermediate			mixed
TCGA-A4-8515-01A-11R-2404-07	KIRP	0	0	100	0,0001	good	PRCC T1	Type 1 Papillary RCC	P-e.1a
TCGA-KO-8414-01A-11R-2315-07	KICH	1	99	0	0,0004	good	ChRCC		Ch-e
TCGA-KO-8417-01A-11R-2315-07	KICH	0	100	0	0,0015	good	ChRCC		Ch-e
TCGA-KL-8343-01A-11R-2315-07	KICH	0	100	0	0,0001	good	ChRCC		Ch-e
TCGA-KN-8427-01A-11R-2315-07	KICH	71	2	27	0,276	intermediate	ChRCC	Metabolically Divergent (MD-)ChRCC	CC-e.3
TCGA-KL-8328-01A-11R-2315-07	KICH	0	100	0	0,0002	good	ChRCC		Ch-e
TCGA-KN-8437-01A-11R-2315-07	KICH	34	25	41	0,8397	poor	ChRCC		mixed
TCGA-KL-8333-01A-11R-2315-07	KICH	5	95	0	0,0008	good	ChRCC		Ch-e
TCGA-KN-8424-01A-11R-2315-07	KICH	1	99	0	0,0001	good	ChRCC		Ch-e
TCGA-KN-8419-01A-11R-2315-07	KICH	3	97	0	0,0001	good	ChRCC		Ch-e
TCGA-KL-8339-01A-11R-2315-07	KICH	0	100	0	0,0001	good	ChRCC		Ch-e
TCGA-KN-8431-01A-11R-2315-07	KICH	1	99	0	0,0007	good	ChRCC		Ch-e
TCGA-KN-8436-01A-11R-2315-07	KICH	4	96	0	0,0003	good	ChRCC		Ch-e
TCGA-KL-8334-01A-11R-2315-07	KICH	0	100	0	0,0001	good	ChRCC		Ch-e
TCGA-KL-8326-01A-11R-2315-07	KICH	0	100	0	0,0006	good	ChRCC		Ch-e
TCGA-KL-8338-01A-11R-2315-07	KICH	0	99	1	0,0002	good	ChRCC		Ch-e
TCGA-KN-8430-01A-11R-2315-07	KICH	0	100	0	0,0007	good	ChRCC		Ch-e
TCGA-KO-8416-01A-11R-2315-07	KICH	0	100	0	0,0013	good	ChRCC		Ch-e
TCGA-KO-8409-01A-11R-2315-07	KICH	0	100	0	0,0002	good	ChRCC		Ch-e
TCGA-KM-8639-01A-11R-2403-07	KICH	15	56	29	0,6922	intermediate	ChRCC		mixed
TCGA-KO-8403-01A-11R-2315-07	KICH	0	100	0	0,0001	good	ChRCC		Ch-e
TCGA-KL-8340-01A-11R-2315-07	KICH	0	100	0	0,0001	good	ChRCC		Ch-e
TCGA-KN-8426-01A-11R-2315-07	KICH	1	98	1	0,0001	good	ChRCC		Ch-e
TCGA-KO-8410-01A-11R-2315-07	KICH	0	100	0	0,0002	good	ChRCC		Ch-e
TCGA-KL-8325-01A-11R-2315-07	KICH	0	100	0	0,0001	good	ChRCC		Ch-e
TCGA-KL-8335-01A-11R-2315-07	KICH	0	100	0	0,0001	good	ChRCC		Ch-e
TCGA-KM-8440-01A-11R-2315-07	KICH	0	100	0	0,0001	good	ChRCC		Ch-e
TCGA-KL-8344-01A-11R-2315-07	KICH	13	85	2	0,0036	intermediate	ChRCC		Ch-e
TCGA-KO-8404-01A-11R-2315-07	KICH	94	6	0	0,0051	intermediate	ChRCC	Metabolically Divergent (MD-)ChRCC	CC-e.3
TCGA-KO-8406-01A-11R-2315-07	KICH	0	100	0	0,0001	good	ChRCC		Ch-e
TCGA-KL-8329-01A-11R-2315-07	KICH	0	100	0	0,0008	good	ChRCC		Ch-e
TCGA-KM-8443-01A-11R-2315-07	KICH	0	100	0	0,0001	good	ChRCC		Ch-e
TCGA-KL-8327-01A-11R-2315-07	KICH	0	100	0	0,0012	good	ChRCC		Ch-e

TCGA-KN-8432-01A-11R-2315-07	KICH	0	100	0	0,0014	good	ChRCC	Ch-e
TCGA-KN-8428-01A-11R-2315-07	KICH	2	97	1	0,0001	good	ChRCC	Ch-e
TCGA-KM-8441-01A-11R-2315-07	KICH	28	56	16	0,5697	poor	ChRCC	mixed
TCGA-KL-8341-01A-11R-2315-07	KICH	0	100	0	0,0013	good	ChRCC	Ch-e
TCGA-KN-8418-01A-11R-2315-07	KICH	0	100	0	0,0003	good	ChRCC	Ch-e
TCGA-KO-8415-01A-11R-2315-07	KICH	3	96	1	0,0001	good	ChRCC	Ch-e
TCGA-KO-8411-01A-11R-2315-07	KICH	0	100	0	0,0018	good	ChRCC	Ch-e
TCGA-KM-8438-01A-11R-2315-07	KICH	7	93	0	0,0041	good	ChRCC	Ch-e
TCGA-KO-8405-01A-11R-2315-07	KICH	2	96	2	0,0001	good	ChRCC	Ch-e
TCGA-KL-8342-01A-11R-2315-07	KICH	2	98	0	0,0008	good	ChRCC	Ch-e
TCGA-KL-8332-01A-11R-2315-07	KICH	3	97	0	0,0001	good	ChRCC	Ch-e
TCGA-KM-8477-01A-11R-2315-07	KICH	0	100	0	0,0001	good	ChRCC	Ch-e
TCGA-KL-8324-01A-11R-2315-07	KICH	0	100	0	0,0063	good	ChRCC	Ch-e
TCGA-KN-8429-01A-11R-2315-07	KICH	0	100	0	0,0001	good	ChRCC	Ch-e
TCGA-KL-8323-01A-21R-2315-07	KICH	1	99	0	0,0001	good	ChRCC	Ch-e
TCGA-KL-8331-01A-11R-2315-07	KICH	0	100	0	0,0001	good	ChRCC	Ch-e
TCGA-KN-8433-01A-11R-2315-07	KICH	3	97	0	0,0032	good	ChRCC	Ch-e
TCGA-KM-8439-01A-11R-2315-07	KICH	14	55	31	0,7414	intermediate	ChRCC	mixed
TCGA-KL-8345-01A-11R-2315-07	KICH	0	100	0	0,0001	good	ChRCC	Ch-e
TCGA-KO-8407-01A-11R-2315-07	KICH	2	98	0	0,0001	good	ChRCC	Ch-e
TCGA-KL-8330-01A-11R-2315-07	KICH	0	100	0	0,0024	good	ChRCC	Ch-e
TCGA-KN-8435-01A-11R-2315-07	KICH	0	100	0	0,0035	good	ChRCC	Ch-e
TCGA-KN-8423-01A-11R-2315-07	KICH	0	100	0	0,0004	good	ChRCC	Ch-e
TCGA-KM-8476-01A-11R-2315-07	KICH	0	100	0	0,0011	good	ChRCC	Ch-e
TCGA-KN-8421-01A-11R-2315-07	KICH	0	100	0	0,0027	good	ChRCC	Ch-e
TCGA-KM-8442-01A-11R-2315-07	KICH	0	100	0	0,0001	good	ChRCC	Ch-e
TCGA-KL-8337-01A-11R-2315-07	KICH	0	99	1	0,0001	good	ChRCC	Ch-e
TCGA-KL-8346-01A-11R-2315-07	KICH	0	100	0	0,0005	good	ChRCC	Ch-e
TCGA-KN-8425-01A-11R-2315-07	KICH	0	100	0	0,0001	good	ChRCC	Ch-e
TCGA-KO-8413-01A-11R-2315-07	KICH	0	96	4	0,0001	good	ChRCC	Ch-e
TCGA-KN-8434-01A-11R-2315-07	KICH	0	100	0	0,0001	good	ChRCC	Ch-e
TCGA-KO-8408-01A-11R-2315-07	KICH	1	99	0	0,0012	good	ChRCC	Ch-e
TCGA-KL-8336-01A-11R-2315-07	KICH	0	100	0	0,0001	good	ChRCC	Ch-e
